# What Can Providers' Motivations Tell Us About Healthcare Quality?

**DOI:** 10.1002/hec.70144

**Published:** 2026-08-03

**Authors:** Lucy Margaret Poole, Line Bjørnskov Pedersen, Anne Sophie Oxholm

**Affiliations:** ^1^ Danish Centre for Health Economics Department of Public Health University of Southern Denmark Odense M Denmark; ^2^ Research Unit of General Practice Department of Public Health University of Southern Denmark Odense M Denmark

**Keywords:** agency theory, altruism, financial motivation, general practice, quality of care

## Abstract

Agency theory suggests that variation in providers' quality of care may be explained by their heterogeneous motivations. We provide novel empirical evidence on the links between different sources of provider motivation and quality of care by combining a unique 4‐year survey dataset on Danish general practitioners' motivations with quality indicators and control variables from rich register data. Using a within‐between regression specification, we estimate the associations between two key sources of motivation (financial motivation and non‐paternalistic altruism toward patients) and seven quality indicators related to accreditation standards in general practice. We find that providers' financial motivation is associated with high‐quality care that relates to remunerated services. We further find that non‐paternalistic altruism toward patients associates with lower‐quality prescribing for drugs that may be highly demanded by patients and fewer annual control visits for chronic disease. These links are mostly found when we compare heterogeneously motivated practices (between estimates) rather than when we look at changes within the same practices over time (within estimates). The findings can help policymakers address agency problems that may arise from patient demands and design policies that motivate delivery of high‐quality care.

## 1 Introduction

At their core, healthcare systems share the common goal of delivering high‐quality care. However, evidence of providers' varying adherence to quality standards and clinical guidelines demonstrates the ongoing difficulties associated with successfully achieving this goal (Gandolfi et al. 2025). To respond to this challenge, policymakers use incentives to motivate providers to deliver high‐quality care. Agency theory suggests that both financial and altruistic motivations influence healthcare providers' provision of care, which may explain variation in their behavior (Ellis and McGuire 1986). Our study aims to provide empirical evidence on the link between providers' motivations and quality of care to better understand how motivations can be used to address agency problems.

Our study adds to the existing empirical investigations that have explored healthcare providers' motivations. This literature can be broadly divided into two strands. The first strand maps heterogeneity in providers' motivations, either by measuring motivation directly using survey data (Fernet et al. 2020; Yordanov et al. 2023) or indirectly through natural and laboratory experiments that investigate providers' responses to incentive schemes (e.g., Godager and Wiesen 2013; Hennig‐Schmidt et al. 2011; Kesternich et al. 2015; Kolstad 2013). Overall, this literature demonstrates that providers' financial and altruistic motivations vary. Most of these studies do not, however, provide direct evidence on whether providers' motivations serve as mechanisms to explain variation in actual behaviors. Exceptions include Brosig‐Koch et al. (2024) and Oxholm et al. (2025), who either use experimental data or focus only on one motivation (intrinsic motivation measured as pure work joy) to uncover such mechanisms. Further empirical evidence on the links between motivations and quality of care is needed to help understand why quality improvement initiatives, such as pay‐for‐performance, fail to consistently induce higher‐quality care (Eijkenaar et al. 2013; Mendelson et al. 2017; Zaresani and Scott 2021).

The second strand of the literature is much less comprehensive and explores how different sources of provider motivation are related to provider behavior and quality of care. In general practice, recent cross‐sectional studies have directly measured general practitioners' (GPs') motivations using survey data and then linked these measures to administrative data on treatment behavior. For example, Oxholm et al. (2024) and Jensen and Andersen (2015) both find altruism toward patients (originally referred to as user orientation) to be associated with higher levels of antibiotic prescribing. Oxholm et al. (2024) further find positive associations between financial motivation (also referred to as extrinsic motivation), consultation volume, and fee‐for‐service revenue per patient. Although most of these outcomes do not directly capture quality, the findings from these studies can provide some insights into how we might expect these motivations to link to proxies for quality of care. Outside of general practice, a few studies have investigated links between provider altruism and outcomes related to patients' mental health treatment (Douven et al. 2019), hospital spending and readmissions (Casalino et al. 2024), patient‐perceived quality (Brock et al. 2016), and physicians' receipt of financial benefits from pharmaceutical companies (Huang et al. 2025). In these studies, altruism is defined as putting the patient's interests above one's own and is elicited through laboratory experiments (Brock et al. 2016; Casalino et al. 2024; Huang et al. 2025) or real‐world responses to a remuneration scheme (Douven et al. 2019). Though the studies investigate very different outcomes, they find that providers' altruism toward patients is associated with higher quality of care.

The existing evidence provides some insights into how providers' motivations may relate to quality, but there are limited studies in this area, particularly in a primary care setting. We aim to fill this gap in the literature by estimating the links between GPs' motivations and quality of care. We focus on two sources of provider motivation: financial motivation (motivation by tangible incentives, including money (Kuvaas et al. 2017)) and patient‐oriented altruism (motivation to do good for specific patients (L. B. Andersen et al. 2011)). In this study, patient‐oriented altruism reflects a non‐paternalistic form of altruism that focuses on maximizing patient utility (satisfaction), recognizing that patient welfare can be broader than just the health benefits they receive from medical care (Farley 1986). We therefore do not capture altruism that drives providers to maximize patient health benefits in instances where this course may not align with patients' preferences. We recognize that patient‐oriented altruism can be defined in several ways (Ellis and McGuire 1986; Farley 1986; Galizzi et al. 2023). The relevance of non‐paternalistic altruism in healthcare settings has been highlighted in other studies (see, e.g., Bergstrom (1982); Ge et al. (2022); Jones‐Lee (1991),  (1992) and Simonsen et al. (2021)).

Our choice to focus only on financial motivation and patient‐oriented altruism is based on both theoretical and empirical considerations. Agency theory traditionally assumes that physicians' behavior is driven by financial and patient‐altruistic concerns (Ellis and McGuire 1986; Farley 1986). Recent studies also suggest that society‐oriented altruism may explain physician behavior, particularly in double‐agency situations where the interests of patients and society do not align (Allen et al. 2022; Oxholm et al. 2024, 2026). As the quality indicators included in this study do not capture notable double agency problems, we do not include this type of motivation. A recent study by Oxholm et al. (2025) identifies links between intrinsic motivation (pure work joy) and quality of care, also in the Danish general practice setting and using the same quality indicators as the ones we consider. We therefore do not include intrinsic motivation to avoid overlap with this study. We instead focus on the two motivations that are highlighted in agency theory and are relevant for our measures of quality of care.

Based on the predictions of agency theory and prior empirical findings, we set general expectations regarding the links between GPs' motivations and quality of care. First, we expect GPs' financial motivation to be positively associated with quality of care when good performance on a quality indicator aligns with revenue‐maximizing behavior. Specifically, we expect to find a positive relationship between financial motivation and quality, when quality is proxied by the provision of directly remunerated services (Oxholm et al. 2024). Highly financially motivated providers may, however, provide less of services that they perceive to be insufficiently remunerated (Doran et al. 2011; Dumont et al. 2008; Skovsgaard et al. 2023). Second, we expect patient‐oriented altruism to be associated with higher quality in instances where there is alignment between patients' demands (which the highly non‐paternalistic patient‐oriented altruistic provider aims to satisfy) and clinical indicators of quality. If patients' demands and clinical guidelines are misaligned, we may instead find negative associations between non‐paternalistic patient‐oriented altruism and quality of care (Jensen and Andersen 2015; Oxholm et al. 2024).

We test these expectations using a unique 4‐year panel of general practices in Denmark. We combine high‐quality register data on process measures of quality of care with the Danish national GP work life survey, which includes items capturing both financial and altruistic motivations (Yordanov et al. 2023; Poole et al. 2025). We employ a within‐between regression specification to benefit from the flexibility of random effects models while also controlling for unobservable, time‐constant heterogeneity among general practices in our within‐practice estimates (Bell and Jones 2015). By adopting this approach, we can infer whether changes in motivation within the same practice over time are associated with changes in quality (within estimates) and how quality varies across practices with heterogeneously motivated GPs (between estimates). A few existing studies have shown that providers' motivations can vary over time (e.g., Attema et al. 2023; Poole et al. 2025). Estimating both within and between relationships provides a more comprehensive understanding of how motivation relates to quality of care.

Our findings largely confirm our expectations. We find that financial motivation relates to higher‐quality care when quality indicators reflect a directly compensated service, for example, preventive home visits and annual control visits for chronic disease. We further find that patient‐oriented altruism relates to lower‐quality prescribing of NSAIDs and to a lesser extent lower‐quality prescribing of sleeping medication, two classes of drugs which may be highly demanded by patients. We also find that patient‐oriented altruism is associated with fewer patients receiving annual control visits for chronic disease. These links between motivations and quality of care are mostly found when we compare heterogeneously motivated practices (between estimates) rather than when we look at changes within the same practices over time (within estimates). Our findings remain robust across a range of supplementary analyses, including different sample definitions, motivation measurements, and time periods.

We make several contributions to the empirical literature linking providers' motivations to their quality of care. First, we directly measure two sources of motivation that are associated with different provider behaviors. As previously mentioned, past studies often elicited provider motivations indirectly based on behavior in natural or laboratory experiments (Brock et al. 2016; Casalino et al. 2024; Douven et al. 2019; Godager and Wiesen 2013). By directly measuring motivation in isolation from provider behavior, we can investigate the underlying mechanisms linking motivation to quality of care. Second, we include four measurement points for all variables. Of the few existing studies linking direct measures of providers' financial motivation and patient‐oriented altruism to their behavior, most are limited to cross‐sectional association designs (Jensen and Andersen 2015; Oxholm et al. 2024). We are therefore able to estimate both within and between relationships. Third, we combine self‐reported survey data on motivation with objectively measured register data on quality of care. We therefore avoid common source bias, which may be present in studies that collect motivation and quality data from a single source (either surveys or experiments) (Jakobsen and Jensen 2015; Veenstra et al. 2022). Fourth, our quality outcomes capture specific dimensions of quality for which unexplained variation has been identified in general practice (see Section 3.1.2). These quality indicators serve as useful tests for the expectations proposed above, and the results may also inform on how to motivate high‐quality care in these clinically relevant areas. Most existing studies have either focused on just a few quality measures (e.g., antibiotics (Jensen and Andersen 2015; Oxholm et al. 2024)) or a single source of provider motivation (e.g., altruism (Casalino et al. 2024; Douven et al. 2019) or intrinsic motivation measured as pure work joy (Oxholm et al. 2025)). By investigating two key sources of motivation and a large set of quality indicators, we broaden the scope of the literature linking provider motivations to quality of care.

The remainder of the paper will proceed as follows. Section 2 introduces the setting of Danish general practice and provides context for institutional changes occurring during the study period. Section 3 describes the data and empirical strategy. Section 4 presents the results, and Section 5 concludes with a discussion of the findings and their implications for policy.

## 2 Institutional Setting

Denmark has a universal, tax‐funded healthcare system in which general practice plays a central role. For most patients, preventive care, diagnostics, and treatment are all handled at the level of the GP. When secondary services are needed, GPs assume a gatekeeping role and connect patients to specialist providers. Only around 10% of all contacts with GPs are estimated to include a referral to another provider (Haastrup et al. 2025). The GP gatekeeping system promotes continuity of care by fostering ongoing relationships between GPs and their patients. Further, this model aims to reduce congestion in emergency departments and ration access to costly secondary services.

The vast majority of GPs in Denmark are self‐employed and work on contract for one of the five Danish regions, with new contracts negotiated jointly across regions every 3 years (Haastrup et al. 2025). Because GPs typically own their practices, they can choose to work alone or in partnership with other GPs. In 2025, the average number of GPs working in a practice was 2.2 (PLO 2025). While GPs do have some flexibility in how they set up their practices, all practices organize their everyday operations and their remuneration around a patient list system. Under this scheme, approximately 98% of the Danish population is enlisted with a specific general practice where they can consult a GP without any copayment (with only a few exceptions for non‐essential services). Patients can choose to be listed with any GP who is accepting new patients and is located within a 15‐km radius of their home (5 km in the capital) (Birk et al. 2024). However, practices have the option to close their list to new patients once the average number of patients per GP exceeds 1600 (K. M. Pedersen et al. 2012). During our analysis period, approximately 60% of general practices had closed their list to new patients (Olsen et al. 2023), and some parts of the country were designated as being at‐risk of a GP shortage (Danish Regions 2018). In combination, these factors suggest that competition for patients by general practices was low during this period.

GP remuneration combines capitation (a risk‐adjusted payment for each patient on the list) with fee‐for‐service, covering around 40% and 60% of total remuneration, respectively (PLO 2025). Practices' total revenue therefore depends not only on the number of patients on their list, but also on their patient case‐mix and the number of services they provide. The fee‐for‐service scheme covers GP contacts (whether in person, virtual, or at the patient's home), laboratory tests, and other diagnostic and preventive services. GPs do not receive a fee for issuing prescriptions or for referring patients to other providers.

Our study period covers the years 2019–2023 and includes the COVID‐19 pandemic, which led to several immediate changes in the working conditions in general practice. At the onset of the pandemic in the spring of 2020, for example, much of GPs' work shifted from in‐person to virtual, and both providers and patients had to adjust to new ways of delivering and receiving care. By early 2022, all COVID‐related restrictions in the healthcare system were lifted, and the situation was no longer considered socially critical (Statens Serum Institut 2022). Besides these pandemic‐related changes, we are unaware of other major institutional changes in general practice taking place during our study period.

## 3 Methods

### 3.1 Data

#### 3.1.1 Measuring Motivation

Data on providers' motivations comes from the Danish national GP work life survey, conducted annually from 2019 to 2022. Each year, all GPs in Denmark who were registered with a provider number received invitations by mail to take part in an online survey. Initial invitations were sent out in May/June each year, with data collection lasting until December. The survey is not publicly accessible, but additional information on its design, distribution procedure, and content is provided in Yordanov et al. (2023) and Poole et al. (2025).

We focus on two sources of provider motivation: financial motivation and patient‐oriented altruism. Using validated survey items that have been previously used to quantify healthcare providers' motivations, we measure GPs' extrinsic motivation (a proxy for their financial motivation) and patient orientation (a proxy for their patient‐oriented altruism) (Jensen and Andersen 2015; Oxholm et al. 2024; Poole et al. 2025; Yordanov et al. 2023). Patient orientation captures a non‐paternalistic altruism that is focused on maximizing patients' utility. Survey items for each motivation include, for example: “If I had been offered better pay, I would have done a better job” (extrinsic motivation) and “it gives me energy to know that I helped the patient” (patient orientation). The complete list of survey items can be found in Supporting Information S1: Appendix A. GPs respond to each statement using a 5‐step Likert scale that ranges from “completely disagree” to “completely agree”. They are also given the option to respond with “do not know/do not want to answer”.

Following completion of the survey by GPs, we generate sum scores for each source of motivation. These scores are calculated by assigning a point value to each Likert scale response, such that the response indicating the highest level of motivation is assigned 5 points and the response indicating the lowest level of motivation 1 point. Answers in the category “do not know/do not want to answer” are imputed with the sample mean for that year (1.33% of all responses for financial motivation, 0.46% of all responses for patient‐oriented altruism). We then sum up these point values for the statements relating to each source of motivation (four items for financial motivation, three items for patient‐oriented altruism) to create two distinct scores. Following other studies on provider motivation, we then standardize the scores using a minimum‐maximum approach for ease of interpretation (Oxholm et al. 2024; Poole et al. 2025; Yordanov et al. 2023). The standardization process ensures that each motivation is measured on a scale ranging from zero (the least motivated GPs) to one (the most motivated GPs). Lastly, we compute average GP motivation scores across GPs who work in the same practice. These measures can therefore be interpreted as the motivation of the average GP in each practice, and they can then be linked to our quality indicators, which are measured at the practice level (see Section 3.1.2).

Before beginning our analyses, we test the fit of our proposed motivation measurement model whereby each survey item loads onto one (and only one) of the two sources of motivation. The results from our confirmatory factor analysis support the validity of our model and its invariance over time (see Supporting Information S1: Appendix B). We are therefore reassured that the survey items adequately capture the two latent constructs of motivation throughout the study period.

#### 3.1.2 Measuring Quality of Care

Quality of care can be measured along different dimensions and in many different ways (Campbell et al. 2000). We proxy quality of care using seven quality indicators highlighted in a recent study by L. B. Pedersen et al. (2024), who evaluated the impact of accreditation of Danish general practices on their quality of care. The quality indicators were related to new accreditation standards and were chosen based on their clinical relevance for general practice, their accessibility in Danish register data, and the fact that they are not self‐reported by the practices (L. B. Pedersen et al. 2024). We include similar process‐based quality indicators that capture providers' prescribing behaviors and their provision of services. Process measures are useful because they are likely closer linked to provider behavior than outcome measures, which may to a larger extent rely on patient characteristics, behavior, and uncertainty (Donabedian 1988). Additionally, the provision of the services captured by these process indicators has been shown to vary across general practices (Koefoed et al. 2013; Prior et al. 2022; Ribe et al. 2021), making them well suited to the estimation of potential links between different sources of motivation and quality of care.

All of our quality indicators are measured in the first 6 months of each year 2020–2023. We include four indicators on providers' prescribing behaviors. These indicators are based on data from the Danish National Prescription Register (LMDB), which contains patient‐level data on all prescriptions redeemed by patients in community pharmacies in Denmark (Pottegård et al. 2016). From this register we calculate “the share of enlisted patients over 65 years old who are polypharmacy patients (> 5 concurrent prescriptions)” and “the average number of prescriptions issued to enlisted patients over 65 years old”. Among older adults, polypharmacy is the strongest predictor of inappropriate prescribing and may therefore suggest lower‐quality care (Carey et al. 2008). We also investigate the prescription of two specific classes of drugs: non‐steroidal anti‐inflammatory drugs (NSAIDs) and sleeping medication. Both are considered potentially inappropriate medications in the pharmacology literature and there exists considerable variation in the prescription of these drugs in general practice (Ribe et al. 2021). NSAIDs can be particularly problematic for older adults when they are not prescribed alongside a proton‐pump inhibitor (PPI), as PPIs reduce the likelihood of gastrointestinal complications due to prolonged NSAID use (Wolfe et al. 1999). Therefore, we include as proxies for quality “the average number of defined daily doses (DDDs) prescribed for NSAIDs (without a PPI prescription) for enlisted patients over 65” and “the average number of DDDs of sleeping medication prescribed for enlisted adult patients (18 years old and up)”. For all quality indicators related to prescribing, lower values reflect higher quality of care.

We include three indicators on the provision of remunerated services. These indicators are based on data from the Danish National Health Insurance Service Register (SSR), which contains information on publicly funded primary care services, including consultations, diagnostic, and curative services in general practice (J. S. Andersen et al. 2011). Following L. B. Pedersen et al. (2024), we calculate “the share of enlisted patients over 75 years old receiving a preventive home visit” and “the share of enlisted patients 18 years and up receiving an annual control visit for chronic disease”. These services support access to and continuity of care for potentially vulnerable populations (Prior et al. 2022). Finally, we calculate “the share of enlisted COPD and asthma patients 18 years and up who receive a spirometry test”. Spirometry testing is an important tool for diagnosing and monitoring both COPD and asthma. Because patients can receive these tests in either general practice or in hospital, we combine SSR general practice data with hospital records in the Danish National Patient Register (LPR) to capture whether the necessary care was delivered. For all three quality indicators related to remunerated services, higher values reflect higher quality of care.

#### 3.1.3 Measuring Patient, Practice, and Area Characteristics

We aim to control for patient, practice, and area characteristics that may potentially impact the relationships between motivations and quality of care. We proxy enlisted patients' socioeconomic status using indicators inspired by the Danish Deprivation Index, which aims to identify patients in need of greater care in general practice (Oxholm et al. 2024; Vedsted and Sørensen 2009). We obtain information on patients' socioeconomic status from Statistics Denmark's registers. We proxy enlisted patients' health status using the Charlson Comorbidity Index (CCI) (Quan et al. 2011), which is based on hospital diagnoses from the past 5 years that are recorded in LPR (as diagnoses are unavailable in primary care data). A more detailed description of these patient characteristics is provided in the descriptive statistics table in Supporting Information S1: Appendix C. Practice characteristics include the number of enlisted patients and the region where the practice is located, both of which are found in the Danish Provider Register. We add two variables related to the area where the practice is located: the degree of urbanization as defined by Statistics Denmark (Statistics Denmark 2025) and whether the area has been identified by the Danish Regions as being at risk of a GP shortage (Danish Regions 2018).

### 3.2 Empirical Approach

We estimate the relationships between practices' motivations and their quality of care using an unbalanced panel of general practices in Denmark. The panel includes all practices that are open (as classified by the Danish Provider Register) and active (as indicated by their patient list size) throughout the study period and whose GP(s) responded to the work life survey at least once between 2019 and 2022. Recall from Section 3.1.1 that motivation data was collected in the second half of each year. To mitigate concerns about reverse causality (namely, that prior performance on quality may impact providers' motivation), we measure practices' quality of care afterward (but close) to their motivation measurement—specifically in the first half of each calendar year. Accordingly, for each cohort we link motivation measured in the second half of year *t‐1* (2019–2022) to practice‐level quality indicators measured in the first half of the following year *t* (2020–2023). This ensures that motivation measurement precedes the quality outcomes while remaining close in timing.

We model the relationship between practices' motivations and quality of care using a within‐between specification. This model specification benefits from the flexibility of random effects models while still allowing for correlation to exist between unobserved heterogeneity and the covariates. Mundlak (1978) showed that random and fixed effects models would produce the same within estimates if this correlation was properly parameterized, and Wooldridge (2019) extends this theory to the case of unbalanced panels. In practice, this parameterization is typically done by incorporating unit averages of time‐varying covariates into a random effects model specification. The within‐between specification (sometimes called a hybrid model (Allison 2009)) is an equivalent but alternative parameterization of the original Mundlak model (see, e.g., Bell and Jones (2015); Snijders and Bosker (2012)), where time‐varying covariates are demeaned to reduce collinearity, improve efficiency, and allow for direct interpretation of the coefficient estimates $\beta_{w}$ and $\beta_{B}$ as the within and between estimates, respectively (Bell and Jones 2015; Raudenbush 1989).

We specify the following within‐between regression model (Bell and Jones 2015):

(1) $$Q_{it}^{k}=\alpha +\beta_{w}\left(M_{it-1}-\bar{M_{i}}\right)+\beta_{B}\bar{M_{i}}+\gamma_{w}\left(X_{it}-\bar{X_{i}}\right)+\gamma_{B}\bar{X_{i}}+\delta Z_{i}+\omega_{it},$$

where $Q_{it}^{k}$ measures practice *i*'s performance on quality indicator *k* at time *t* (first half of 2020, 2021, 2022, or 2023). $M_{it-1}$ is a vector of time‐varying lagged motivation variables measuring average financial motivation and patient‐oriented altruism in practice *i* at time *t‐1* (second half of 2019, 2020, 2021, or 2022). $\bar{M_{i}}$ is a vector of practice *i*'s average level of each source of motivation across the entire motivation measurement period (2019–2022). $X_{it}$ is a vector of time‐varying covariates (list size per GP, enlisted patients' socioeconomic statuses (see Supporting Information S1: Appendix C for details), enlisted patients' health statuses proxied by the Charlson Comorbidity Index (CCI), and a set of time dummies) observed in practice *i* at time *t*. $\bar{X_{i}}$ are the within‐practice averages of these covariates across time. $Z_{i}$ is a vector of time‐invariant covariates (practice location characteristics including region, degree of urbanization, and area at risk of GP shortage), α is a global intercept, and $\omega_{it}$ is a composite error term that includes the practice‐specific effect (after controlling for correlation between the error term and the explanatory variables) and an idiosyncratic error.

Our estimates of interest are $\beta_{w}$ and $\beta_{B}$. The within estimate, $\beta_{w}$, represents how a change in motivation within a practice over time is related to quality of care. The between estimate, $\beta_{B}$, represents how differences in motivation across practices are related to quality of care. A strength of the within estimate is that it controls for time‐invariant unobservable factors. However, we are careful not to interpret any of the estimates as causal relationships between motivation and quality of care. For the estimates to be interpreted causally, the assumption of conditional mean independence must hold for our measures of motivation. While we include a rich set of controls to mitigate concerns about omitted variable bias, we cannot rule out the presence of unobserved confounders. For example, motivation may be correlated with GPs' own or their family members' health state or other unobserved characteristics that impact quality of care, thereby biasing the estimates. Such biases may arise both in the between estimates (across practices) and within estimates (over time).

We estimate Equation (1) separately for each quality indicator *k* as a mixed‐effects model using maximum likelihood estimation with standard errors clustered at the practice level. To account for the fact that our calculation of the average GP motivation scores only includes the motivations of GPs who respond to the survey, we weight observations according to the share of GPs working in the practice who responded to the work life survey in that particular year. We therefore assume that the average motivation sum scores of practices with high survey response rates are more representative of the motivational composition of all GPs working in the practice than the sum scores of practices with low response rates.

### 3.3 Supplementary Analyses

We perform several supplementary analyses alongside our main findings. First, we test the representativity of the sample of general practices included in the study by comparing their performance on quality indicators and control variables to the entire population of active general practices in Denmark. We continue to investigate the robustness of our choice of sample by (i) weighting practices according to their inverse probability of having responded to the survey in a given year based on their observable characteristics, with weights estimated by logistic regression (Supporting Information S1: Appendix G), (ii) looking only at practices who had at least one responding GP in all 4 years the survey was administered (balanced panel), in case practices being included in the study at different times is driving our original findings (Supporting Information S1: Appendix H), and (iii) looking only at solo practices and practices where 100% of GPs responded to the survey, to more strongly link GP‐level motivation with practice‐level quality of care measures (Supporting Information S1: Appendix I).

Second, we evaluate whether our results are robust to alternate ways of measuring motivation: (i) we weight the motivation sum scores by the survey items' factor loadings in the confirmatory factor analysis (Supporting Information S1: Appendix J), (ii) we dichotomize motivation scores at the 50th and 75th percentile of the practice‐level motivation distribution to allow for a non‐linear relationship between motivations and quality of care (Supporting Information S1: Appendix K and Appendix L), and (iii) we treat “do not know/do not want to answer” responses as missing values rather than replacing them with the sample mean to ensure that these responses are not driving our findings (Supporting Information S1: Appendix M). To investigate correlation between the two measures of motivation, we also calculate Pearson correlation coefficients for each year of the study period.

Third, we investigate how the timing of our measures of motivation and quality and care might influence the results. To assess the extent to which identified relationships between motivations and quality are driven by the COVID‐19 pandemic, we drop a wave of observations from the study sample, first using the (i) 2021 year dummy (observations with 2021 quality data and 2020 motivation data, Supporting Information S1: Appendix N) and then using the (ii) 2020 year dummy (observations with 2020 quality data and 2019 motivation data, Supporting Information S1: Appendix O). We also test a non‐lagged model specification whereby motivations and quality of care are measured at the same time (the latter half of each year 2019–2022) rather than measuring quality just after motivations are surveyed (Supporting Information S1: Appendix P).

Fourth, we examine the role of observable patient, practice, and area characteristics by estimating (i) an unadjusted model that includes only the motivations and time dummies as control variables (Appendix Q) and (ii) the original model with patient characteristics excluded, in case both providers' motivations and their quality of care affect the characteristics of their patient list (Supporting Information S1: Appendix R). We also include two sets of results where the dataset has been pre‐processed using entropy balancing. We calculate weights such that the means and variances of all observable characteristics are equalized between practices with above‐median motivation and practices with below‐median motivation (Hainmueller 2012). We perform this pre‐processing separately for financial motivation and patient‐oriented altruism. We then combine these balancing weights with the existing weights (the share of GPs in the practice who responded to the survey) and obtain two new sets of within‐between regression results, the first where covariates are balanced based on providers' financial motivation (Supporting Information S1: Appendix S), and the second where they are balanced based on their patient‐oriented altruism (Supporting Information S1: Appendix T).

As a final robustness check, we consider an alternative measure of NSAID prescribing quality that reflects up‐to‐date guidelines for prescribing these medications in general practice (Danish Society of General Practice 2021). We calculate “the share of patients at high risk for upper gastrointestinal bleeding who are prescribed NSAIDs without a PPI” using an individual‐level algorithm that categorizes patients' risk level based on past diagnoses and medication use (Supporting Information S1: Appendix U) (Petersen et al. 2020).

## 4 Results

### 4.1 Descriptive Statistics

Descriptive statistics for provider motivation (lagged), quality indicators, and control variables related to patient, practice, and area characteristics are presented in Supporting Information S1: Appendix C for each year of the study period. At baseline, the average level of financial motivation in a practice is 0.47, and the average level of patient‐oriented altruism is 0.66. As highlighted in Poole et al. (2025), patient‐oriented altruism increases in 2020 (2021 lagged motivation) and remains at this higher level for the rest of the study period. Financial motivation decreases slightly in 2021 (2022 lagged motivation) and then moves back up toward its original level in 2022 (2023 lagged motivation). We find that the two sources of motivation are only weakly correlated with correlation coefficients below 0.15 in each year (see Supporting Information S1: Appendix D).

At baseline, the average number of prescriptions issued to enlisted patients over 65 is approximately 2.8 prescriptions, with around 15% of these patients classified as polypharmacy patients, on average (see Supporting Information S1: Appendix C). The average number of DDDs prescribed to the target groups is around 3 DDDs per patient for both NSAIDs without PPI and sleeping medication. It appears as though the prescription of sleeping medication is increasing over time, while the prescription of NSAIDs without PPI is decreasing. However, it is important to note that we cannot infer on the statistical significance of the change presented in this descriptive table. In the first half of 2020, on average, practices are providing home visits to around 5% of enlisted patients aged 75 years and above and annual control visits for chronic disease to around 14% of their enlisted adult patients. On average, approximately 11% of COPD and asthma patients received a spirometry test in the first half of 2020. The practice‐level average provision of annual controls and spirometry tests appears to increase during the study period, while the provision of home visits remains fairly stable.

The representativity tests in Supporting Information S1: Appendix E compare the practices included in the study sample with the entire population of active general practices in Denmark in each year of the study period. In terms of their performance on the quality indicators, the practices in the sample are representative of the population on most indicators. The sample does, however, perform slightly better on the NSAIDs indicator (all years), the annual control visit for chronic disease indicator (all years), and spirometry testing (2022 and 2023) compared to the general practice population. We also find some statistically significant but small differences between the sample and the entire practice population for enlisted patients' socioeconomic statuses, but not for their health statuses. Practices in urban areas, areas at risk of a GP shortage, and in the North Denmark Region, Region of Southern Denmark, and Region Zealand are well represented in all waves. Practices in rural areas and in the Central Denmark Region are slightly overrepresented while practices in the Capital Region of Denmark are underrepresented.

### 4.2 Regression Results

Table 1 presents the regression results for Equation (1) investigating the relationships between provider motivations and quality of care. For simplicity, we include only the coefficient estimates for financial motivation and patient‐oriented altruism. The complete table including time dummies and controls can be found in Supporting Information S1: Appendix F. When interpreting the coefficient estimates, recall that improvements in quality related to prescribing (columns 1–4) are reflected by negative coefficient estimates, while improvements in service provision (columns 5–7) are reflected by positive coefficient estimates (see Section 3.1.2).

**TABLE 1:** Regression results for within‐between specification.

	(1)	(2)	(3)	(4)	(5)	(6)	(7)
	Average number of prescriptions (over 65)	Share of polypharmacy patients (over 65)	Average DDDs, NSAIDs without PPI (over 65)	Average DDDs, sleeping medication (18+)	Share of patients with preventive home visit (75+)	Share of patients with annual control for chronic disease (18+)	Share of COPD and asthma patients with spirometry test (18+)
Lagged financial motivation (within)	−0.01470 (0.01597)	−0.00084 (0.00275)	−0.05807 (0.13395)	−0.34126*** (0.12750)	0.00358 (0.00410)	0.01137*** (0.00389)	−0.00323 (0.00528)
Lagged patient‐oriented altruism (within)	0.01763 (0.02132)	−0.00115 (0.00339)	−0.14668 (0.17991)	0.04335 (0.15865)	0.00520 (0.00737)	0.00742 (0.00583)	0.00562 (0.00749)
Lagged financial motivation (between)	−0.02767 (0.03492)	−0.00413 (0.00406)	0.53100** (0.25196)	0.29482 (0.27252)	0.02629*** (0.00773)	0.02611*** (0.00728)	0.00460 (0.00779)
Lagged patient‐oriented altruism (between)	0.05063 (0.06297)	0.00849 (0.00704)	1.86519*** (0.42806)	0.70762* (0.42802)	−0.01980* (0.01110)	−0.03830*** (0.01307)	−0.01959 (0.01220)
Constant	2.09436***	0.07248***	−1.91187***	2.21504***	0.04893***	0.13567***	0.11803***
	(0.07678)	(0.00888)	(0.55208)	(0.59570)	(0.01346)	(0.01669)	(0.01509)
Observations	3286	3286	3286	3286	3286	3286	3286
No. of practices	1248	1248	1248	1248	1248	1248	1248

*Note:* The columns contain coefficients from within‐between regressions estimated by maximum likelihood. Each of the seven quality indicators is measured for 6 months in a given year and regressed on 6‐month lagged period (t‐1) financial motivation, patient‐oriented altruism, time‐dummies (reference year 2023) and a set of control variables related to practice, patient, and area characteristics. Control variables are described in more detail in Table C1 in Supporting Information S1: Appendix C. We present only the estimates related to motivations for simplicity, with the complete table found in Supporting Information S1: Appendix F. The regressions are based on an unbalanced sample of 3286 observations from 1248 general practices in Denmark. The number of observations per wave is 755 (in 2020), 842 (in 2021), 841 (in 2022), and 848 (in 2023). Observations are weighted according to the share of GPs in the practice responding to the motivation survey in that year. Standard errors clustered by practice in parentheses.

* *p* < 0.10.

** *p* < 0.05.

*** *p* < 0.01.

We find statistically significant within estimates ($\beta_{w}$) for the relationship between financial motivation, prescribing of sleeping medication, and the provision of annual control visits for chronic disease. More specifically, a one standard deviation (SD) increase in a practice's average level of financial motivation is associated with a decrease of 0.039 DDDs^1^ of sleeping medication prescribed (6.3% of one SD in sleeping medication prescribing) and an increase of 0.001 in the share of patients receiving an annual control visit (5.6% of one SD). Between practice estimates ($\beta_{B}$) show that a one SD increase in financial motivation is associated with increases of 0.006 and 0.005 in the share of patients receiving a home visit and an annual control visit for chronic disease, respectively. These increases represent 14.6% of one SD of the share of patients receiving a home visit and 9.8% of one SD of the share of patients receiving an annual control for chronic disease. We further find that a one SD increase in financial motivation is associated with a 0.11 DDD increase in the prescription of NSAIDs without PPI between practices (6.3% of one SD).

Changes in patient‐oriented altruism are not statistically significantly associated with changes in quality within practices over time. Between practices, however, we find that patient‐oriented altruism is statistically significantly associated with lower quality of care for some indicators, that is, the prescription of NSAIDs without PPI and the provision of annual control visits for chronic disease. More specifically, a one SD increase in patient‐oriented altruism is associated with an increase of 0.28 DDDs of NSAIDs prescribed (16.1% of one SD) and a decrease of 0.006 in the share of patients receiving an annual control for chronic disease (11.8% of one SD).

### 4.3 Supplementary Analyses

As outlined in Section 3.3, we consider several alternate specifications of our within‐between regression model. Appendices G through U in the supplementary materials present the complete regression results tables for the robustness checks described in Section 3.3.

The statistically significant, positive relationships between financial motivation, the provision of annual control visits for chronic disease, and the provision of home visits to older adults appear largely robust across the alternate models we investigate. The result for financial motivation and prescription of NSAIDs without PPI is also fairly robust. We find that the within estimate for financial motivation and DDDs of sleeping medication is consistent in its direction across all alternate specifications, but it remains statistically significant at the 5% level only in about half of the robustness checks.

The results for patient‐oriented altruism, the prescription of NSAIDs without PPI, and the provision of annual control visits for chronic disease are highly robust across most supplementary analyses. The result for NSAIDs is also robust to an alternate definition of quality in NSAID prescribing (Supporting Information S1: Appendix U). We also find that the estimated relationship between patient‐oriented altruism and the prescription of sleeping medication (between practices) is consistently positive and becomes statistically significant at the 5% level when we apply the balanced panel criterion (Supporting Information S1: Appendix H), remove observations from 2021 (Supporting Information S1: Appendix N), measure motivations and quality at the same time (Supporting Information S1: Appendix P), or use entropy balancing to pre‐process the data (Supporting Information S1: Appendix T). When looking at the balanced panel (Supporting Information S1: Appendix H), we observe some additional statistically significant estimates, in particular for the patient‐oriented altruism between estimates. The between estimates for the balanced panel most likely differ from the unbalanced panel between estimates because they are based on the specific subset of practices that responded to all four work life surveys, which may make them less representative of the full study population. A potential concern for the within estimates in the unbalanced panel case is that panel attrition could introduce bias in these estimates (if practices who drop out of the balanced panel have different trends over time than those who remain in the panel throughout the entire period). Reassuringly, the within estimates are qualitatively similar across the balanced and unbalanced samples, which reduces our concern that selection issues are driving our findings (Wooldridge 2019).

## 5 Discussion

This study estimates the relationships between different sources of provider motivation and quality of care in general practice. The two motivations that we study (financial motivation and patient‐oriented altruism) appear to relate differently to quality, as we observe variation in the estimated direction and magnitude of these relationships. There are several plausible explanations as to why we find different sources of providers' motivation to relate differently to quality of care. In line with our expectations, financial motivation appears to contribute to high‐quality care when the quality indicators are directly linked to providers' remuneration, as is the case with preventive home visits and annual control visits for chronic disease. These findings are supported by Oxholm et al. (2024) who show that GPs' financial motivation is positively associated with the average amount of fee‐for‐service revenue generated per patient. We also find that changes in financial motivation within practices are negatively associated with the prescription of sleeping medication, another indicator of high‐quality care. However, this result is not robust across all of the supplementary analyses we investigate.

We find that patient‐oriented altruism is associated with lower‐quality prescribing of NSAIDs. This finding could be explained by the fact that NSAIDs are a class of drugs that may be highly demanded by patients for pain relief. Given our definition of patient‐oriented altruism as a non‐paternalistic form of altruism whereby providers aim to satisfy patients' demands, this finding aligns with our expectations. We do not find a similarly strong relationship between patient‐oriented altruism and the prescription of sleeping medication (another likely demanded drug), as the coefficient estimate is statistically insignificant in the main model specification. However, across all supplementary analyses (see appendices G through U) the between estimate for patient‐oriented altruism and sleeping medication is consistently positive and becomes statistically significant in some models. These findings align with Jensen and Andersen (2015) and Oxholm et al. (2024) who find that patient‐oriented altruism is associated with lower‐quality prescribing of antibiotics, another class of potentially highly demanded drugs.

We further find that patient‐oriented altruism is negatively associated with the provision of annual control visits for chronic disease between practices. This finding is highly robust across different model specifications. It may be the case that this resource‐intensive, preventive service is not prioritized by highly patient‐altruistic practices, as providers might assess these services to have too high of an opportunity cost to other enlisted patients with more immediate needs for care. Oxholm et al. (2024) show that highly patient‐altruistic practices tend to serve more high‐need patients, while Prior et al. (2022) show that practices serving older patients with more comorbidities provide fewer annual control visits for chronic disease. In combination, these studies support our speculation that highly patient‐altruistic practices may face more immediate demands from complex patients on their list and may therefore deprioritize services like annual control visits.

Our results related to patient‐oriented altruism differ from those in the experimental literature on physician altruism and responses to payment schemes. In this branch of the literature, altruistic behavior is implied by decision‐making that prioritizes patients' health benefits (see, e.g., Godager and Wiesen (2013) and Attema et al. (2023)), which are a single dimension of patients' utility functions. Our definition of patient‐oriented altruism focuses on the provider's desire to maximize the patient's utility (satisfaction) as a whole, which does not always lead to the same result as would health benefit maximization alone. This distinction may explain why we find patient‐oriented altruism (defined in this non‐paternalistic way) to be associated with lower quality of care on indicators where patients' demands are in opposition to clinical guidelines. This comparison underscores that altruism is a multidimensional concept (as also highlighted by Simonsen et al. (2021), Jones‐Lee (1991),  (1992), Bergstrom (1982), and Ge et al. (2022)), and that different definitions may lead to different conclusions regarding the link between provider altruism and quality of care.

A general observation across both sources of motivation is that we observe fewer statistically significant within estimates compared to between estimates. One possible explanation for this finding is that the within relationships are imprecisely estimated due to the relatively small variation in motivations within practices over time (see Supporting Information S1: Appendix C which shows that the within SD of both motivations is smaller than the between‐practice SD). Consequently, even a meaningful association between within‐practice changes in motivation and quality of care may fail to be statistically significant due to low precision. Another potential concern is that the difference in statistical significance between the within and between‐practice estimates is driven by unobservable, time‐constant practice characteristics. Such characteristics are naturally accounted for in the within estimation but not in the between estimation. While we cannot rule out this explanation, we are reassured that the between estimates align with our expectations and that they are robust to the inclusion of a rich set of control variables and to weighting observations using entropy balancing (see Supporting Information S1: Appendices S and T).

This study has several strengths. First, we use a 4‐year panel design that combines unique survey data on motivation with rich register data on quality of care and control variables. This data structure allows us to uncover the links between motivations and quality of care both within practices over time and across practices. Second, we directly measure GPs' financial and patient‐altruistic motivations and then link these motivations to their behavior, rather than indirectly inferring their motivations after their behavior has already been observed (either in laboratory or natural experiments). Third, we consider several process measures of quality that allow us to test our expectations for how motivations and quality might interact. Fourth, we include a rich set of control variables on patient, practice, and area characteristics. By including these variables, we reduce potential omitted variable bias from unobserved factors that likely relate to both providers' motivations and their quality of care. However, we still refrain from interpreting our estimates as causal, as unobserved factors that explain both variation in motivation and in quality of care may remain.

We also face some limitations. First, we do not (and cannot) capture all dimensions of quality. The outcomes we use are focused on clinical guidelines and were created as reasonable proxies for quality in general practice in relation to accreditation standards (L. B. Pedersen et al. 2024), but they cannot capture all the nuance involved with treating patients with these specific medications and services. Other dimensions of quality (patient satisfaction, for example) and their relationships to motivation would be interesting to uncover in future studies. Second, we can only measure quality indicators at the practice level and therefore cannot precisely link individual GP‐level motivation to outcomes. It may be the case that the GPs who respond to the motivation survey are not an accurate representation of all GPs working in their practice (the average practice includes 2.2 GPs), or that changes in which GPs are responding from year to year are diluting the strength of the within estimates. To strengthen the link between motivation and quality, we weight observations according to the share of GPs responding to the survey in the practice. As a robustness check, we also look at the subsample of practices with a 100% survey response rate (including solo GP practices) and find qualitatively similar results. Third, we focus only on two types of motivation: patient‐oriented altruism and financial motivation. As previously detailed, we have chosen this focus due to these motivations' central role in the traditional physician agency theory (Ellis and McGuire 1986; Farley 1986) and their direct relevance for the quality indicators studied. However, we recognize that other motivations may also relate to physicians' care provision, for example society‐oriented altruism (Allen et al. 2022; Oxholm et al. 2024, 2026) and intrinsic motivation (Oxholm et al. 2025).

Fourth, our study period includes the COVID‐19 pandemic, which could have impacted both providers' motivations and their quality of care. The robustness checks in Supporting Information S1: Appendices N and O allow us to investigate whether some of our statistically significant findings may be driven by the pandemic period. We find most results to be robust to specifications that drop observations from the most disruptive pandemic years (2020 and 2021). Fifth, our analysis is based on a sample of practices where at least one GP responded to our survey during the study period. These practices account for 46.3%–54.1% of all general practices in Denmark. It is not possible to know whether the practices whose GPs never respond to the motivation survey systematically differ in their motivations from those who do respond. We assess the representativity of our sample to the best of our ability by comparing quality and control variables for included practices to those of the practice population and find the sample to be representative of the population on several observable characteristics (see Supporting Information S1: Appendix E). In a supplementary analysis, we also find that our results are robust to weighting observations based on the inverse probability of at least one GP responding to the survey (see Supporting Information S1: Appendix G). Finally, though we include a rich set of control variables in our model, we cannot rule out the presence of unobserved confounders. GPs' motivations may be correlated with unobserved characteristics that relate to quality of care, potentially introducing bias into the within and between estimates. We are therefore careful not to interpret our findings as causal.

Our findings on the links between provider motivations and quality of care may have important implications for policy. One already highlighted finding is the few statistically significant within estimates compared to between estimates. This difference may indicate better scope for policymakers to design policies with the heterogeneous motivations of providers in mind, rather than try to influence practices' motivations toward a specific motivational profile that links to desirable behaviors or outcomes. As we find positive associations between providers' financial motivation and quality of care, one effective policy could be to use fees. However, it is important to remember that not all providers are equally motivated by money (Yordanov et al. 2023). Especially since we find that financial motivation and patient‐oriented altruism link to quality in very different ways (in terms of the magnitude and direction of the estimated relationships, and which quality indicators are most relevant), it is evident that a one‐size‐fits‐all approach to motivating high‐quality care will likely not work for all providers. A wider set of incentives that is applied to all providers but is successful in appealing to a broader set of motivational types may help to balance out differences in quality. For example, we find that patient‐oriented altruism links to lower quality of care on some indicators, expectedly because patients' demands do not match clinical guidelines. New initiatives could aim to solve such agency problems through improved communication to patients about the long‐run consequences of their demands for their health.

Overall, this study provides novel empirical evidence of the link between providers' financial and patient‐oriented motivations and quality of care. This evidence suggests that policymakers should carefully consider providers' heterogeneous motivations when designing policies to promote high‐quality healthcare.

## Ethics Statement

The Research & Innovation Organization at the University of Southern Denmark registered the data processing activities (file number 10.482). The Regional Scientific Ethical Committees for Southern Denmark assessed the study and concluded that no further ethical approval was needed, cf. Section 14, subsection 1 in the Act on Research Ethics Review of Health Research Projects (file number 20192000–99).

## Conflicts of Interest

The authors declare no conflicts of interest.

## Supporting information


Supporting Information S1

## Data Availability

Research data are not shared as accessibility to data is restricted by GDPR rules.

## Endnotes

^1^
This is calculated by multiplying the estimated within relationship between financial motivation and the prescribing of sleeping medication (−0.341, see Table 1) by the within standard deviation of financial motivation (0.114, see Supporting Information S1: Appendix C). Afterwards (in the parenthesis), this value (−0.039) is compared to the within standard deviation of sleeping medication prescribing (0.616) to obtain the standardised regression coefficient. Standardisation of the between estimates is done using a similar approach but instead using the baseline between‐practice standard deviations.
