# Supplementary material for: What Can Providers' Motivations Tell Us About Healthcare Quality?
Source: Health Econ. 2026 Aug 3;35(12):1801–13. doi: 10.1002/hec.70144 (PMC13724289; doi:10.1002/hec.70144)
Supplement: Supplementary file 1 [file HEC-35-1801-s001.docx]

**What can providers’ motivations tell us about healthcare quality?**

*Supporting materials*

[Appendix A – Motivation survey items	2](#_Toc230096518)

[Appendix B – Confirmatory factor analysis and measurement equivalence testing	3](#_Toc230096519)

[Appendix C – Descriptive statistics (means and standard deviations) for motivations, quality indicators, and control variables, by year	4](#_Toc230096520)

[Appendix D – Correlation coefficients for financial motivation and patient-oriented altruism	7](#_Toc230096521)

[Appendix E – Representativity tests on quality indicators and control variables	8](#_Toc230096522)

[Appendix F – Regression results for within-between specification (complete version of Table 1)	16](#_Toc230096523)

[Appendix G – Regression results with practices weighted by the inverse probability of responding to the survey	19](#_Toc230096524)

[Appendix H – Regression results on a balanced panel of general practices	22](#_Toc230096525)

[Appendix I – Regression results for the subset of practices with 100% survey response rate	25](#_Toc230096526)

[Appendix J – Regression results with motivation sum scores weighted by CFA factor loadings	28](#_Toc230096527)

[Appendix K – Regression results with motivations dichotomised at the 50th percentile of the distribution	31](#_Toc230096528)

[Appendix L – Regression results with motivations dichotomised at the 75th percentile of the distribution	34](#_Toc230096529)

[Appendix M – Regression results where “do not know/do not want to answer” responses are treated as missing values	37](#_Toc230096530)

[Appendix N – Regression results when observations with 2020 motivation and 2021 quality are removed	40](#_Toc230096531)

[Appendix O – Regression results when observations with 2019 motivation and 2020 quality are removed	43](#_Toc230096532)

[Appendix P – Regression results when motivations and quality of care are measured at the same time	46](#_Toc230096533)

[Appendix Q – Unadjusted regression results with motivations and time dummies as controls	49](#_Toc230096534)

[Appendix R – Regression results when patient characteristics are removed from the model	51](#_Toc230096535)

[Appendix S – Regression results after entropy balancing on financial motivation	53](#_Toc230096536)

[Appendix T – Regression results after entropy balancing on patient-oriented altruism	56](#_Toc230096537)

[Appendix U – Results for an alternative definition of high quality NSAID prescribing	59](#_Toc230096538)

[References	62](#_Toc230096539)

# **Appendix A – Motivation survey items**

The GP survey contains the items listed below for extrinsic motivation and patient orientation, which are used to proxy financial motivation and patient-oriented altruism, respectively. These items have previously been validated in the literature on GP motivation (see e.g. Yordanov et al. (2023) and Oxholm et al. (2024)). GPs respond to each survey item using a 5-step Likert scale ranging from “completely disagree” to “completely agree.” They can also respond with “do not know/do not want to answer.”

Extrinsic motivation (EM)

EM1: If I am supposed to put in extra effort in my job, I need to get extra pay.

EM2: External incentives such as bonuses and provisions are essential for how well I perform my job.

EM3: It is important for me to have an external incentive to strive for in order to do a good job.

EM4: If I had been offered better pay, I would have done a better job.

Patient orientation (PO)

PO1: The individual patient is more important than formal rules.

PO2: It gives me energy to know that I helped the patient.

PO3: If the patient is satisfied, the job is done.

# **Appendix B – Confirmatory factor analysis and measurement equivalence testing**

Before proceeding with regression analyses, we test the fit of the factor structure linking the survey items that proxy financial motivation and patient-oriented altruism to the overall sum scores for each motivational component. Confirmatory factor analysis (CFA) is conducted at the individual GP level to align with the data collection process. Table B1 presents goodness-of-fit statistics from two CFA models: the first (column (1)) imposes the same factor structure between the survey items and the latent motivational constructs in each year (2019 to 2022), while the second (column (2)) adds the additional restriction that the factor loadings for each survey item must be constant across all four waves. Both models show acceptable fit, with RMSEA and SRMR below 0.04 and CFI and TLI above 0.95. The third column gives the p-value from a nested model test comparing the unrestricted configural invariance model to the restricted metric invariance model. The insignificant p-value of 0.147 suggests that imposing the equal factor loading constraint does not significantly worsen the fit of the model. This result supports measurement equivalence in our motivation model throughout the study period.

Table B1. Goodness-of-fit statistics from confirmatory factor analysis and nested model test

|  | Configural invariance model (1) | Metric invariance model (2) | delta  (3) |
| --- | --- | --- | --- |
| Chi-squared | 128 | 148 | 20.68 |
| Degrees of freedom | 56 | 71 | 15 |
| RMSEA | 0.032 | 0.029 |  |
| AIC | 94013 | 94004 |  |
| BIC | 94563 | 94455 |  |
| CFI | 0.990 | 0.990 |  |
| TLI | 0.985 | 0.988 |  |
| SRMR | 0.035 | 0.038 |  |
| p-value from nested model test |  |  | 0.147 |

Table B2. Factor loadings from confirmatory factor analysis

|  | Wave 1 (2019) factor loading |
| --- | --- |
| EM1 | 0.3811 |
| EM2 | 0.8810 |
| EM3 | 0.8125 |
| EM4 | 0.6642 |
| PO1 | 0.4520 |
| PO2 | 0.6280 |
| PO3 | 0.4251 |

Note: Table B2 gives the wave 1 (2019) factor loadings for the individual survey items that proxy financial motivation (EM) and patient-oriented altruism (PO). Based on the result of the nested model test in Table B1 – which shows that applying constant factor loadings across waves does not worsen model fit – wave 1 factor loadings are used to weight survey items in the motivation sum score calculations in all four waves of the study period in a robustness check (see Appendix G).

# **Appendix C – Descriptive statistics (means and standard deviations) for motivations, quality indicators, and control variables, by year**

Table C1. Descriptive statistics (means and standard deviations) for motivations, quality indicators, and control variables at practice level, by year

|  | 2020 | 2021 | 2022 | 2023 | Within S.D. |
| --- | --- | --- | --- | --- | --- |
| **Motivations** |  |  |  |  |  |
| Lagged financial motivation | 0.4741 (0.2101) | 0.4695 (0.2092) | 0.4254 (0.2081) | 0.4475 (0.2182) | 0.1137 |
| Lagged patient-oriented altruism | 0.6627 (0.1483) | 0.7209 (0.1286) | 0.7221 (0.1265) | 0.7168 (0.1290) | 0.0829 |
|  |  |  |  |  |  |
| **Quality indicators** |  |  |  |  |  |
| Average no. of prescriptions (over 65) | 2.8197  (0.2924) | 2.9202 (0.3090) | 2.9907 (0.3080) | 3.0884 (0.3185) | 0.1084 |
| Share of polypharmacy patients (over 65) | 0.1474  (0.0317) | 0.1578 (0.0345) | 0.1654 (0.0344) | 0.1753 (0.0357) | 0.0135 |
| Average DDDs, NSAIDs without PPI (over 65) | 2.8037  (1.7434) | 2.5080 (1.5923) | 2.3785 (1.6066) | 2.2107 (1.5027) | 0.5188 |
| Average DDDs, sleeping medication (18+) | 3.4999  (1.5749) | 3.9241  (1.596) | 4.3805 (1.7529) | 4.7056 (2.4981) | 0.6160 |
| Share of patients with preventive home visit (75+) | 0.0457  (0.0411) | 0.0518 (0.0485) | 0.0484 (0.0435) | 0.0478 (0.0446) | 0.0189 |
| Share of patients with annual control for chronic disease (18+) | 0.1375  (0.0510) | 0.1512 (0.0556) | 0.1610 (0.0550) | 0.1776 (0.0589) | 0.0179 |
| Share of COPD and asthma patients with spirometry test (18+) | 0.1127 (0.0437) | 0.1096 (0.0529) | 0.1435 (0.0550) | 0.1449 (0.0545) | 0.0269 |
|  |  |  |  |  |  |
| **Patient characteristics** |  |  |  |  |  |
| Share of enlisted patients who are 20-59 years old and unemployed for at least six months (SOCIO1) | 0.0132 (0.0059) | 0.0090 (0.0051) | 0.0044 (0.0029) | 0.0054 (0.0031) | 0.0034 |
| Share of enlisted patients who are 25 to 59 years old without vocational education (SOCIO2) | 0.1072 (0.0262) | 0.1060 (0.0261) | 0.1035 (0.0254) | 0.1001 (0.0288) | 0.0037 |
| Share of enlisted patients who are 18 to 59 years old on welfare payments (SOCIO3) | 0.0612 (0.0212) | 0.0600 (0.0209) | 0.0593 (0.0201) | 0.0588 (0.0289) | 0.0023 |
| Share of enlisted patients who are 0 to 16 years old in family with low educational level (SOCIO4) | 0.0107 (0.0064) | 0.0100 (0.0060) | 0.0094 (0.0056) | 0.0084 (0.0051) | 0.0012 |
| Share of enlisted patients who are immigrants and descendants from non-Western countries (SOCIO5) | 0.0833 (0.0774) | 0.0840 (0.0746) | 0.0839 (0.0720) | 0.0850 (0.0677) | 0.0049 |
| Share of enlisted patients who are +30 years old and single (SOCIO6) | 0.1549 (0.0311) | 0.1589 (0.0318) | 0.1606 (0.0321) | 0.1620 (0.0383) | 0.0042 |
| Share of enlisted patients who are +70 years old with a low disposable family income (SOCIO7) | 0.0375 (0.0210) | 0.0383 (0.0206) | 0.0394 (0.0202) | 0.0396 (0.0217) | 0.0026 |
| Share of enlisted patients with Charlson Comorbidity Index equal to 1 (CCI=1) | 0.0379 (0.0090) | 0.0379 (0.0094) | 0.0377 (0.0091) | 0.0369 (0.0086) | 0.0017 |
| Share of enlisted patients with Charlson Comorbidity Index greater than 1 (CCI>1) | 0.0524 (0.0135) | 0.0529 (0.0135) | 0.0535 (0.0135) | 0.0548 (0.0138) | 0.0024 |
|  |  |  |  |  |  |
| **Practice and area characteristics** |  |  |  |  |  |
| Average list size per GP | 1657  (439) | 1659  (437) | 1639  (386) | 1660  (434) | 145 |
| Share of practices located in rural area | 34.17% | 31.35% | 33.53% | 32.19% |  |
| Share of practices in intermediate density area | 31.66% | 33.61% | 32.58% | 33.97% |  |
| Share of practices located in urban area | 34.17% | 35.04% | 33.89% | 33.84% |  |
| Share of practices located in area at risk of GP shortage | 7.68% | 7.84% | 8.32% | 6.96% |  |
| Region  Capital  Zealand  North  South  Central | 27.15%  13.25%  6.62%  24.9%  28.08% | 29.93%  13.78%  7.01%  24.23%  25.06% | 28.18%  12.49%  7.73%  24.14%  27.47% | 30.07%  11.79%  8.25%  24.29%  25.59% |  |
|  |  |  |  |  |  |
| Number of practices | 755 | 842 | 841 | 848 |  |
|  |  |  |  |  |  |

Note: Table C1 gives the mean values for motivations, quality indicators, and control variables by year measured at the practice level. Standard deviations (S.D.) in parentheses. The rightmost column includes the within standard deviation across the whole study period. Lagged motivation variables are measured from June to December in the previous year (2019 to 2022). The motivation variables are based on validated measures from the literature on GP motivation (see e.g. Yordanov et al. (2023) and Oxholm et al. (2024) and Appendix A). Quality variables and controls related to patient, practice, and area characteristics are measured from January to June in each year. The quality indicators are related to accreditation standards in Danish general practice (see Pedersen et al., 2024). The control variables on enlisted patients’ socioeconomic (SOCIO) characteristics are inspired by the Danish Deprivation Index (Vedsted & Sørensen, 2009), whereas enlisted patients’ health characteristics are based on the Charlson Comorbidity Index (CCI) (Quan et al., 2011). Practices located in rural and urban areas are defined according to a population density algorithm from Statistics Denmark (2025) with the baseline group being intermediate density areas (the algorithm takes inspiration from the Eurostat classification ‘Degree of Urbanisation (DEGURBA)’). The Danish Regions (2018) designate areas at risk of GP shortages based on locations where it is difficult to recruit GPs or where patient burdens are unusually high.

# **Appendix D – Correlation coefficients for financial motivation and patient-oriented altruism**

Table D1. Correlation coefficients for financial motivation and patient-oriented altruism

|  | 2020 (lagged) | 2021 (lagged) | 2022 (lagged) | 2023 (lagged) |
| --- | --- | --- | --- | --- |
| Pearson correlation coefficient | 0.1499^*^ | 0.0616 | 0.0337 | 0.0678^*^ |

Note: Pearson correlation coefficients for financial motivation and patient-oriented altruism in each year of the study period.

^*^indicates statistically significant correlation at a 5% significance level.

# **Appendix E – Representativity tests on quality indicators and control variables**

Table E1. Representativity tests on quality indicators and control variables in 2020

| Variable | Sample | | Population | | P-value |
| --- | --- | --- | --- | --- | --- |
|  | Mean | SD | Mean | SD |  |
| **Quality indicators** |  |  |  |  |  |
| Average no. of prescriptions (over 65) | 2.8197 | 0.2924 | 2.8135 | 0.3295 | 0.6561 |
| Share of polypharmacy patients (over 65) | 0.1474 | 0.0317 | 0.1477 | 0.0350 | 0.8698 |
| Average DDDs, NSAIDs without PPI (over 65) | 2.8037 | 1.7434 | 3.1413 | 1.9412 | <0.001 |
| Average DDDs, sleeping medication (18+) | 3.4999 | 1.5749 | 3.5384 | 1.6611 | 0.5917 |
| Share of patients with preventive home visit (75+) | 0.0457 | 0.0411 | 0.0434 | 0.0468 | 0.2356 |
| Share of patients with annual control for chronic disease (18+) | 0.1375 | 0.0510 | 0.1261 | 0.0588 | <0.001 |
| Share of COPD and asthma patients with spirometry test (18+) | 0.1127 | 0.0437 | 0.1101 | 0.0482 | 0.2085 |
|  |  |  |  |  |  |
| **Patient characteristics** |  |  |  |  |  |
| Share of enlisted patients who are 20-59 years old and unemployed for at least six months (SOCIO1) | 0.0132 | 0.0059 | 0.0142 | 0.0065 | <0.001 |
| Share of enlisted patients who are 25 to 59 years old without vocational education (SOCIO2) | 0.1072 | 0.0262 | 0.1116 | 0.0295 | <0.001 |
| Share of enlisted patients who are 18 to 59 years old on welfare payments (SOCIO3) | 0.0612 | 0.0212 | 0.0618 | 0.0229 | 0.5494 |
| Share of enlisted patients who are 0 to 16 years old in family with low educational level (SOCIO4) | 0.0107 | 0.0064 | 0.0110 | 0.0072 | 0.3034 |
| Share of enlisted patients who are immigrants and descendants from non-Western countries (SOCIO5) | 0.0833 | 0.0774 | 0.0930 | 0.0835 | 0.0067 |
| Share of enlisted patients who are +30 years old and single (SOCIO6) | 0.1549 | 0.0311 | 0.1622 | 0.0347 | <0.001 |
| Share of enlisted patients who are +70 years old with a low disposable family income (SOCIO7) | 0.0375 | 0.0210 | 0.0387 | 0.0228 | 0.2091 |
| Share of enlisted patients with Charlson Comorbidity Index equal to 1 (CCI=1) | 0.0379 | 0.0090 | 0.0383 | 0.0095 | 0.3651 |
| Share of enlisted patients with Charlson Comorbidity Index greater than 1 (CCI>1) | 0.0524 | 0.0135 | 0.0525 | 0.0144 | 0.9637 |
|  |  |  |  |  |  |
| **Practice/area characteristics** |  |  |  |  |  |
| Average list size per GP | 1657 | 439 | 1689 | 451 | 0.1065 |
| Share of practices located in rural area | 34.17% |  | 28.06% |  | 0.002 |
| Share of practices in intermediate density area | 31.66% |  |  |  |  |
| Share of practices located in urban area | 34.17% |  | 37.44% |  | 0.123 |
| Share of practices located in area at risk of GP shortage | 7.68% |  | 9.28% |  | 0.201 |
| Region |  |  |  |  |  |
| Capital | 27.15% |  | 36.17% |  | <0.001 |
| Zealand | 13.25% |  | 13.38% |  | 0.926 |
| North | 6.62% |  | 8.33% |  | 0.149 |
| South | 24.9% |  | 21.02% |  | 0.035 |
| Central | 28.08% |  | 21.09% |  | <0.001 |
|  |  |  |  |  |  |
| Number of practices | 755 | | 1632 | |  |
|  |  |  |  |  |  |

Note: Table E1 provides the representativity tests comparing the sample included in the regression analyses (n=755) to the entire population of active general practices in Denmark in 2020 (N=1632). The rightmost column presents the p-value from a two-sided t-test or Chi-difference test comparing the means or proportions of both groups. P-values below 0.05 indicate a statistically significant difference between the study sample and the population of general practices in Denmark in 2020.

Table E2. Representativity tests on quality indicators and control variables in 2021

| Variable | Sample | | Population | | P-value |
| --- | --- | --- | --- | --- | --- |
|  | Mean | SD | Mean | SD |  |
| **Quality indicators** |  |  |  |  |  |
| Average no. of prescriptions (over 65) | 2.9202 | 0.3090 | 2.9281 | 0.3431 | 0.5725 |
| Share of polypharmacy patients (over 65) | 0.1578 | 0.0345 | 0.1591 | 0.0376 | 0.4047 |
| Average DDDs, NSAIDs without PPI (over 65) | 2.5080 | 1.5923 | 2.8030 | 1.7965 | <0.001 |
| Average DDDs, sleeping medication (18+) | 3.9241 | 1.5960 | 4.0646 | 1.9541 | 0.0722 |
| Share of patients with preventive home visit (75+) | 0.0518 | 0.0485 | 0.0502 | 0.0540 | 0.4785 |
| Share of patients with annual control for chronic disease (18+) | 0.1512 | 0.0556 | 0.1424 | 0.0630 | <0.001 |
| Share of COPD and asthma patients with spirometry test (18+) | 0.1096 | 0.0529 | 0.1061 | 0.0542 | 0.1269 |
|  |  |  |  |  |  |
| **Patient characteristics** |  |  |  |  |  |
| Share of enlisted patients who are 20-59 years old and unemployed for at least six months (SOCIO1) | 0.0090 | 0.0051 | 0.0097 | 0.0057 | 0.001 |
| Share of enlisted patients who are 25 to 59 years old without vocational education (SOCIO2) | 0.1060 | 0.0261 | 0.1108 | 0.0317 | <0.001 |
| Share of enlisted patients who are 18 to 59 years old on welfare payments (SOCIO3) | 0.0600 | 0.0209 | 0.0615 | 0.0270 | 0.1186 |
| Share of enlisted patients who are 0 to 16 years old in family with low educational level (SOCIO4) | 0.0100 | 0.0060 | 0.0106 | 0.0070 | 0.0179 |
| Share of enlisted patients who are immigrants and descendants from non-Western countries (SOCIO5) | 0.0840 | 0.0746 | 0.0948 | 0.0854 | 0.002 |
| Share of enlisted patients who are +30 years old and single (SOCIO6) | 0.1589 | 0.0318 | 0.1649 | 0.0382 | <0.001 |
| Share of enlisted patients who are +70 years old with a low disposable family income (SOCIO7) | 0.0383 | 0.0206 | 0.0392 | 0.0228 | 0.3488 |
| Share of enlisted patients with Charlson Comorbidity Index equal to 1 (CCI=1) | 0.0379 | 0.0094 | 0.0382 | 0.0097 | 0.4347 |
| Share of enlisted patients with Charlson Comorbidity Index greater than 1 (CCI>1) | 0.0529 | 0.0135 | 0.0530 | 0.0148 | 0.8584 |
|  |  |  |  |  |  |
| **Practice/area characteristics** |  |  |  |  |  |
| Average list size per GP | 1659 | 437 | 1699 | 495 | 0.0504 |
| Share of practices located in rural area | 31.35% |  | 27.76% |  | 0.062 |
| Share of practices in intermediate density area |  |  |  |  |  |
| Share of practices located in urban area | 35.04% |  | 37.94% |  | 0.157 |
| Share of practices located in area at risk of GP shortage | 7.84% |  | 9.06% |  | 0.310 |
| Region |  |  |  |  |  |
| Capital | 29.93% |  | 36.73% |  | 0.001 |
| Zealand | 13.78% |  | 12.98% |  | 0.583 |
| North | 7.01% |  | 8.3% |  | 0.261 |
| South | 24.23% |  | 21.03% |  | 0.071 |
| Central | 25.06% |  | 20.96% |  | 0.021 |
|  |  |  |  |  |  |
| Number of practices | 842 | | 1621 | |  |
|  |  |  |  |  |  |

Note: Table E2 provides the representativity tests comparing the sample included in the regression analyses (n=842) to the population of general practices in Denmark in 2021 (N=1621). The rightmost column presents the p-value from a two-sided t-test or Chi-difference test comparing the means or proportions of both groups. P-values below 0.05 indicate a statistically significant difference between the study sample and the population of general practices in Denmark in 2021.

Table E3. Representativity tests on quality indicators and control variables in 2022

| Variable | Sample | | Population | | P-value |
| --- | --- | --- | --- | --- | --- |
|  | Mean | SD | Mean | SD |  |
| **Quality indicators** |  |  |  |  |  |
| Average no. of prescriptions (over 65) | 2.9907 | 0.3080 | 2.9739 | 0.3422 | 0.2339 |
| Share of polypharmacy patients (over 65) | 0.1654 | 0.0344 | 0.1642 | 0.0381 | 0.4492 |
| Average DDDs, NSAIDs without PPI (over 65) | 2.3785 | 1.6066 | 2.6428 | 1.7295 | <0.001 |
| Average DDDs, sleeping medication (18+) | 4.3805 | 1.7529 | 4.4290 | 2.1513 | 0.5735 |
| Share of patients with preventive home visit (75+) | 0.0484 | 0.0435 | 0.0471 | 0.0468 | 0.5070 |
| Share of patients with annual control for chronic disease (18+) | 0.1610 | 0.0550 | 0.1515 | 0.0625 | <0.001 |
| Share of COPD and asthma patients with spirometry test (18+) | 0.1435 | 0.0550 | 0.1372 | 0.0569 | 0.0086 |
|  |  |  |  |  |  |
| **Patient characteristics** |  |  |  |  |  |
| Share of enlisted patients who are 20-59 years old and unemployed for at least six months (SOCIO1) | 0.0044 | 0.0029 | 0.0049 | 0.0032 | <0.001 |
| Share of enlisted patients who are 25 to 59 years old without vocational education (SOCIO2) | 0.1035 | 0.0254 | 0.1082 | 0.0310 | <0.001 |
| Share of enlisted patients who are 18 to 59 years old on welfare payments (SOCIO3) | 0.0593 | 0.0201 | 0.0598 | 0.0265 | 0.6255 |
| Share of enlisted patients who are 0 to 16 years old in family with low educational level (SOCIO4) | 0.0094 | 0.0056 | 0.0099 | 0.0067 | 0.0455 |
| Share of enlisted patients who are immigrants and descendants from non-Western countries (SOCIO5) | 0.0839 | 0.0720 | 0.0964 | 0.0851 | <0.001 |
| Share of enlisted patients who are +30 years old and single (SOCIO6) | 0.1606 | 0.0321 | 0.1659 | 0.0374 | <0.001 |
| Share of enlisted patients who are +70 years old with a low disposable family income (SOCIO7) | 0.0394 | 0.0202 | 0.0395 | 0.0214 | 0.8840 |
| Share of enlisted patients with Charlson Comorbidity Index equal to 1 (CCI=1) | 0.0377 | 0.0091 | 0.0376 | 0.0093 | 0.7807 |
| Share of enlisted patients with Charlson Comorbidity Index greater than 1 (CCI>1) | 0.0535 | 0.0135 | 0.0529 | 0.0143 | 0.2510 |
|  |  |  |  |  |  |
| **Practice/area characteristics** |  |  |  |  |  |
| Average list size per GP | 1639 | 386 | 1707 | 511 | <0.001 |
| Share of practices located in rural area | 33.53% |  | 28.3% |  | 0.008 |
| Share of practices in intermediate density area |  |  |  |  |  |
| Share of practices located in urban area | 33.89% |  | 37.74% |  | 0.061 |
| Share of practices located in area at risk of GP shortage | 8.32% |  | 9.28% |  | 0.435 |
| Region |  |  |  |  |  |
| Capital | 28.18% |  | 36.21% |  | <0.001 |
| Zealand | 12.49% |  | 12.73% |  | 0.862 |
| North | 7.73% |  | 9.02% |  | 0.280 |
| South | 24.14% |  | 20.86% |  | 0.064 |
| Central | 27.47% |  | 21.28% |  | 0.001 |
|  |  |  |  |  |  |
| Number of practices | 841 | | 1590 | |  |
|  |  |  |  |  |  |

Note: Table E1 provides the representativity tests comparing the sample included in the regression analyses (n=841) to the population of general practices in Denmark in 2022 (N=1590). The rightmost column presents the p-value from a two-sided t-test or Chi-difference test comparing the means or proportions of both groups. P-values below 0.05 indicate a statistically significant difference between the study sample and the population of general practices in Denmark in 2022.

Table E4. Representativity tests on quality indicators and control variables in 2023

| Variable | Sample | | Population | | P-value |
| --- | --- | --- | --- | --- | --- |
|  | Mean | SD | Mean | SD |  |
| **Quality indicators** |  |  |  |  |  |
| Average no. of prescriptions (over 65) | 3.0884 | 0.3185 | 3.0778 | 0.3370 | 0.4531 |
| Share of polypharmacy patients (over 65) | 0.1753 | 0.0357 | 0.1749 | 0.0377 | 0.7762 |
| Average DDDs, NSAIDs without PPI (over 65) | 2.2107 | 1.5027 | 2.4577 | 1.6591 | <0.001 |
| Average DDDs, sleeping medication (18+) | 4.7056 | 2.4981 | 4.7605 | 2.3337 | 0.5905 |
| Share of patients with preventive home visit (75+) | 0.0478 | 0.0446 | 0.0449 | 0.0441 | 0.1253 |
| Share of patients with annual control for chronic disease (18+) | 0.1776 | 0.0589 | 0.1698 | 0.0656 | 0.0037 |
| Share of COPD and asthma patients with spirometry test (18+) | 0.1449 | 0.0545 | 0.1392 | 0.0574 | 0.0166 |
|  |  |  |  |  |  |
| **Patient characteristics** |  |  |  |  |  |
| Share of enlisted patients who are 20-59 years old and unemployed for at least six months (SOCIO1) | 0.0054 | 0.0031 | 0.0059 | 0.0034 | <0.001 |
| Share of enlisted patients who are 25 to 59 years old without vocational education (SOCIO2) | 0.1001 | 0.0288 | 0.1057 | 0.0306 | <0.001 |
| Share of enlisted patients who are 18 to 59 years old on welfare payments (SOCIO3) | 0.0588 | 0.0289 | 0.0599 | 0.0267 | 0.3393 |
| Share of enlisted patients who are 0 to 16 years old in family with low educational level (SOCIO4) | 0.0084 | 0.0051 | 0.0091 | 0.0062 | 0.0021 |
| Share of enlisted patients who are immigrants and descendants from non-Western countries (SOCIO5) | 0.0850 | 0.0677 | 0.1009 | 0.0879 | <0.001 |
| Share of enlisted patients who are +30 years old and single (SOCIO6) | 0.1620 | 0.0383 | 0.1673 | 0.0376 | 0.0012 |
| Share of enlisted patients who are +70 years old with a low disposable family income (SOCIO7) | 0.0396 | 0.0217 | 0.0399 | 0.0227 | 0.7367 |
| Share of enlisted patients with Charlson Comorbidity Index equal to 1 (CCI=1) | 0.0369 | 0.0086 | 0.0370 | 0.0090 | 0.8574 |
| Share of enlisted patients with Charlson Comorbidity Index greater than 1 (CCI>1) | 0.0548 | 0.0138 | 0.0541 | 0.0146 | 0.2177 |
|  |  |  |  |  |  |
| **Practice/area characteristics** |  |  |  |  |  |
| Average list size per GP | 1660 | 434 | n/a | n/a | n/a |
| Share of practices located in rural area | 32.19% |  | 29.29% |  | 0.139 |
| Share of practices in intermediate density area |  |  |  |  |  |
| Share of practices located in urban area | 33.84% |  | 37.97% |  | 0.044 |
| Share of practices located in area at risk of GP shortage | 6.96% |  | n/a |  | n/a |
| Region |  |  |  |  |  |
| Capital | 30.07% |  | 35.67% |  | 0.005 |
| Zealand | 11.79% |  | 13.27% |  | 0.298 |
| North | 8.25% |  | 9.06% |  | 0.503 |
| South | 24.29% |  | 21.06% |  | 0.068 |
| Central | 25.59% |  | 20.93% |  | 0.009 |
|  |  |  |  |  |  |
| Number of practices | 848 | | 1567 | |  |
|  |  |  |  |  |  |

Note: Table E1 provides the representativity tests comparing the sample included in the regression analyses (n=848) to the population of general practices in Denmark in 2023 (N=1567). The rightmost column presents the p-value from a two-sided t-test or Chi-difference test comparing the means or proportions of both groups. P-values below 0.05 indicate a statistically significant difference between the study sample and the population of general practices in Denmark in 2023. In 2023, we are missing (n/a) population-level data on list size per GP and share of practices located in areas at risk of GP shortages as these statistics were compiled from the GP survey and are only available from 2019 to 2022.

# **Appendix F – Regression results for within-between specification (complete version of Table 1)**

Table F1. Regression results for within-between specification (complete version of Table 1 including estimates for control variables and time dummies)

|  | (1) | (2) | (3) | (4) | (5) | (6) | (7) |
| --- | --- | --- | --- | --- | --- | --- | --- |
|  | Average number of prescriptions (over 65) | Share of polypharmacy patients  (over 65) | Average DDDs, NSAIDs without PPI (over 65) | Average DDDs, sleeping medication (18+) | Share of patients with preventive home visit (75+) | Share of patients with annual control for chronic disease (18+) | Share of COPD and asthma patients with spirometry test (18+) |
|  |  |  |  |  |  |  |  |
| Lagged financial motivation (within) | -0.01470  (0.01597) | -0.00084  (0.00275) | -0.05807  (0.13395) | -0.34126***  (0.12750) | 0.00358  (0.00410) | 0.01137***  (0.00389) | -0.00323  (0.00528) |
|  |  |  |  |  |  |  |  |
| Lagged patient-oriented altruism (within) | 0.01763  (0.02132) | -0.00115  (0.00339) | -0.14668  (0.17991) | 0.04335  (0.15865) | 0.00520  (0.00737) | 0.00742  (0.00583) | 0.00562  (0.00749) |
|  |  |  |  |  |  |  |  |
| Lagged financial motivation (between) | -0.02767  (0.03492) | -0.00413  (0.00406) | 0.53100**  (0.25196) | 0.29482  (0.27252) | 0.02629***  (0.00773) | 0.02611***  (0.00728) | 0.00460  (0.00779) |
|  |  |  |  |  |  |  |  |
| Lagged patient-oriented altruism (between) | 0.05063  (0.06297) | 0.00849  (0.00704) | 1.86519***  (0.42806) | 0.70762*  (0.42802) | -0.01980*  (0.01110) | -0.03830***  (0.01307) | -0.01959  (0.01220) |
|  |  |  |  |  |  |  |  |
| List per GP (within) | 0.00001 | 0.00000 | 0.00002 | 0.00008 | -0.00001 | -0.00001** | -0.00000 |
|  | (0.00001) | (0.00000) | (0.00009) | (0.00010) | (0.00001) | (0.00000) | (0.00000) |
| List per GP (between) | 0.00001 | 0.00000 | 0.00039*** | -0.00016 | 0.00000 | 0.00000 | 0.00000 |
|  | (0.00001) | (0.00000) | (0.00011) | (0.00012) | (0.00000) | (0.00000) | (0.00000) |
| SOCIO1 (within) | 2.27855 | 0.33560 | -3.74230 | -10.36308 | 0.62691 | 0.06350 | 0.58994 |
|  | (1.40142) | (0.21674) | (13.76098) | (9.12466) | (0.43748) | (0.22959) | (0.39162) |
| SOCIO2 (within) | 1.31290* | 0.21609* | -7.65584 | -6.76182 | 0.48240* | -0.13810 | 0.00640 |
|  | (0.79432) | (0.12146) | (7.84139) | (6.13084) | (0.27771) | (0.14643) | (0.24964) |
| SOCIO3 (within) | -2.05773* | -0.30838** | 5.37440 | 8.59306 | -0.35601 | -0.33750 | -0.15377 |
|  | (1.10872) | (0.15676) | (8.57740) | (8.72776) | (0.25483) | (0.21096) | (0.28209) |
| SOCIO4 (within) | -1.56572 | -0.39559 | -23.57813 | -15.46000 | -0.30647 | -0.30606 | -0.70084 |
|  | (2.40908) | (0.35996) | (16.68016) | (16.13112) | (0.63085) | (0.41834) | (0.74057) |
| SOCIO5 (within) | 0.37218 | 0.11164 | 10.49737 | 1.52068 | 0.07893 | -0.06663 | 0.03381 |
|  | (0.58271) | (0.10156) | (6.98474) | (4.57449) | (0.22988) | (0.11963) | (0.15384) |
| SOCIO6 (within) | 0.16047 | -0.08331 | 17.52793*** | 6.00758 | -0.00782 | 0.06616 | -0.07160 |
|  | (0.57259) | (0.08914) | (5.99642) | (5.63166) | (0.19353) | (0.12089) | (0.18965) |
| SOCIO7 (within) | 2.70051*** | 0.36189*** | -7.01660 | 19.20973*** | 0.08654 | 0.26876 | 0.10776 |
|  | (0.75302) | (0.11042) | (6.08207) | (7.40390) | (0.20634) | (0.17542) | (0.25980) |
| CCI = 1 (within) | 2.73505** | 0.28744 | -9.66587 | -16.10065 | -0.09986 | 0.67786*** | 0.61600* |
|  | (1.26152) | (0.19688) | (9.55420) | (11.37930) | (0.27369) | (0.24202) | (0.36535) |
| CCI > 1 (within) | 2.99785*** | 0.46557*** | -22.11885*** | 7.56891 | 0.46941* | 0.22966 | 1.07737*** |
|  | (0.93293) | (0.14705) | (8.31413) | (9.59341) | (0.26196) | (0.20151) | (0.32756) |
| SOCIO1 (between) | -1.76176 | 0.37755 | 4.61368 | -65.92220** | 0.35433 | -1.03891* | 0.33335 |
|  | (3.15191) | (0.36017) | (21.17467) | (29.84832) | (0.56914) | (0.59046) | (0.54774) |
| SOCIO2 (between) | 3.13802*** | 0.31654*** | 14.73740*** | -14.57595*** | 0.14190 | -0.09875 | -0.01722 |
|  | (0.61903) | (0.07205) | (3.99645) | (4.04362) | (0.09281) | (0.12447) | (0.11371) |
| SOCIO3 (between) | 2.41954*** | 0.17775** | -8.46551* | 66.02665*** | -0.15282 | 0.18457 | -0.26963** |
|  | (0.59463) | (0.08416) | (4.83905) | (13.23011) | (0.09935) | (0.12282) | (0.12298) |
| SOCIO4 (between) | 0.54745 | 0.26020 | 31.54214** | -22.99385 | -0.49552 | 0.15190 | 0.09952 |
|  | (2.02839) | (0.23151) | (14.41570) | (23.37256) | (0.32531) | (0.43861) | (0.43015) |
| SOCIO5 (between) | 0.33567* | 0.03008 | -0.84181 | -4.12951*** | 0.05551 | -0.00563 | 0.00284 |
|  | (0.17936) | (0.02016) | (1.18511) | (1.47866) | (0.03764) | (0.03621) | (0.03314) |
| SOCIO6 (between) | -2.17980*** | -0.22674*** | 0.12914 | 8.45614*** | 0.01753 | -0.29551*** | -0.01615 |
|  | (0.35833) | (0.04271) | (2.45753) | (2.91507) | (0.05708) | (0.07532) | (0.07245) |
| SOCIO7 (between) | 3.80403*** | 0.53166*** | 19.92981*** | -12.71862*** | -0.13065 | 0.17493 | 0.24618* |
|  | (0.58916) | (0.07466) | (4.70914) | (4.76598) | (0.09834) | (0.14871) | (0.12806) |
| CCI = 1 (between) | 10.64870*** | 1.15342*** | -7.89174 | 2.27547 | -0.09005 | 1.00830*** | 1.19733*** |
|  | (1.16935) | (0.14445) | (7.54811) | (10.77906) | (0.17073) | (0.27649) | (0.22588) |
| CCI > 1 (between) | 3.18149*** | 0.18768 | 6.72114 | 14.84903 | 0.07969 | 0.83015*** | -0.21996 |
|  | (0.98432) | (0.12984) | (6.55978) | (9.17967) | (0.15537) | (0.23147) | (0.20471) |
| 2020 (within) | -0.27930*** | -0.02915*** | 0.69481*** | -0.92269*** | -0.00552 | -0.03576*** | -0.03010*** |
|  | (0.01419) | (0.00215) | (0.13993) | (0.11062) | (0.00469) | (0.00273) | (0.00465) |
| 2021 (within) | -0.15985*** | -0.01662*** | 0.34846*** | -0.50877*** | 0.00102 | -0.02357*** | -0.03247*** |
|  | (0.00886) | (0.00141) | (0.08046) | (0.07035) | (0.00264) | (0.00181) | (0.00310) |
| 2022 (within) | -0.10713*** | -0.01005*** | 0.17923*** | -0.26735*** | 0.00155 | -0.01556*** | 0.00063 |
|  | (0.00647) | (0.00102) | (0.05598) | (0.05042) | (0.00183) | (0.00124) | (0.00193) |
| 2020 (between) | -0.29840*** | -0.03770*** | 0.80851*** | -0.53267 | -0.00806 | -0.04164*** | -0.04211*** |
|  | (0.04192) | (0.00500) | (0.30456) | (0.35362) | (0.00705) | (0.00881) | (0.00812) |
| 2021 (between) | -0.22945*** | -0.02711*** | 0.46703* | -0.75165*** | 0.00044 | -0.02528*** | -0.03270*** |
|  | (0.03799) | (0.00469) | (0.23932) | (0.24458) | (0.00637) | (0.00805) | (0.00805) |
| 2022 (between) | -0.12682*** | -0.01396*** | 0.31472 | -0.40830 | 0.00154 | -0.01768** | -0.00474 |
|  | (0.03690) | (0.00458) | (0.23604) | (0.25915) | (0.00647) | (0.00775) | (0.00759) |
| Region North | 0.32150*** | 0.03045*** | -0.65919*** | -2.18084*** | -0.01769*** | 0.05284*** | 0.03304*** |
|  | (0.03341) | (0.00410) | (0.21787) | (0.35018) | (0.00486) | (0.00711) | (0.00709) |
| Region Central | 0.31081*** | 0.03222*** | -0.87244*** | -1.33780*** | 0.00132 | 0.03887*** | 0.03346*** |
|  | (0.02632) | (0.00334) | (0.16810) | (0.37805) | (0.00447) | (0.00539) | (0.00553) |
| Region South | 0.09774*** | 0.00776*** | -0.77791*** | -2.49543*** | -0.01336*** | 0.02779*** | 0.02628*** |
|  | (0.02394) | (0.00290) | (0.17129) | (0.30669) | (0.00438) | (0.00563) | (0.00582) |
| Region Zealand | 0.09765*** | 0.01050*** | 0.35078* | -0.67598*** | -0.01797*** | 0.01676*** | -0.00255 |
|  | (0.02369) | (0.00278) | (0.18575) | (0.25428) | (0.00404) | (0.00640) | (0.00543) |
| Rural area | -0.05375*** | -0.00538*** | 0.12867 | 0.09680 | -0.00424 | -0.01031** | -0.00461 |
|  | (0.01697) | (0.00204) | (0.11645) | (0.11869) | (0.00258) | (0.00404) | (0.00399) |
| Urban area | 0.01338 | 0.00108 | 0.19261 | 0.55143*** | 0.00004 | -0.00797* | -0.01444*** |
|  | (0.02012) | (0.00226) | (0.12983) | (0.14622) | (0.00341) | (0.00441) | (0.00423) |
| Risk of GP shortage | -0.07633*** | -0.00904*** | 0.08022 | -0.69138*** | -0.00441 | 0.00986 | 0.00025 |
|  | (0.02769) | (0.00306) | (0.21305) | (0.22590) | (0.00406) | (0.00705) | (0.00585) |
| Constant | 2.09436*** | 0.07248*** | -1.91187*** | 2.21504*** | 0.04893*** | 0.13567*** | 0.11803*** |
|  | (0.07678) | (0.00888) | (0.55208) | (0.59570) | (0.01346) | (0.01669) | (0.01509) |
|  |  |  |  |  |  |  |  |
| Observations | 3,286 | 3,286 | 3,286 | 3,286 | 3,286 | 3,286 | 3,286 |
| No. of practices | 1,248 | 1,248 | 1,248 | 1,248 | 1,248 | 1,248 | 1,248 |

Note: The columns contain coefficients from within-between regressions estimated by maximum likelihood. Each of the seven quality indicators (calculated over the first six months of each year t=2020 to 2023) is regressed on financial motivation and patient-oriented altruism from each year t-1 (2019 to 2022), time dummies (reference year 2023) and a set of controls related to patient, practice, and area characteristics (see Appendix C for details). The regressions are based on an unbalanced sample of 3286 observations from 1248 general practices in Denmark. Observations are weighted according to the share of GPs in the practice responding to the motivation survey in that year. The number of observations per wave is 755 (in 2020), 842 (in 2021), 841 (in 2022), and 848 (in 2023). Standard errors clustered by practice in parentheses.

*p<0.10	**p<0.05	*** p<0.01

# **Appendix G – Regression results with practices weighted by the inverse probability of responding to the survey**

Table G1. Regression results with practices weighted by the inverse probability of responding to the survey

|  | (1) | (2) | (3) | (4) | (5) | (6) | (7) |
| --- | --- | --- | --- | --- | --- | --- | --- |
|  | Average number of prescriptions (over 65) | Share of polypharmacy patients  (over 65) | Average DDDs, NSAIDs without PPI (over 65) | Average DDDs, sleeping medication (18+) | Share of patients with preventive home visit (75+) | Share of patients with annual control for chronic disease (18+) | Share of COPD and asthma patients with spirometry test (18+) |
|  |  |  |  |  |  |  |  |
| Lagged financial motivation (within) | -0.00255  (0.01804) | 0.00114  (0.00333) | 0.02622  (0.16235) | -0.36136***  (0.13418) | 0.00186  (0.00446) | 0.01320***  (0.00478) | -0.00287  (0.00548) |
|  |  |  |  |  |  |  |  |
| Lagged patient-oriented altruism (within) | 0.02140  (0.02500) | -0.00243  (0.00390) | -0.06628  (0.23711) | 0.09830  (0.17828) | 0.00415  (0.00793) | 0.01361*  (0.00783) | 0.00252  (0.00774) |
|  |  |  |  |  |  |  |  |
| Lagged financial motivation (between) | -0.02865  (0.03478) | -0.00418  (0.00402) | 0.51378**  (0.25250) | 0.30867  (0.27265) | 0.02630***  (0.00775) | 0.02526***  (0.00727) | 0.00492  (0.00774) |
|  |  |  |  |  |  |  |  |
| Lagged patient-oriented altruism (between) | 0.04873  (0.06266) | 0.00797  (0.00696) | 1.80656***  (0.42600) | 0.69679*  (0.42243) | -0.01793  (0.01150) | -0.03712***  (0.01293) | -0.02020*  (0.01190) |
|  |  |  |  |  |  |  |  |
| List per GP (within) | 0.00001 | -0.00000 | 0.00000 | 0.00007 | -0.00001 | -0.00001 | -0.00000 |
|  | (0.00002) | (0.00000) | (0.00010) | (0.00009) | (0.00001) | (0.00000) | (0.00000) |
| List per GP (between) | 0.00001 | 0.00000 | 0.00037*** | -0.00017 | 0.00000 | 0.00000 | 0.00000 |
|  | (0.00001) | (0.00000) | (0.00011) | (0.00012) | (0.00000) | (0.00000) | (0.00000) |
| SOCIO1 (within) | 2.57065* | 0.41437* | -1.60433 | -3.70080 | 0.85953* | 0.11158 | 0.67814* |
|  | (1.36250) | (0.23182) | (18.08656) | (9.53537) | (0.52208) | (0.26245) | (0.39255) |
| SOCIO2 (within) | 1.62383* | 0.24262* | -10.68910 | -6.03980 | 0.49617* | -0.10727 | -0.00107 |
|  | (0.95046) | (0.13317) | (8.83587) | (6.02237) | (0.28823) | (0.16342) | (0.24337) |
| SOCIO3 (within) | -2.74361** | -0.36247** | 7.70109 | 6.90628 | -0.56302* | -0.44895* | -0.06290 |
|  | (1.26391) | (0.17820) | (9.88739) | (9.00487) | (0.30548) | (0.26753) | (0.26946) |
| SOCIO4 (within) | -2.69892 | -0.57750 | -29.02539 | -8.84484 | -0.81225 | -0.18834 | -0.97910 |
|  | (2.81812) | (0.42224) | (18.03903) | (17.05869) | (0.64794) | (0.42684) | (0.75987) |
| SOCIO5 (within) | -0.03170 | 0.05110 | 6.36751 | 2.59755 | 0.00320 | -0.07013 | -0.00353 |
|  | (0.58533) | (0.11952) | (5.84186) | (5.64339) | (0.19918) | (0.14707) | (0.16356) |
| SOCIO6 (within) | -0.13747 | -0.08341 | 18.45488*** | 6.87800 | -0.05850 | 0.10746 | -0.13789 |
|  | (0.60942) | (0.09674) | (6.82107) | (6.14762) | (0.23284) | (0.13751) | (0.18798) |
| SOCIO7 (within) | 2.52393*** | 0.29628** | -12.06006* | 22.70753*** | 0.29752 | 0.24078 | 0.21445 |
|  | (0.79264) | (0.12400) | (7.05830) | (8.10633) | (0.29214) | (0.21281) | (0.25814) |
| CCI = 1 (within) | 2.68073* | 0.20137 | -13.61197 | -16.10812 | -0.07933 | 0.83323*** | 0.52138 |
|  | (1.42076) | (0.23010) | (11.56479) | (11.38859) | (0.32086) | (0.27197) | (0.35197) |
| CCI > 1 (within) | 2.80926** | 0.44700** | -29.78258*** | 9.12602 | 0.92581** | 0.13523 | 1.18647*** |
|  | (1.10806) | (0.18538) | (10.80244) | (11.46204) | (0.42929) | (0.23169) | (0.32125) |
| SOCIO1 (between) | -1.77828 | 0.40930 | 3.33028 | -68.14365** | 0.27233 | -0.99757* | 0.33346 |
|  | (3.12225) | (0.35168) | (21.04114) | (29.21400) | (0.54369) | (0.58497) | (0.53231) |
| SOCIO2 (between) | 3.12399*** | 0.31289*** | 15.17272*** | -14.32954*** | 0.14796 | -0.10263 | -0.01455 |
|  | (0.62266) | (0.07240) | (4.09024) | (4.02705) | (0.09780) | (0.12439) | (0.11246) |
| SOCIO3 (between) | 2.44364*** | 0.17871** | -8.92752* | 66.56220*** | -0.12930 | 0.19761 | -0.25112** |
|  | (0.59302) | (0.08391) | (4.91260) | (13.14795) | (0.09757) | (0.12219) | (0.12074) |
| SOCIO4 (between) | 0.57375 | 0.26961 | 32.37279** | -25.51975 | -0.50117 | 0.16144 | 0.09117 |
|  | (2.03360) | (0.22983) | (14.48665) | (23.08926) | (0.32492) | (0.44102) | (0.43338) |
| SOCIO5 (between) | 0.35649** | 0.03169 | -0.83553 | -3.79958*** | 0.05555 | -0.00717 | 0.00017 |
|  | (0.17937) | (0.02015) | (1.17307) | (1.46458) | (0.03745) | (0.03613) | (0.03364) |
| SOCIO6 (between) | -2.18983*** | -0.22764*** | 0.15632 | 8.23420*** | -0.00424 | -0.30200*** | -0.02371 |
|  | (0.35778) | (0.04286) | (2.44983) | (2.90695) | (0.05693) | (0.07504) | (0.07213) |
| SOCIO7 (between) | 3.74969*** | 0.52648*** | 19.98639*** | -12.44352*** | -0.12373 | 0.16469 | 0.26547** |
|  | (0.59015) | (0.07533) | (4.61504) | (4.75584) | (0.09843) | (0.14964) | (0.12659) |
| CCI = 1 (between) | 10.51614*** | 1.13664*** | -7.77510 | 0.66065 | -0.11913 | 1.00082*** | 1.19140*** |
|  | (1.17454) | (0.14700) | (7.63120) | (10.79327) | (0.17341) | (0.27563) | (0.22451) |
| CCI > 1 (between) | 3.31852*** | 0.20472 | 6.83592 | 15.15453* | 0.12331 | 0.85263*** | -0.22948 |
|  | (0.99299) | (0.13350) | (6.57184) | (9.18997) | (0.15620) | (0.23115) | (0.20425) |
| 2020 (within) | -0.28405*** | -0.02968*** | 0.65746*** | -0.97285*** | -0.00345 | -0.03594*** | -0.02881*** |
|  | (0.01603) | (0.00247) | (0.17217) | (0.11334) | (0.00585) | (0.00294) | (0.00468) |
| 2021 (within) | -0.16374*** | -0.01666*** | 0.32683*** | -0.52688*** | 0.00267 | -0.02437*** | -0.03086*** |
|  | (0.01020) | (0.00166) | (0.09700) | (0.07344) | (0.00348) | (0.00198) | (0.00306) |
| 2022 (within) | -0.11150*** | -0.01016*** | 0.15433** | -0.28644*** | 0.00328 | -0.01630*** | 0.00178 |
|  | (0.00759) | (0.00123) | (0.06453) | (0.05332) | (0.00271) | (0.00137) | (0.00202) |
| 2020 (between) | -0.29676*** | -0.03774*** | 0.77307** | -0.51754 | -0.00744 | -0.04112*** | -0.04165*** |
|  | (0.04128) | (0.00487) | (0.30029) | (0.34679) | (0.00701) | (0.00873) | (0.00782) |
| 2021 (between) | -0.22505*** | -0.02660*** | 0.42453* | -0.73011*** | 0.00043 | -0.02538*** | -0.03248*** |
|  | (0.03738) | (0.00455) | (0.23621) | (0.24161) | (0.00640) | (0.00793) | (0.00784) |
| 2022 (between) | -0.12512*** | -0.01396*** | 0.27654 | -0.42753* | 0.00073 | -0.01703** | -0.00383 |
|  | (0.03629) | (0.00444) | (0.23679) | (0.25290) | (0.00647) | (0.00766) | (0.00739) |
| Region North | 0.32293*** | 0.03070*** | -0.63527*** | -2.15525*** | -0.01901*** | 0.05199*** | 0.03129*** |
|  | (0.03330) | (0.00407) | (0.21744) | (0.34712) | (0.00479) | (0.00711) | (0.00679) |
| Region Central | 0.31130*** | 0.03229*** | -0.87565*** | -1.35642*** | -0.00044 | 0.03829*** | 0.03289*** |
|  | (0.02633) | (0.00335) | (0.16735) | (0.37440) | (0.00445) | (0.00540) | (0.00556) |
| Region South | 0.09737*** | 0.00767*** | -0.76639*** | -2.49218*** | -0.01482*** | 0.02715*** | 0.02585*** |
|  | (0.02397) | (0.00291) | (0.17019) | (0.30354) | (0.00441) | (0.00562) | (0.00571) |
| Region Zealand | 0.09836*** | 0.01086*** | 0.37389** | -0.68188*** | -0.01936*** | 0.01575** | -0.00241 |
|  | (0.02372) | (0.00278) | (0.18914) | (0.25251) | (0.00407) | (0.00637) | (0.00529) |
| Rural area | -0.05299*** | -0.00521** | 0.12425 | 0.10003 | -0.00408 | -0.01022** | -0.00486 |
|  | (0.01691) | (0.00204) | (0.11609) | (0.11827) | (0.00251) | (0.00406) | (0.00393) |
| Urban area | 0.01521 | 0.00150 | 0.19130 | 0.54213*** | 0.00080 | -0.00801* | -0.01355*** |
|  | (0.02011) | (0.00225) | (0.12969) | (0.14558) | (0.00344) | (0.00441) | (0.00412) |
| Risk of GP shortage | -0.07530*** | -0.00894*** | 0.06472 | -0.67173*** | -0.00565 | 0.00943 | -0.00005 |
|  | (0.02749) | (0.00304) | (0.20871) | (0.22443) | (0.00395) | (0.00700) | (0.00562) |
| Constant | 2.09993*** | 0.07359*** | -1.84503*** | 2.25326*** | 0.04917*** | 0.13546*** | 0.11781*** |
|  | (0.07691) | (0.00898) | (0.54775) | (0.58764) | (0.01373) | (0.01662) | (0.01476) |
|  |  |  |  |  |  |  |  |
| Observations | 3,286 | 3,286 | 3,286 | 3,286 | 3,286 | 3,286 | 3,286 |
| No. of practices | 1,248 | 1,248 | 1,248 | 1,248 | 1,248 | 1,248 | 1,248 |

Note: The columns contain coefficients from within-between regressions estimated by maximum likelihood. Each of the seven quality indicators (calculated over the first six months of each year t=2020 to 2023) is regressed on financial motivation and patient-oriented altruism from each year t-1 (2019 to 2022), time dummies (reference year 2023) and a set of controls related to patient, practice, and area characteristics (see Appendix C for details). The regressions are based on an unbalanced sample of 3286 observations from 1248 general practices in Denmark. Observations are weighted according to i) the share of GPs in the practice responding to the motivation survey in that year and ii) the inverse probability of responding to the survey. Sampling weights are estimated separately for each wave using a logistic regression of survey participation on observable patient, practice, and area characteristics (listed in Appendix C). The number of observations per wave is 755 (in 2020), 842 (in 2021), 841 (in 2022), and 848 (in 2023). Standard errors clustered by practice in parentheses.

*p<0.10	**p<0.05	*** p<0.01

# **Appendix H – Regression results on a balanced panel of general practices**

Table H1. Regression results on a balanced panel of general practices

|  | (1) | (2) | (3) | (4) | (5) | (6) | (7) |
| --- | --- | --- | --- | --- | --- | --- | --- |
|  | Average number of prescriptions (over 65) | Share of polypharmacy patients  (over 65) | Average DDDs, NSAIDs without PPI (over 65) | Average DDDs, sleeping medication (18+) | Share of patients with preventive home visit (75+) | Share of patients with annual control for chronic disease (18+) | Share of COPD and asthma patients with spirometry test (18+) |
|  |  |  |  |  |  |  |  |
| Lagged financial motivation (within) | -0.02290  (0.01707) | -0.00086  (0.00309) | -0.15001  (0.14671) | -0.14480  (0.15428) | 0.00622  (0.00474) | 0.01555***  (0.00562) | -0.00256  (0.00719) |
|  |  |  |  |  |  |  |  |
| Lagged patient-oriented altruism (within) | 0.02742  (0.02583) | -0.00175  (0.00430) | -0.06103  (0.18063) | 0.16708  (0.21229) | 0.00111  (0.00764) | 0.00577  (0.00759) | 0.01179  (0.00975) |
|  |  |  |  |  |  |  |  |
| Lagged financial motivation (between) | -0.03023  (0.06527) | -0.00447  (0.00722) | 0.30329  (0.37825) | -0.22024  (0.36310) | 0.02190*  (0.01214) | 0.03753***  (0.01253) | 0.00511  (0.01341) |
|  |  |  |  |  |  |  |  |
| Lagged patient-oriented altruism (between) | 0.27876***  (0.10204) | 0.03375***  (0.01123) | 2.17935***  (0.76107) | 1.48315**  (0.64198) | -0.03057*  (0.01661) | -0.02937  (0.01925) | -0.00652  (0.02337) |
|  |  |  |  |  |  |  |  |
| List per GP (within) | -0.00001 | -0.00000 | -0.00001 | 0.00003 | -0.00001*** | -0.00001 | -0.00000 |
|  | (0.00002) | (0.00000) | (0.00013) | (0.00013) | (0.00000) | (0.00000) | (0.00001) |
| List per GP (between) | 0.00010*** | 0.00001*** | 0.00064*** | 0.00016 | 0.00001 | 0.00000 | 0.00000 |
|  | (0.00003) | (0.00000) | (0.00020) | (0.00017) | (0.00001) | (0.00001) | (0.00001) |
| SOCIO1 (within) | 3.00863 | 0.12871 | -27.92011 | -12.69949 | 0.19732 | -0.11294 | 1.04500** |
|  | (1.83339) | (0.30246) | (20.67376) | (10.67094) | (0.54790) | (0.30385) | (0.51071) |
| SOCIO2 (within) | 0.28824 | 0.21631 | -19.85566** | -9.11404 | -0.06122 | -0.43493* | 0.34752 |
|  | (1.04661) | (0.16919) | (8.63832) | (8.24291) | (0.34202) | (0.24239) | (0.36807) |
| SOCIO3 (within) | -1.47027 | -0.26739 | 14.29323 | 9.13321 | -0.15458 | -0.60330* | -0.53905 |
|  | (1.25295) | (0.19907) | (9.73670) | (11.28880) | (0.34662) | (0.32084) | (0.37581) |
| SOCIO4 (within) | 0.61420 | -0.57823 | -16.70405 | 4.32511 | 0.05527 | 0.65080 | -0.30470 |
|  | (3.04058) | (0.41452) | (18.24562) | (18.99301) | (0.69472) | (0.51826) | (1.06643) |
| SOCIO5 (within) | 1.26102 | 0.19266 | 4.13227 | -1.75599 | -0.19205 | 0.12706 | -0.22026 |
|  | (0.86577) | (0.12498) | (4.41682) | (6.26157) | (0.16193) | (0.20828) | (0.22642) |
| SOCIO6 (within) | 0.24819 | -0.06249 | 15.66199** | -3.82603 | 0.06349 | 0.29987* | -0.13411 |
|  | (0.73686) | (0.11783) | (7.56380) | (6.14907) | (0.22663) | (0.16493) | (0.23976) |
| SOCIO7 (within) | 3.18129*** | 0.50025*** | -5.30313 | 15.23589 | -0.00663 | 0.59937** | 0.01400 |
|  | (0.94639) | (0.14289) | (8.09283) | (9.59285) | (0.29955) | (0.27047) | (0.34574) |
| CCI = 1 (within) | -0.03291 | -0.01763 | -10.93007 | -15.07470 | -0.44106 | 0.66545* | 0.39511 |
|  | (1.40652) | (0.21036) | (13.97349) | (13.62867) | (0.42846) | (0.37225) | (0.59278) |
| CCI > 1 (within) | 2.45245* | 0.46073** | -26.55946** | 10.00146 | -0.16496 | 0.43012 | 1.15719** |
|  | (1.32625) | (0.19652) | (12.74848) | (8.57962) | (0.32511) | (0.27394) | (0.47800) |
| SOCIO1 (between) | -4.30070 | 0.04985 | -10.84764 | 34.55639 | 1.74966 | -1.37449 | -1.08998 |
|  | (6.69796) | (0.77517) | (42.10748) | (33.82655) | (1.24390) | (0.97178) | (1.09302) |
| SOCIO2 (between) | 2.25186* | 0.21784* | 3.56389 | -21.57561*** | 0.24945 | -0.17343 | -0.04466 |
|  | (1.19574) | (0.13102) | (6.11009) | (5.75665) | (0.16745) | (0.17949) | (0.19576) |
| SOCIO3 (between) | 3.50993*** | 0.41517*** | 19.13015** | 37.32269*** | -0.63319*** | -0.22993 | -0.56357** |
|  | (1.27438) | (0.14394) | (8.49597) | (8.02040) | (0.19098) | (0.24724) | (0.25119) |
| SOCIO4 (between) | 1.55857 | 0.20039 | 9.85714 | 21.70215 | -0.27059 | 1.93270*** | 0.66174 |
|  | (3.58763) | (0.40318) | (26.71246) | (24.44023) | (0.67670) | (0.65349) | (0.75259) |
| SOCIO5 (between) | -0.06112 | -0.02081 | -3.69782** | -5.89425*** | 0.02228 | -0.00870 | 0.09042* |
|  | (0.26666) | (0.03043) | (1.88287) | (2.05429) | (0.05031) | (0.05687) | (0.05190) |
| SOCIO6 (between) | -1.84984*** | -0.20018*** | 3.11094 | 8.84122** | 0.15652 | -0.12951 | 0.05559 |
|  | (0.57672) | (0.06355) | (3.64533) | (3.84927) | (0.09866) | (0.10625) | (0.11536) |
| SOCIO7 (between) | 3.13514*** | 0.42909*** | 4.94280 | -29.58494*** | -0.13660 | 0.35529* | 0.18451 |
|  | (1.05330) | (0.11891) | (7.49352) | (5.94075) | (0.15842) | (0.18327) | (0.20208) |
| CCI = 1 (between) | 11.02786*** | 1.12311*** | -19.03732 | 22.16216* | -0.06960 | 1.11003*** | 1.40718*** |
|  | (1.89497) | (0.21590) | (11.72654) | (12.57597) | (0.33902) | (0.38331) | (0.37324) |
| CCI > 1 (between) | 1.58641 | 0.05438 | -1.08612 | 35.31836*** | 0.03818 | 0.90650*** | -0.21265 |
|  | (1.77076) | (0.20261) | (10.39062) | (12.91716) | (0.31282) | (0.31345) | (0.32996) |
| Region North | 0.28948*** | 0.02407*** | -1.19786*** | -1.40161*** | -0.01362 | 0.04621*** | 0.04943*** |
|  | (0.06087) | (0.00697) | (0.31829) | (0.31690) | (0.00850) | (0.01164) | (0.01214) |
| Region Central | 0.25088*** | 0.02325*** | -1.19626*** | -0.08466 | 0.01004 | 0.04558*** | 0.03878*** |
|  | (0.04494) | (0.00500) | (0.26719) | (0.28846) | (0.00737) | (0.00905) | (0.00877) |
| Region South | 0.10111** | 0.00599 | -0.98486*** | -1.62436*** | -0.00184 | 0.02662*** | 0.02853*** |
|  | (0.04038) | (0.00428) | (0.28242) | (0.28068) | (0.00668) | (0.00867) | (0.00880) |
| Region Zealand | 0.04727 | 0.00246 | 0.38905 | -0.44589 | -0.00936 | 0.01872** | -0.00067 |
|  | (0.03942) | (0.00424) | (0.27859) | (0.27445) | (0.00701) | (0.00814) | (0.00827) |
| Rural area | -0.03673 | -0.00258 | 0.10371 | 0.00966 | -0.00147 | -0.01117** | -0.00248 |
|  | (0.02410) | (0.00265) | (0.15279) | (0.14647) | (0.00357) | (0.00474) | (0.00485) |
| Urban area | 0.02687 | 0.00283 | 0.02091 | 0.13826 | -0.00245 | -0.00574 | -0.01372** |
|  | (0.03037) | (0.00338) | (0.19212) | (0.21299) | (0.00505) | (0.00630) | (0.00693) |
| Risk of GP shortage | -0.09736** | -0.01146** | -0.05512 | -0.31163 | -0.00127 | 0.01215 | -0.00073 |
|  | (0.04683) | (0.00497) | (0.34762) | (0.27665) | (0.00652) | (0.00978) | (0.00800) |
| Year 2021 | 0.11812*** | 0.01135*** | -0.46061*** | 0.40725*** | 0.00578* | 0.01120*** | 0.00171 |
|  | (0.01047) | (0.00163) | (0.11825) | (0.07044) | (0.00318) | (0.00190) | (0.00340) |
| Year 2022 | 0.18230*** | 0.01787*** | -0.72688*** | 0.66764*** | 0.00452 | 0.01684*** | 0.03861*** |
|  | (0.01652) | (0.00268) | (0.20088) | (0.12110) | (0.00481) | (0.00321) | (0.00558) |
| Year 2023 | 0.28021*** | 0.02747*** | -0.81374*** | 1.03178*** | 0.00487 | 0.03107*** | 0.04094*** |
|  | (0.01809) | (0.00289) | (0.19513) | (0.14504) | (0.00511) | (0.00373) | (0.00617) |
| Constant | 1.60966*** | 0.01896 | -0.43233 | -0.13935 | 0.02702 | 0.06572** | 0.06640** |
|  | (0.12109) | (0.01348) | (0.83713) | (0.89115) | (0.02232) | (0.02840) | (0.02688) |
|  |  |  |  |  |  |  |  |
| Observations | 1,668 | 1,668 | 1,668 | 1,668 | 1,668 | 1,668 | 1,668 |
| No. of practices | 417 | 417 | 417 | 417 | 417 | 417 | 417 |

Note: The columns contain coefficients from within-between regressions estimated by maximum likelihood (MLE). Each of the seven quality indicators (calculated over the first six months of each year t=2020 to 2023) is regressed on financial motivation and patient-oriented altruism from each year t-1 (2019 to 2022), time dummies (reference year 2020) and a set of controls related to patient, practice, and area characteristics (see Appendix C for details). Regressions are based on a balanced sample of 1668 observations from 417 general practices in Denmark. Observations are weighted according to the share of GPs in the practice responding to the motivation survey in that year. Standard errors clustered at the practice level in parentheses.

*p<0.10	**p<0.05	*** p<0.01

# **Appendix I – Regression results for the subset of practices with 100% survey response rate**

Table I1. Regression results for the subset of practices with 100% survey response rate

|  | (1) | (2) | (3) | (4) | (5) | (6) | (7) |
| --- | --- | --- | --- | --- | --- | --- | --- |
|  | Average number of prescriptions (over 65) | Share of polypharmacy patients  (over 65) | Average DDDs, NSAIDs without PPI (over 65) | Average DDDs, sleeping medication (18+) | Share of patients with preventive home visit (75+) | Share of patients with annual control for chronic disease (18+) | Share of COPD and asthma patients with spirometry test (18+) |
|  |  |  |  |  |  |  |  |
| Lagged financial motivation (within) | -0.02591  (0.02746) | -0.00044  (0.00481) | -0.00558  (0.23451) | -0.41198*  (0.22055) | 0.00932  (0.00640) | 0.01881***  (0.00700) | -0.00093  (0.00839) |
|  |  |  |  |  |  |  |  |
| Lagged patient-oriented altruism (within) | 0.03606  (0.03579) | -0.00556  (0.00561) | -0.04318  (0.30967) | -0.16789  (0.25201) | 0.00860  (0.01118) | 0.00298  (0.01039) | 0.00518  (0.01155) |
|  |  |  |  |  |  |  |  |
| Lagged financial motivation (between) | -0.06344  (0.04935) | -0.00923  (0.00577) | 0.87941**  (0.36241) | 0.29390  (0.36963) | 0.02657**  (0.01046) | 0.02350**  (0.01012) | -0.00043  (0.01075) |
|  |  |  |  |  |  |  |  |
| Lagged patient-oriented altruism (between) | 0.00775  (0.09564) | 0.00057  (0.01040) | 2.45486***  (0.62864) | 0.93427  (0.61899) | -0.02629*  (0.01484) | -0.05965***  (0.01970) | -0.01895  (0.01771) |
|  |  |  |  |  |  |  |  |
| List per GP (within) | 0.00001 | 0.00000 | 0.00008 | 0.00002 | -0.00001 | -0.00001 | -0.00000 |
|  | (0.00003) | (0.00000) | (0.00017) | (0.00018) | (0.00001) | (0.00001) | (0.00001) |
| List per GP (between) | 0.00002 | 0.00000 | 0.00017 | -0.00008 | 0.00001* | 0.00001 | 0.00000 |
|  | (0.00002) | (0.00000) | (0.00013) | (0.00014) | (0.00000) | (0.00000) | (0.00000) |
| SOCIO1 (within) | 1.16499 | 0.27837 | -7.19009 | -4.29971 | 0.85635 | -0.01118 | 0.12191 |
|  | (1.94185) | (0.29643) | (19.63642) | (12.58835) | (0.58828) | (0.31529) | (0.53354) |
| SOCIO2 (within) | 2.27248** | 0.33267** | -9.06493 | -6.75988 | 0.67163* | -0.21210 | -0.10132 |
|  | (1.04859) | (0.15797) | (10.75375) | (8.10585) | (0.35629) | (0.19058) | (0.33734) |
| SOCIO3 (within) | -2.76430* | -0.42311* | 6.98802 | 6.01508 | -0.56007 | -0.43108 | -0.12595 |
|  | (1.58804) | (0.21779) | (12.32553) | (12.06468) | (0.35236) | (0.28924) | (0.37320) |
| SOCIO4 (within) | -2.54183 | -0.65101 | -42.78336* | -28.90261 | -0.10851 | -0.00620 | -0.66527 |
|  | (3.40365) | (0.47823) | (22.46827) | (22.29955) | (0.83911) | (0.55859) | (0.97590) |
| SOCIO5 (within) | 0.22423 | 0.09436 | 12.79034 | 2.76098 | 0.10115 | -0.09212 | -0.01825 |
|  | (0.75776) | (0.13302) | (9.10824) | (5.99401) | (0.27256) | (0.13927) | (0.20743) |
| SOCIO6 (within) | -0.04595 | -0.17468 | 24.20704*** | 7.38621 | 0.10753 | 0.00893 | -0.09612 |
|  | (0.79616) | (0.12405) | (8.54463) | (7.91168) | (0.23485) | (0.16465) | (0.25987) |
| SOCIO7 (within) | 2.94322*** | 0.38467** | -14.67922 | 28.51247*** | 0.23940 | 0.22380 | 0.15253 |
|  | (1.10177) | (0.15550) | (9.12382) | (10.60547) | (0.28130) | (0.25230) | (0.35801) |
| CCI = 1 (within) | 3.39834** | 0.35252 | -8.62495 | -18.75900 | 0.04840 | 0.68427** | 0.45597 |
|  | (1.66573) | (0.25658) | (12.73997) | (14.39617) | (0.35156) | (0.30779) | (0.47162) |
| CCI > 1 (within) | 3.65363*** | 0.56163*** | -18.75012 | 9.10633 | 0.62061* | 0.15816 | 1.12368** |
|  | (1.29969) | (0.20525) | (11.84311) | (13.48532) | (0.36650) | (0.27360) | (0.44188) |
| SOCIO1 (between) | 1.90956 | 0.50083 | 7.33392 | 8.54743 | 0.62884 | -0.99002 | 1.05365 |
|  | (4.50529) | (0.49869) | (28.25276) | (25.03172) | (0.74958) | (0.80235) | (0.73132) |
| SOCIO2 (between) | 2.59449*** | 0.24637*** | 13.12951** | -13.55693*** | 0.06720 | -0.02868 | -0.06919 |
|  | (0.82170) | (0.09416) | (5.36843) | (5.05238) | (0.11127) | (0.16203) | (0.14534) |
| SOCIO3 (between) | 3.04324*** | 0.34402*** | -1.93529 | 25.92757*** | -0.24948* | 0.21211 | -0.31845 |
|  | (1.04522) | (0.12229) | (6.87598) | (7.42000) | (0.14343) | (0.22328) | (0.21333) |
| SOCIO4 (between) | 0.74721 | 0.19621 | 31.69666* | 46.14721** | -0.35224 | -0.01378 | 0.18025 |
|  | (2.81881) | (0.30733) | (17.70171) | (18.19988) | (0.40720) | (0.57545) | (0.59092) |
| SOCIO5 (between) | 0.16265 | 0.01393 | -2.33100 | -4.45982*** | 0.08283 | -0.01055 | 0.00793 |
|  | (0.24465) | (0.02785) | (1.64589) | (1.62072) | (0.05091) | (0.04956) | (0.04644) |
| SOCIO6 (between) | -2.21589*** | -0.22684*** | 0.98263 | 4.55024 | 0.06620 | -0.31061*** | 0.00691 |
|  | (0.47968) | (0.05766) | (3.31526) | (3.34304) | (0.06807) | (0.10037) | (0.09372) |
| SOCIO7 (between) | 4.44967*** | 0.60432*** | 21.21139*** | -8.48080 | -0.22084* | 0.16754 | 0.16368 |
|  | (0.80235) | (0.10215) | (6.47796) | (5.75110) | (0.12307) | (0.19827) | (0.17097) |
| CCI = 1 (between) | 11.15376*** | 1.14922*** | -6.73650 | 33.24214*** | 0.10544 | 0.87653** | 0.96768*** |
|  | (1.60225) | (0.19686) | (9.93619) | (11.25547) | (0.22216) | (0.36125) | (0.29209) |
| CCI > 1 (between) | 2.26346* | 0.05760 | -0.26660 | 23.50421** | 0.06950 | 0.75717** | -0.11595 |
|  | (1.22408) | (0.16712) | (8.50433) | (10.34485) | (0.18876) | (0.29622) | (0.25876) |
| 2020 (within) | -0.26359*** | -0.02869*** | 0.75668*** | -0.93279*** | -0.00632 | -0.03398*** | -0.01877** |
|  | (0.02169) | (0.00326) | (0.21715) | (0.17594) | (0.00704) | (0.00415) | (0.00739) |
| 2021 (within) | -0.15497*** | -0.01618*** | 0.40312*** | -0.49316*** | -0.00281 | -0.02333*** | -0.02263*** |
|  | (0.01400) | (0.00222) | (0.12850) | (0.11275) | (0.00414) | (0.00274) | (0.00485) |
| 2022 (within) | -0.11124*** | -0.00942*** | 0.21301** | -0.26615*** | 0.00051 | -0.01492*** | 0.00417 |
|  | (0.01044) | (0.00159) | (0.09114) | (0.08178) | (0.00294) | (0.00194) | (0.00291) |
| 2020 (between) | -0.33592*** | -0.04052*** | 1.05368** | -1.23141*** | -0.00844 | -0.04823*** | -0.05118*** |
|  | (0.06119) | (0.00718) | (0.42869) | (0.39375) | (0.00931) | (0.01239) | (0.01109) |
| 2021 (between) | -0.27133*** | -0.03050*** | 1.06881*** | -0.97312*** | 0.00020 | -0.02832** | -0.03313*** |
|  | (0.05514) | (0.00675) | (0.33286) | (0.31196) | (0.00815) | (0.01151) | (0.01116) |
| 2022 (between) | -0.17595*** | -0.01922*** | 0.63707* | -0.43630 | 0.00674 | -0.02033* | -0.00701 |
|  | (0.05345) | (0.00661) | (0.34800) | (0.36914) | (0.00854) | (0.01134) | (0.01080) |
| Region North | 0.28179*** | 0.02313*** | -0.91465*** | -1.40219*** | -0.00852 | 0.06405*** | 0.03853*** |
|  | (0.05252) | (0.00615) | (0.32893) | (0.32294) | (0.00698) | (0.01083) | (0.01110) |
| Region Central | 0.28191*** | 0.02544*** | -1.25367*** | -0.54661* | 0.01502** | 0.04238*** | 0.03362*** |
|  | (0.04402) | (0.00512) | (0.26121) | (0.28760) | (0.00706) | (0.00879) | (0.00922) |
| Region South | 0.07929** | 0.00344 | -0.98900*** | -1.93416*** | -0.00403 | 0.03445*** | 0.03286*** |
|  | (0.03760) | (0.00435) | (0.27364) | (0.27679) | (0.00707) | (0.00916) | (0.00969) |
| Region Zealand | 0.09858*** | 0.00822* | 0.05527 | -0.13610 | -0.01146** | 0.02952*** | -0.00250 |
|  | (0.03637) | (0.00433) | (0.27343) | (0.30007) | (0.00575) | (0.01003) | (0.00865) |
| Rural area | -0.08089*** | -0.00905*** | 0.26122 | 0.06599 | -0.00348 | -0.01584** | -0.00020 |
|  | (0.02789) | (0.00346) | (0.19337) | (0.18968) | (0.00389) | (0.00663) | (0.00653) |
| Urban area | -0.00761 | -0.00059 | 0.23790 | 0.42302** | -0.00039 | -0.00642 | -0.01392** |
|  | (0.02814) | (0.00321) | (0.19921) | (0.21401) | (0.00503) | (0.00652) | (0.00616) |
| Risk of GP shortage | -0.08861** | -0.01112** | 0.11506 | -0.74002** | -0.00115 | 0.01385 | 0.00125 |
|  | (0.04084) | (0.00441) | (0.31774) | (0.29353) | (0.00549) | (0.01017) | (0.00837) |
| Constant | 2.20307*** | 0.09252*** | -2.12041*** | 1.69426** | 0.03379* | 0.14617*** | 0.12036*** |
|  | (0.11370) | (0.01290) | (0.79049) | (0.79903) | (0.01787) | (0.02289) | (0.02096) |
|  |  |  |  |  |  |  |  |
| Observations | 1,375 | 1,375 | 1,375 | 1,375 | 1,375 | 1,375 | 1,375 |
| No. of practices | 679 | 679 | 679 | 679 | 679 | 679 | 679 |

Note: The columns contain coefficients from within-between regressions estimated by maximum likelihood (MLE). Each of the seven quality indicators (calculated over the first six months of each year t=2020 to 2023) is regressed on financial motivation and patient-oriented altruism from each year t-1 (2019 to 2022), time dummies (reference year 2023) and a set of controls related to patient, practice, and area characteristics (see Appendix C for details). Regressions are based on the subset of general practices with a 100% response rate to the motivation survey (1375 observations from 679 general practices in Denmark, which includes solo practices operated by a single GP). The number of observations per wave is 308 (2020), 352 (2021), 360 (2022), and 355 (2023). Standard errors clustered at the practice level in parentheses.

*p<0.10	**p<0.05	*** p<0.01

# **Appendix J – Regression results with motivation sum scores weighted by CFA factor loadings**

Table J1. Regression results with motivation sum scores weighted by CFA factor loadings

|  | (1) | (2) | (3) | (4) | (5) | (6) | (7) |
| --- | --- | --- | --- | --- | --- | --- | --- |
|  | Average number of prescriptions (over 65) | Share of polypharmacy patients  (over 65) | Average DDDs, NSAIDs without PPI (over 65) | Average DDDs, sleeping medication (18+) | Share of patients with preventive home visit (75+) | Share of patients with annual control for chronic disease (18+) | Share of COPD and asthma patients with spirometry test (18+) |
|  |  |  |  |  |  |  |  |
| Lagged financial motivation (within) | -0.01145  (0.01453) | -0.00220  (0.00237) | 0.12831  (0.09806) | -0.09247  (0.08977) | 0.00568*  (0.00332) | 0.00286  (0.00254) | -0.00071  (0.00463) |
|  |  |  |  |  |  |  |  |
| Lagged patient-oriented altruism (within) | 0.01774  (0.02208) | -0.00116  (0.00348) | -0.15580  (0.18708) | 0.02980  (0.16638) | 0.00516  (0.00738) | 0.00819  (0.00635) | 0.00545  (0.00793) |
|  |  |  |  |  |  |  |  |
| Lagged financial motivation (between) | -0.03027  (0.03235) | -0.00467  (0.00373) | 0.51774**  (0.21781) | 0.23757  (0.24315) | 0.01099*  (0.00658) | 0.01055  (0.00704) | -0.00697  (0.00728) |
|  |  |  |  |  |  |  |  |
| Lagged patient-oriented altruism (between) | 0.03901  (0.06568) | 0.00754  (0.00734) | 1.80519***  (0.43811) | 0.50758  (0.46086) | -0.01545  (0.01151) | -0.03073**  (0.01365) | -0.01510  (0.01268) |
|  |  |  |  |  |  |  |  |
| List per GP (within) | 0.00001 | 0.00000 | 0.00002 | 0.00008 | -0.00001 | -0.00001** | -0.00000 |
|  | (0.00001) | (0.00000) | (0.00009) | (0.00010) | (0.00001) | (0.00000) | (0.00000) |
| List per GP (between) | 0.00001 | 0.00000 | 0.00039*** | -0.00016 | 0.00000 | 0.00000 | 0.00000 |
|  | (0.00001) | (0.00000) | (0.00011) | (0.00012) | (0.00000) | (0.00000) | (0.00000) |
| SOCIO1 (within) | 2.29969 | 0.34012 | -4.02280 | -10.23547 | 0.61624 | 0.05943 | 0.59185 |
|  | (1.40210) | (0.21573) | (13.73220) | (9.24168) | (0.43655) | (0.22985) | (0.39166) |
| SOCIO2 (within) | 1.33073* | 0.21855* | -7.78121 | -6.55444 | 0.47466* | -0.14385 | 0.00754 |
|  | (0.80079) | (0.12237) | (7.85340) | (6.17345) | (0.27772) | (0.14795) | (0.24943) |
| SOCIO3 (within) | -2.05541* | -0.30790** | 5.32562 | 8.57656 | -0.35593 | -0.33626 | -0.15349 |
|  | (1.10487) | (0.15647) | (8.59439) | (8.71810) | (0.25399) | (0.21331) | (0.28251) |
| SOCIO4 (within) | -1.59345 | -0.40177 | -23.09831 | -15.48634 | -0.29263 | -0.30742 | -0.70238 |
|  | (2.40378) | (0.35973) | (16.66354) | (16.17618) | (0.62676) | (0.41515) | (0.73935) |
| SOCIO5 (within) | 0.36590 | 0.11111 | 10.50304 | 1.41123 | 0.08165 | -0.06277 | 0.03294 |
|  | (0.57991) | (0.10111) | (6.97839) | (4.65294) | (0.22889) | (0.12273) | (0.15437) |
| SOCIO6 (within) | 0.13093 | -0.08818 | 17.78508*** | 5.70657 | 0.00534 | 0.07520 | -0.07379 |
|  | (0.57067) | (0.08884) | (6.01087) | (5.64119) | (0.19326) | (0.12212) | (0.18942) |
| SOCIO7 (within) | 2.69824*** | 0.36416*** | -7.25082 | 18.95462** | 0.08093 | 0.27635 | 0.10371 |
|  | (0.75237) | (0.11031) | (6.08398) | (7.37123) | (0.20492) | (0.17787) | (0.25978) |
| CCI = 1 (within) | 2.79062** | 0.29700 | -10.16600 | -15.57206 | -0.12634 | 0.66192*** | 0.61835* |
|  | (1.27318) | (0.19789) | (9.58733) | (11.33576) | (0.27263) | (0.24271) | (0.36589) |
| CCI > 1 (within) | 2.97085*** | 0.46080*** | -21.77825*** | 7.42279 | 0.48161* | 0.23269 | 1.07584*** |
|  | (0.93632) | (0.14736) | (8.25661) | (9.58456) | (0.26181) | (0.20203) | (0.32730) |
| SOCIO1 (between) | -1.66446 | 0.39146 | 2.27977 | -67.31287** | 0.23820 | -1.15670* | 0.30579 |
|  | (3.13741) | (0.36010) | (21.16351) | (29.87459) | (0.57860) | (0.59248) | (0.54561) |
| SOCIO2 (between) | 3.12976*** | 0.31557*** | 15.05961*** | -14.36864*** | 0.16687* | -0.07371 | -0.00573 |
|  | (0.61829) | (0.07196) | (3.99934) | (4.05987) | (0.09229) | (0.12435) | (0.11259) |
| SOCIO3 (between) | 2.42758*** | 0.17926** | -8.92262* | 65.66678*** | -0.18286* | 0.15620 | -0.28133** |
|  | (0.59425) | (0.08410) | (4.79811) | (13.23448) | (0.09975) | (0.12272) | (0.12244) |
| SOCIO4 (between) | 0.55871 | 0.26222 | 30.93702** | -23.44095 | -0.52805 | 0.12842 | 0.08700 |
|  | (2.03257) | (0.23190) | (14.52676) | (23.39668) | (0.32725) | (0.43915) | (0.42801) |
| SOCIO5 (between) | 0.33299* | 0.02950 | -0.66588 | -4.00356*** | 0.06119 | -0.00104 | 0.00349 |
|  | (0.17949) | (0.02017) | (1.19686) | (1.47610) | (0.03825) | (0.03627) | (0.03264) |
| SOCIO6 (between) | -2.17878*** | -0.22701*** | 0.34329 | 8.65960*** | 0.02572 | -0.28910*** | -0.01437 |
|  | (0.35795) | (0.04264) | (2.44817) | (2.92358) | (0.05703) | (0.07518) | (0.07244) |
| SOCIO7 (between) | 3.81626*** | 0.53367*** | 19.50393*** | -13.06116*** | -0.16006 | 0.14724 | 0.23476* |
|  | (0.59051) | (0.07475) | (4.68596) | (4.74022) | (0.09741) | (0.14837) | (0.12817) |
| CCI = 1 (between) | 10.62677*** | 1.14999*** | -7.91426 | 2.23316 | -0.09267 | 1.00871*** | 1.18968*** |
|  | (1.16960) | (0.14440) | (7.59623) | (10.81338) | (0.17143) | (0.27521) | (0.22564) |
| CCI > 1 (between) | 3.17097*** | 0.18610 | 7.45102 | 15.30220* | 0.10261 | 0.84853*** | -0.21553 |
|  | (0.98271) | (0.12958) | (6.61646) | (9.21808) | (0.15702) | (0.23100) | (0.20346) |
| 2020 (within) | -0.27985*** | -0.02918*** | 0.69226*** | -0.93574*** | -0.00539 | -0.03532*** | -0.03022*** |
|  | (0.01420) | (0.00215) | (0.13971) | (0.11182) | (0.00467) | (0.00279) | (0.00465) |
| 2021 (within) | -0.16038*** | -0.01664*** | 0.34569*** | -0.52138*** | 0.00113 | -0.02316*** | -0.03259*** |
|  | (0.00889) | (0.00141) | (0.08013) | (0.07057) | (0.00263) | (0.00184) | (0.00309) |
| 2022 (within) | -0.10719*** | -0.01010*** | 0.18335*** | -0.26454*** | 0.00167 | -0.01564*** | 0.00069 |
|  | (0.00646) | (0.00102) | (0.05575) | (0.05029) | (0.00183) | (0.00123) | (0.00194) |
| 2020 (between) | -0.29947*** | -0.03780*** | 0.82302*** | -0.53247 | -0.00707 | -0.04038*** | -0.04159*** |
|  | (0.04189) | (0.00500) | (0.30354) | (0.35674) | (0.00706) | (0.00885) | (0.00809) |
| 2021 (between) | -0.22985*** | -0.02714*** | 0.46920* | -0.75456*** | 0.00014 | -0.02528*** | -0.03288*** |
|  | (0.03799) | (0.00469) | (0.24031) | (0.24507) | (0.00645) | (0.00803) | (0.00805) |
| 2022 (between) | -0.12614*** | -0.01380*** | 0.27695 | -0.43313* | 0.00070 | -0.01807** | -0.00440 |
|  | (0.03708) | (0.00462) | (0.23687) | (0.26015) | (0.00651) | (0.00776) | (0.00759) |
| Region North | 0.32176*** | 0.03047*** | -0.64897*** | -2.17268*** | -0.01782*** | 0.05257*** | 0.03282*** |
|  | (0.03343) | (0.00411) | (0.21913) | (0.35081) | (0.00492) | (0.00716) | (0.00710) |
| Region Central | 0.31077*** | 0.03221*** | -0.85345*** | -1.32314*** | 0.00221 | 0.03962*** | 0.03379*** |
|  | (0.02632) | (0.00333) | (0.16761) | (0.37772) | (0.00452) | (0.00540) | (0.00547) |
| Region South | 0.09748*** | 0.00769*** | -0.75019*** | -2.47364*** | -0.01264*** | 0.02830*** | 0.02627*** |
|  | (0.02401) | (0.00291) | (0.17000) | (0.30623) | (0.00438) | (0.00565) | (0.00576) |
| Region Zealand | 0.09748*** | 0.01045*** | 0.36253* | -0.66748*** | -0.01795*** | 0.01671*** | -0.00281 |
|  | (0.02368) | (0.00278) | (0.18676) | (0.25495) | (0.00408) | (0.00647) | (0.00542) |
| Rural area | -0.05397*** | -0.00542*** | 0.13142 | 0.09977 | -0.00379 | -0.00986** | -0.00437 |
|  | (0.01707) | (0.00205) | (0.11634) | (0.11909) | (0.00258) | (0.00407) | (0.00397) |
| Urban area | 0.01260 | 0.00099 | 0.19541 | 0.54964*** | 0.00039 | -0.00742* | -0.01434*** |
|  | (0.02011) | (0.00226) | (0.12942) | (0.14668) | (0.00343) | (0.00446) | (0.00423) |
| Risk of GP shortage | -0.07617*** | -0.00900*** | 0.08603 | -0.68590*** | -0.00379 | 0.01049 | 0.00074 |
|  | (0.02773) | (0.00307) | (0.21730) | (0.22854) | (0.00406) | (0.00709) | (0.00589) |
| Constant | 2.11324*** | 0.07483*** | -2.14807*** | 2.24338*** | 0.04740*** | 0.13284*** | 0.12231*** |
|  | (0.07995) | (0.00929) | (0.60760) | (0.63962) | (0.01472) | (0.01777) | (0.01564) |
|  |  |  |  |  |  |  |  |
| Observations | 3,286 | 3,286 | 3,286 | 3,286 | 3,286 | 3,286 | 3,286 |
| No. of practices | 1,248 | 1,248 | 1,248 | 1,248 | 1,248 | 1,248 | 1,248 |

Note: The columns contain coefficients from within-between regressions estimated by maximum likelihood (MLE). Each of the seven quality indicators (calculated over the first six months of each year t=2020 to 2023) is regressed on financial motivation and patient-oriented altruism from each year t-1 (2019 to 2022), time dummies (reference year 2023) and a set of controls related to patient, practice, and area characteristics (see Appendix C for details). Sum scores for financial motivation and patient-oriented altruism are calculated by weighting each individual survey item according to its factor loading in a confirmatory factor analysis (see Appendix B for factor loadings and details regarding CFA). Regressions are based on an unbalanced sample of 3286 observations from 1248 general practices in Denmark. The number of observations per wave is 755 (2020), 842 (2021), 841 (2022), and 848 (2023). Observations are weighted according to the share of GPs in the practice responding to the motivation survey in that year. Standard errors clustered at the practice level in parentheses.

*p<0.10	**p<0.05	*** p<0.01

# **Appendix K –** **Regression results with motivations dichotomised at the 50th percentile of the distribution**

Table K1. Regression results with motivations dichotomised at the 50th percentile of the distribution

|  | (1) | (2) | (3) | (4) | (5) | (6) | (7) |
| --- | --- | --- | --- | --- | --- | --- | --- |
|  | Average number of prescriptions (over 65) | Share of polypharmacy patients  (over 65) | Average DDDs, NSAIDs without PPI (over 65) | Average DDDs, sleeping medication (18+) | Share of patients with preventive home visit (75+) | Share of patients with annual control for chronic disease (18+) | Share of COPD and asthma patients with spirometry test (18+) |
|  |  |  |  |  |  |  |  |
| Lagged financial motivation (within) | -0.01356***  (0.00510) | -0.00090  (0.00089) | -0.03678  (0.04235) | -0.07375*  (0.04193) | 0.00097  (0.00164) | 0.00426***  (0.00114) | 0.00184  (0.00180) |
|  |  |  |  |  |  |  |  |
| Lagged patient-oriented altruism (within) | -0.00431  (0.00527) | -0.00033  (0.00083) | -0.05757  (0.03899) | 0.07554**  (0.03655) | 0.00032  (0.00138) | 0.00164  (0.00106) | 0.00181  (0.00168) |
|  |  |  |  |  |  |  |  |
| Lagged financial motivation (between) | -0.01876  (0.01651) | -0.00243  (0.00189) | 0.12906  (0.10629) | 0.03039  (0.11205) | 0.01063***  (0.00302) | 0.00974***  (0.00337) | 0.00069  (0.00334) |
|  |  |  |  |  |  |  |  |
| Lagged patient-oriented altruism (between) | 0.02271  (0.01685) | 0.00315  (0.00198) | 0.48996***  (0.10945) | 0.16898  (0.11793) | -0.00209  (0.00307) | -0.00787**  (0.00367) | -0.00594*  (0.00358) |
|  |  |  |  |  |  |  |  |
| List per GP (within) | 0.00001 | 0.00000 | 0.00002 | 0.00009 | -0.00001 | -0.00001** | -0.00000 |
|  | (0.00001) | (0.00000) | (0.00009) | (0.00010) | (0.00001) | (0.00000) | (0.00000) |
| List per GP (between) | 0.00001 | 0.00000 | 0.00039*** | -0.00016 | 0.00000 | 0.00000 | 0.00000 |
|  | (0.00001) | (0.00000) | (0.00011) | (0.00012) | (0.00000) | (0.00000) | (0.00000) |
| SOCIO1 (within) | 2.31715 | 0.33462 | -3.97876 | -10.17846 | 0.63581 | 0.07127 | 0.59653 |
|  | (1.41131) | (0.21723) | (13.75841) | (9.13511) | (0.44172) | (0.23210) | (0.39032) |
| SOCIO2 (within) | 1.32346* | 0.21552* | -7.64854 | -6.83820 | 0.48405* | -0.13698 | 0.00815 |
|  | (0.78989) | (0.12121) | (7.81713) | (6.15134) | (0.27903) | (0.14684) | (0.24934) |
| SOCIO3 (within) | -2.05563* | -0.30604* | 5.61654 | 8.52308 | -0.36282 | -0.34924 | -0.16280 |
|  | (1.10903) | (0.15678) | (8.57648) | (8.69206) | (0.25698) | (0.21437) | (0.28210) |
| SOCIO4 (within) | -1.54652 | -0.39486 | -23.36900 | -15.35647 | -0.30960 | -0.31818 | -0.70215 |
|  | (2.42463) | (0.36121) | (16.72354) | (16.05140) | (0.63064) | (0.41412) | (0.74120) |
| SOCIO5 (within) | 0.38266 | 0.11070 | 10.49646 | 1.20132 | 0.08462 | -0.05847 | 0.03361 |
|  | (0.58212) | (0.10142) | (6.96856) | (4.61001) | (0.22974) | (0.12269) | (0.15457) |
| SOCIO6 (within) | 0.12113 | -0.08466 | 17.47092*** | 5.84668 | -0.00757 | 0.07393 | -0.07047 |
|  | (0.56997) | (0.08900) | (6.02323) | (5.63222) | (0.19467) | (0.12091) | (0.18943) |
| SOCIO7 (within) | 2.78927*** | 0.36615*** | -6.90023 | 19.22984*** | 0.08727 | 0.25805 | 0.09400 |
|  | (0.75350) | (0.11083) | (6.07893) | (7.38163) | (0.20804) | (0.17741) | (0.26021) |
| CCI = 1 (within) | 2.57219** | 0.28168 | -10.16956 | -15.39141 | -0.10824 | 0.69463*** | 0.63597* |
|  | (1.26605) | (0.19795) | (9.56785) | (11.48394) | (0.27342) | (0.24168) | (0.36842) |
| CCI > 1 (within) | 3.00658*** | 0.46580*** | -22.22908*** | 7.85866 | 0.46809* | 0.22785 | 1.08133*** |
|  | (0.93379) | (0.14696) | (8.33722) | (9.59840) | (0.26161) | (0.20036) | (0.32809) |
| SOCIO1 (between) | -1.77420 | 0.37735 | 1.35464 | -67.73145** | 0.29990 | -1.08838* | 0.32877 |
|  | (3.14692) | (0.36046) | (21.21157) | (29.84701) | (0.57468) | (0.59034) | (0.54651) |
| SOCIO2 (between) | 3.14090*** | 0.31610*** | 15.15597*** | -14.25916*** | 0.15326* | -0.08396 | -0.01257 |
|  | (0.61720) | (0.07188) | (4.05340) | (4.06297) | (0.09167) | (0.12413) | (0.11228) |
| SOCIO3 (between) | 2.44068*** | 0.18104** | -8.72324* | 65.69805*** | -0.16182 | 0.16744 | -0.27986** |
|  | (0.59386) | (0.08426) | (4.88529) | (13.21075) | (0.10079) | (0.12308) | (0.12249) |
| SOCIO4 (between) | 0.55332 | 0.26081 | 30.57382** | -23.61225 | -0.51131 | 0.14260 | 0.09581 |
|  | (2.03031) | (0.23216) | (14.60507) | (23.41817) | (0.32479) | (0.43525) | (0.42891) |
| SOCIO5 (between) | 0.32839* | 0.02927 | -0.71814 | -4.03067*** | 0.05664 | -0.00406 | 0.00437 |
|  | (0.17894) | (0.02014) | (1.20960) | (1.48886) | (0.03817) | (0.03631) | (0.03300) |
| SOCIO6 (between) | -2.19524*** | -0.22835*** | 0.30186 | 8.62186*** | 0.01508 | -0.29447*** | -0.01273 |
|  | (0.35890) | (0.04286) | (2.45667) | (2.93630) | (0.05738) | (0.07559) | (0.07282) |
| SOCIO7 (between) | 3.79324*** | 0.53067*** | 19.77522*** | -13.02900*** | -0.12518 | 0.17370 | 0.23846* |
|  | (0.59063) | (0.07473) | (4.72555) | (4.78488) | (0.09830) | (0.14949) | (0.12845) |
| CCI = 1 (between) | 10.66274*** | 1.15531*** | -8.10879 | 2.07496 | -0.09380 | 0.99965*** | 1.19366*** |
|  | (1.16871) | (0.14450) | (7.58660) | (10.84679) | (0.17130) | (0.27733) | (0.22604) |
| CCI > 1 (between) | 3.15705*** | 0.18402 | 6.45617 | 14.97469 | 0.07981 | 0.83927*** | -0.20794 |
|  | (0.98580) | (0.12989) | (6.62695) | (9.22676) | (0.15719) | (0.23168) | (0.20515) |
| 2020 (within) | -0.28078*** | -0.02910*** | 0.70532*** | -0.94526*** | -0.00570 | -0.03582*** | -0.03065*** |
|  | (0.01437) | (0.00216) | (0.13896) | (0.11037) | (0.00482) | (0.00273) | (0.00458) |
| 2021 (within) | -0.15907*** | -0.01656*** | 0.35129*** | -0.51988*** | 0.00109 | -0.02350*** | -0.03280*** |
|  | (0.00889) | (0.00141) | (0.08018) | (0.07086) | (0.00264) | (0.00183) | (0.00309) |
| 2022 (within) | -0.10616*** | -0.01003*** | 0.18209*** | -0.26631*** | 0.00157 | -0.01575*** | 0.00063 |
|  | (0.00641) | (0.00102) | (0.05636) | (0.05044) | (0.00181) | (0.00124) | (0.00193) |
| 2020 (between) | -0.30276*** | -0.03839*** | 0.72590** | -0.55820 | -0.00587 | -0.03834*** | -0.04077*** |
|  | (0.04161) | (0.00496) | (0.30123) | (0.35479) | (0.00703) | (0.00888) | (0.00804) |
| 2021 (between) | -0.22829*** | -0.02697*** | 0.46260* | -0.75500*** | 0.00025 | -0.02550*** | -0.03288*** |
|  | (0.03786) | (0.00468) | (0.24122) | (0.24551) | (0.00642) | (0.00803) | (0.00804) |
| 2022 (between) | -0.12617*** | -0.01392*** | 0.28856 | -0.42249 | 0.00208 | -0.01707** | -0.00473 |
|  | (0.03685) | (0.00458) | (0.23579) | (0.25939) | (0.00655) | (0.00782) | (0.00763) |
| Region North | 0.32112*** | 0.03044*** | -0.65665*** | -2.17893*** | -0.01821*** | 0.05233*** | 0.03304*** |
|  | (0.03351) | (0.00411) | (0.21896) | (0.35139) | (0.00486) | (0.00716) | (0.00709) |
| Region Central | 0.31133*** | 0.03230*** | -0.84395*** | -1.31804*** | 0.00112 | 0.03870*** | 0.03352*** |
|  | (0.02630) | (0.00334) | (0.16888) | (0.37771) | (0.00448) | (0.00539) | (0.00549) |
| Region South | 0.09732*** | 0.00775*** | -0.75204*** | -2.47724*** | -0.01386*** | 0.02737*** | 0.02638*** |
|  | (0.02393) | (0.00290) | (0.17121) | (0.30672) | (0.00434) | (0.00565) | (0.00584) |
| Region Zealand | 0.09708*** | 0.01044*** | 0.35344* | -0.67403*** | -0.01829*** | 0.01645** | -0.00253 |
|  | (0.02375) | (0.00278) | (0.18757) | (0.25533) | (0.00403) | (0.00642) | (0.00544) |
| Rural area | -0.05434*** | -0.00547*** | 0.12507 | 0.09836 | -0.00390 | -0.00985** | -0.00438 |
|  | (0.01698) | (0.00204) | (0.11766) | (0.11928) | (0.00259) | (0.00407) | (0.00398) |
| Urban area | 0.01419 | 0.00114 | 0.19789 | 0.55246*** | 0.00045 | -0.00768* | -0.01450*** |
|  | (0.02007) | (0.00226) | (0.13019) | (0.14661) | (0.00343) | (0.00445) | (0.00425) |
| Risk of GP shortage | -0.07537*** | -0.00894*** | 0.07989 | -0.68394*** | -0.00482 | 0.00975 | 0.00047 |
|  | (0.02776) | (0.00307) | (0.21663) | (0.22672) | (0.00404) | (0.00708) | (0.00583) |
| Constant | 2.11900*** | 0.07667*** | -0.68298 | 2.72096*** | 0.04210*** | 0.11836*** | 0.10796*** |
|  | (0.06495) | (0.00776) | (0.45522) | (0.50256) | (0.01150) | (0.01474) | (0.01320) |
|  |  |  |  |  |  |  |  |
| Observations | 3,286 | 3,286 | 3,286 | 3,286 | 3,286 | 3,286 | 3,286 |
| No. of practices | 1,248 | 1,248 | 1,248 | 1,248 | 1,248 | 1,248 | 1,248 |

Note: The columns contain coefficients from within-between regressions estimated by maximum likelihood (MLE). Each of the seven quality indicators (calculated over the first six months of each year t=2020 to 2023) is regressed on financial motivation and patient-oriented altruism from each year t-1 (2019 to 2022), time dummies (reference year 2023) and a set of controls related to patient, practice, and area characteristics (see Appendix C for details). Financial motivation and patient-oriented altruism are binary variables that are equal to one if the average level of motivation within a practice is above the sample median for that motivation in a given year. Regressions are based on an unbalanced sample of 3286 observations from 1248 general practices in Denmark. The number of observations per wave is 755 (2020), 842 (2021), 841 (2022), and 848 (2023). Observations are weighted according to the share of GPs in the practice responding to the motivation survey in that year. Standard errors clustered at the practice level in parentheses.

*p<0.10	**p<0.05	*** p<0.01

# **Appendix L – Regression results with motivations dichotomised at the 75th percentile of the distribution**

Table L1. Regression results with motivations dichotomised at the 75th percentile of the distribution

|  | (1) | (2) | (3) | (4) | (5) | (6) | (7) |
| --- | --- | --- | --- | --- | --- | --- | --- |
|  | Average number of prescriptions (over 65) | Share of polypharmacy patients  (over 65) | Average DDDs, NSAIDs without PPI (over 65) | Average DDDs, sleeping medication (18+) | Share of patients with preventive home visit (75+) | Share of patients with annual control for chronic disease (18+) | Share of COPD and asthma patients with spirometry test (18+) |
|  |  |  |  |  |  |  |  |
| Lagged financial motivation (within) | 0.00158  (0.00642) | -0.00067  (0.00106) | -0.02787  (0.05161) | -0.05519  (0.05004) | 0.00168  (0.00174) | 0.00291*  (0.00158) | -0.00114  (0.00202) |
|  |  |  |  |  |  |  |  |
| Lagged patient-oriented altruism (within) | 0.00121  (0.00549) | -0.00084  (0.00091) | -0.05833  (0.04554) | 0.03214  (0.04220) | 0.00120  (0.00200) | 0.00070  (0.00117) | 0.00206  (0.00195) |
|  |  |  |  |  |  |  |  |
| Lagged financial motivation (between) | -0.00135  (0.01909) | -0.00123  (0.00221) | 0.37381***  (0.13016) | 0.24673*  (0.13300) | 0.01036***  (0.00361) | 0.00833**  (0.00385) | -0.00010  (0.00379) |
|  |  |  |  |  |  |  |  |
| Lagged patient-oriented altruism (between) | 0.00212  (0.01818) | 0.00118  (0.00217) | 0.58683***  (0.12744) | 0.17974  (0.12909) | -0.00445  (0.00307) | -0.01758***  (0.00399) | -0.00721**  (0.00365) |
|  |  |  |  |  |  |  |  |
| List per GP (within) | 0.00001 | 0.00000 | 0.00002 | 0.00009 | -0.00001 | -0.00001** | -0.00000 |
|  | (0.00001) | (0.00000) | (0.00009) | (0.00010) | (0.00001) | (0.00000) | (0.00000) |
| List per GP (between) | 0.00001 | 0.00000 | 0.00037*** | -0.00017 | 0.00000 | 0.00000 | 0.00000 |
|  | (0.00001) | (0.00000) | (0.00011) | (0.00012) | (0.00000) | (0.00000) | (0.00000) |
| SOCIO1 (within) | 2.28591 | 0.33958 | -3.71825 | -10.16991 | 0.62080 | 0.05736 | 0.59917 |
|  | (1.41374) | (0.21716) | (13.71754) | (9.18846) | (0.44182) | (0.23238) | (0.39098) |
| SOCIO2 (within) | 1.32644* | 0.21715* | -7.57545 | -6.90945 | 0.48194* | -0.13284 | -0.00089 |
|  | (0.79643) | (0.12132) | (7.82275) | (6.15658) | (0.27679) | (0.14607) | (0.24893) |
| SOCIO3 (within) | -2.07539* | -0.30650* | 5.53574 | 8.56009 | -0.36021 | -0.34427 | -0.15781 |
|  | (1.10552) | (0.15659) | (8.58093) | (8.72690) | (0.25717) | (0.21655) | (0.28116) |
| SOCIO4 (within) | -1.56387 | -0.39116 | -23.48398 | -14.19112 | -0.32208 | -0.34776 | -0.66863 |
|  | (2.41584) | (0.36242) | (16.68338) | (16.12990) | (0.62424) | (0.41683) | (0.73845) |
| SOCIO5 (within) | 0.38178 | 0.10965 | 10.37806 | 1.36982 | 0.08684 | -0.05352 | 0.03478 |
|  | (0.58212) | (0.10080) | (6.92817) | (4.59565) | (0.23017) | (0.12530) | (0.15317) |
| SOCIO6 (within) | 0.14526 | -0.08247 | 17.60458*** | 6.02397 | -0.01172 | 0.06017 | -0.07209 |
|  | (0.57298) | (0.08912) | (6.00578) | (5.65495) | (0.19433) | (0.12048) | (0.18914) |
| SOCIO7 (within) | 2.69794*** | 0.36013*** | -7.20386 | 18.80169** | 0.09489 | 0.28937 | 0.10726 |
|  | (0.75399) | (0.11031) | (6.02710) | (7.40444) | (0.20456) | (0.17992) | (0.25984) |
| CCI = 1 (within) | 2.70552** | 0.28859 | -9.34172 | -15.90123 | -0.11241 | 0.65601*** | 0.60778* |
|  | (1.25470) | (0.19671) | (9.53214) | (11.39817) | (0.27607) | (0.24012) | (0.36690) |
| CCI > 1 (within) | 3.00543*** | 0.46498*** | -22.07428*** | 7.39160 | 0.47331* | 0.23706 | 1.07044*** |
|  | (0.93412) | (0.14744) | (8.30986) | (9.61034) | (0.26194) | (0.20119) | (0.32684) |
| SOCIO1 (between) | -1.67155 | 0.37672 | 2.96443 | -65.68709** | 0.35393 | -1.00635* | 0.34135 |
|  | (3.15655) | (0.36077) | (21.20977) | (29.74129) | (0.57207) | (0.58190) | (0.54740) |
| SOCIO2 (between) | 3.10390*** | 0.31467*** | 14.52415*** | -14.81994*** | 0.14938 | -0.08645 | -0.01122 |
|  | (0.61920) | (0.07177) | (4.00148) | (4.04462) | (0.09170) | (0.12254) | (0.11315) |
| SOCIO3 (between) | 2.44355*** | 0.17832** | -7.73537 | 66.44745*** | -0.15718 | 0.15593 | -0.28169** |
|  | (0.59679) | (0.08397) | (4.79739) | (13.23335) | (0.09927) | (0.12212) | (0.12271) |
| SOCIO4 (between) | 0.56336 | 0.26174 | 31.39927** | -23.07447 | -0.51060 | 0.12539 | 0.09106 |
|  | (2.03468) | (0.23212) | (14.38045) | (23.42207) | (0.32439) | (0.43428) | (0.43056) |
| SOCIO5 (between) | 0.33709* | 0.03043 | -1.01187 | -4.19776*** | 0.05580 | 0.00259 | 0.00581 |
|  | (0.17922) | (0.02010) | (1.20690) | (1.47408) | (0.03798) | (0.03595) | (0.03320) |
| SOCIO6 (between) | -2.17332*** | -0.22569*** | -0.42920 | 8.25365*** | 0.01692 | -0.27365*** | -0.00758 |
|  | (0.36145) | (0.04284) | (2.45652) | (2.93290) | (0.05747) | (0.07445) | (0.07228) |
| SOCIO7 (between) | 3.82697*** | 0.53308*** | 19.79695*** | -12.70641*** | -0.14227 | 0.15677 | 0.24015* |
|  | (0.58948) | (0.07478) | (4.64397) | (4.74487) | (0.09730) | (0.14616) | (0.12733) |
| CCI = 1 (between) | 10.65512*** | 1.15546*** | -7.01938 | 2.58355 | -0.10459 | 0.96872*** | 1.18325*** |
|  | (1.16919) | (0.14431) | (7.54273) | (10.82824) | (0.17028) | (0.27136) | (0.22561) |
| CCI > 1 (between) | 3.17398*** | 0.18756 | 7.00432 | 14.95966 | 0.09058 | 0.84710*** | -0.21556 |
|  | (0.98102) | (0.12942) | (6.58256) | (9.19312) | (0.15679) | (0.22740) | (0.20329) |
| 2020 (within) | -0.28090*** | -0.02906*** | 0.70582*** | -0.94150*** | -0.00571 | -0.03575*** | -0.03075*** |
|  | (0.01439) | (0.00215) | (0.13795) | (0.11080) | (0.00483) | (0.00275) | (0.00457) |
| 2021 (within) | -0.16043*** | -0.01660*** | 0.34850*** | -0.52164*** | 0.00111 | -0.02319*** | -0.03262*** |
|  | (0.00888) | (0.00140) | (0.08037) | (0.07042) | (0.00265) | (0.00184) | (0.00309) |
| 2022 (within) | -0.10679*** | -0.00998*** | 0.18204*** | -0.26010*** | 0.00144 | -0.01581*** | 0.00075 |
|  | (0.00643) | (0.00101) | (0.05711) | (0.04979) | (0.00183) | (0.00123) | (0.00193) |
| 2020 (between) | -0.30221*** | -0.03838*** | 0.69202** | -0.57181 | -0.00584 | -0.03757*** | -0.04045*** |
|  | (0.04162) | (0.00496) | (0.29871) | (0.35400) | (0.00702) | (0.00877) | (0.00805) |
| 2021 (between) | -0.22989*** | -0.02718*** | 0.51525** | -0.73112*** | 0.00076 | -0.02612*** | -0.03316*** |
|  | (0.03830) | (0.00471) | (0.23972) | (0.24257) | (0.00639) | (0.00798) | (0.00808) |
| 2022 (between) | -0.12835*** | -0.01410*** | 0.26861 | -0.43615* | 0.00093 | -0.01895** | -0.00477 |
|  | (0.03719) | (0.00461) | (0.23840) | (0.26238) | (0.00654) | (0.00768) | (0.00752) |
| Region North | 0.32271*** | 0.03062*** | -0.64212*** | -2.17131*** | -0.01808*** | 0.05270*** | 0.03291*** |
|  | (0.03349) | (0.00411) | (0.21811) | (0.35160) | (0.00489) | (0.00714) | (0.00708) |
| Region Central | 0.31038*** | 0.03218*** | -0.85894*** | -1.32824*** | 0.00203 | 0.04025*** | 0.03377*** |
|  | (0.02640) | (0.00334) | (0.16662) | (0.37783) | (0.00449) | (0.00535) | (0.00547) |
| Region South | 0.09862*** | 0.00785*** | -0.78045*** | -2.48949*** | -0.01301*** | 0.02944*** | 0.02671*** |
|  | (0.02404) | (0.00291) | (0.16914) | (0.30599) | (0.00432) | (0.00561) | (0.00580) |
| Region Zealand | 0.09861*** | 0.01059*** | 0.34042* | -0.67536*** | -0.01803*** | 0.01758*** | -0.00234 |
|  | (0.02377) | (0.00279) | (0.18401) | (0.25350) | (0.00404) | (0.00632) | (0.00542) |
| Rural area | -0.05445*** | -0.00548*** | 0.13011 | 0.09881 | -0.00373 | -0.00971** | -0.00445 |
|  | (0.01703) | (0.00205) | (0.11602) | (0.11931) | (0.00257) | (0.00401) | (0.00397) |
| Urban area | 0.01221 | 0.00096 | 0.20072 | 0.55117*** | 0.00034 | -0.00821* | -0.01454*** |
|  | (0.02013) | (0.00226) | (0.12891) | (0.14608) | (0.00343) | (0.00441) | (0.00423) |
| Risk of GP shortage | -0.07736*** | -0.00915*** | 0.07579 | -0.69389*** | -0.00377 | 0.01087 | 0.00056 |
|  | (0.02769) | (0.00308) | (0.21139) | (0.22561) | (0.00403) | (0.00700) | (0.00587) |
| Constant | 2.11800*** | 0.07662*** | -0.52512 | 2.76235*** | 0.04450*** | 0.11848*** | 0.10650*** |
|  | (0.06458) | (0.00776) | (0.44729) | (0.50474) | (0.01142) | (0.01466) | (0.01327) |
|  |  |  |  |  |  |  |  |
| Observations | 3,286 | 3,286 | 3,286 | 3,286 | 3,286 | 3,286 | 3,286 |
| No. of practices | 1,248 | 1,248 | 1,248 | 1,248 | 1,248 | 1,248 | 1,248 |

Note: The columns contain coefficients from within-between regressions estimated by maximum likelihood (MLE). Each of the seven quality indicators (calculated over the first six months of each year t=2020 to 2023) is regressed on financial motivation and patient-oriented altruism from each year t-1 (2019 to 2022), time dummies (reference year 2023) and a set of controls related to patient, practice, and area characteristics (see Appendix C for details). Financial motivation and patient-oriented altruism are binary variables that are equal to one if the average level of motivation within a practice is above the 75^th^ percentile for that motivation in a given year. Regressions are based on an unbalanced sample of 3286 observations from 1248 general practices in Denmark. The number of observations per wave is 755 (2020), 842 (2021), 841 (2022), and 848 (2023). Observations are weighted according to the share of GPs in the practice responding to the motivation survey in that year. Standard errors clustered at the practice level in parentheses. *p<0.10	**p<0.05	*** p<0.01

# **Appendix M – Regression results where “do not know/do not want to answer” responses are treated as missing values**

Table M1. Regression results where “do not know/do not want to answer” responses are treated as missing values

|  | (1) | (2) | (3) | (4) | (5) | (6) | (7) |
| --- | --- | --- | --- | --- | --- | --- | --- |
|  | Average number of prescriptions (over 65) | Share of polypharmacy patients  (over 65) | Average DDDs, NSAIDs without PPI (over 65) | Average DDDs, sleeping medication (18+) | Share of patients with preventive home visit (75+) | Share of patients with annual control for chronic disease (18+) | Share of COPD and asthma patients with spirometry test (18+) |
|  |  |  |  |  |  |  |  |
| Lagged financial motivation (within) | -0.01905  (0.01612) | -0.00173  (0.00271) | -0.02796  (0.14036) | -0.30217**  (0.12694) | 0.00253  (0.00437) | 0.01078***  (0.00396) | -0.00410  (0.00530) |
|  |  |  |  |  |  |  |  |
| Lagged patient-oriented altruism (within) | 0.02490  (0.02187) | 0.00092  (0.00344) | -0.15843  (0.18271) | 0.06433  (0.16494) | 0.00701  (0.00760) | 0.00585  (0.00597) | 0.00785  (0.00767) |
|  |  |  |  |  |  |  |  |
| Lagged financial motivation (between) | -0.03228  (0.03444) | -0.00416  (0.00404) | 0.54325**  (0.24647) | 0.22808  (0.27186) | 0.02654***  (0.00767) | 0.02638***  (0.00729) | 0.00532  (0.00765) |
|  |  |  |  |  |  |  |  |
| Lagged patient-oriented altruism (between) | 0.04559  (0.06282) | 0.00807  (0.00714) | 1.86536***  (0.43407) | 0.65473  (0.42675) | -0.01831*  (0.01104) | -0.04062***  (0.01316) | -0.02129*  (0.01222) |
|  |  |  |  |  |  |  |  |
| List per GP (within) | 0.00001 | 0.00000 | 0.00004 | 0.00008 | -0.00001 | -0.00001** | -0.00000 |
|  | (0.00001) | (0.00000) | (0.00009) | (0.00010) | (0.00001) | (0.00000) | (0.00000) |
| List per GP (between) | 0.00001 | 0.00000 | 0.00038*** | -0.00017 | 0.00000 | 0.00000 | 0.00000 |
|  | (0.00001) | (0.00000) | (0.00011) | (0.00012) | (0.00000) | (0.00000) | (0.00000) |
| SOCIO1 (within) | 2.31280 | 0.36124* | -7.24343 | -8.64655 | 0.76274 | 0.01251 | 0.68387* |
|  | (1.42994) | (0.21616) | (14.05177) | (9.54528) | (0.46486) | (0.24015) | (0.39721) |
| SOCIO2 (within) | 1.47732* | 0.18346 | -8.99801 | -6.47955 | 0.49734* | -0.18600 | -0.06750 |
|  | (0.82138) | (0.12596) | (8.00304) | (6.26821) | (0.29291) | (0.15239) | (0.25656) |
| SOCIO3 (within) | -1.48241 | -0.21066 | 4.62308 | 8.05415 | -0.33432 | -0.34505 | -0.13313 |
|  | (1.13614) | (0.16016) | (8.76348) | (9.14824) | (0.26518) | (0.22035) | (0.28621) |
| SOCIO4 (within) | -3.60490 | -0.52171 | -24.86058 | -20.58039 | -0.42670 | -0.18918 | -0.06951 |
|  | (2.33533) | (0.38242) | (18.56476) | (17.31184) | (0.69001) | (0.46266) | (0.73524) |
| SOCIO5 (within) | 0.60563 | 0.15768* | 9.71829 | -0.03867 | 0.13268 | -0.00140 | 0.01429 |
|  | (0.58203) | (0.09378) | (7.96643) | (4.41551) | (0.24893) | (0.12987) | (0.16509) |
| SOCIO6 (within) | 0.13100 | -0.06667 | 18.39540*** | 6.76962 | 0.00738 | 0.07597 | -0.14014 |
|  | (0.58687) | (0.08988) | (6.14684) | (5.82001) | (0.20096) | (0.12429) | (0.19091) |
| SOCIO7 (within) | 2.71177*** | 0.35186*** | -9.27636 | 20.09785*** | 0.08805 | 0.23930 | 0.03764 |
|  | (0.76585) | (0.11183) | (6.28969) | (7.60726) | (0.21369) | (0.18213) | (0.26222) |
| CCI = 1 (within) | 2.41665* | 0.27176 | -8.70870 | -15.49281 | -0.06642 | 0.57445** | 0.66378* |
|  | (1.36568) | (0.20807) | (9.84633) | (11.57225) | (0.28935) | (0.23660) | (0.36980) |
| CCI > 1 (within) | 2.94479*** | 0.45906*** | -23.53573*** | 5.79585 | 0.51276* | 0.30032 | 1.20570*** |
|  | (0.97324) | (0.15067) | (8.57362) | (9.77002) | (0.27115) | (0.20270) | (0.33822) |
| SOCIO1 (between) | -1.16813 | 0.39267 | 10.59979 | -62.09556** | 0.39404 | -1.13122* | 0.20820 |
|  | (3.19034) | (0.36633) | (21.37043) | (30.05667) | (0.58069) | (0.59895) | (0.55210) |
| SOCIO2 (between) | 3.03096*** | 0.30670*** | 14.75416*** | -14.55173*** | 0.14341 | -0.10417 | -0.03876 |
|  | (0.62772) | (0.07408) | (4.05749) | (4.12868) | (0.09213) | (0.12577) | (0.11456) |
| SOCIO3 (between) | 2.55812*** | 0.18552** | -8.53531* | 66.73880*** | -0.16463 | 0.18467 | -0.27557** |
|  | (0.60882) | (0.08602) | (4.85532) | (13.25606) | (0.10314) | (0.12425) | (0.12721) |
| SOCIO4 (between) | 0.71589 | 0.29782 | 33.29861** | -22.92483 | -0.47304 | 0.19285 | 0.23759 |
|  | (2.04046) | (0.23348) | (14.45512) | (23.29498) | (0.32729) | (0.44185) | (0.42626) |
| SOCIO5 (between) | 0.31567* | 0.02942 | -0.80063 | -4.06974*** | 0.06384* | -0.00902 | -0.00670 |
|  | (0.18166) | (0.02067) | (1.20025) | (1.50088) | (0.03851) | (0.03617) | (0.03312) |
| SOCIO6 (between) | -2.21984*** | -0.22804*** | 0.17132 | 8.04618*** | 0.02376 | -0.29583*** | -0.00742 |
|  | (0.36514) | (0.04382) | (2.49222) | (2.95004) | (0.05754) | (0.07674) | (0.07358) |
| SOCIO7 (between) | 3.81680*** | 0.53031*** | 18.80747*** | -13.19145*** | -0.14105 | 0.15904 | 0.25222* |
|  | (0.60386) | (0.07737) | (4.79052) | (4.86655) | (0.09972) | (0.15102) | (0.13161) |
| CCI = 1 (between) | 10.46871*** | 1.14287*** | -8.49638 | 1.05031 | -0.07250 | 0.96936*** | 1.22537*** |
|  | (1.18588) | (0.14719) | (7.69177) | (10.88693) | (0.17292) | (0.28051) | (0.22641) |
| CCI > 1 (between) | 3.24033*** | 0.19455 | 7.61218 | 16.44448* | 0.08193 | 0.85661*** | -0.25897 |
|  | (0.99146) | (0.13247) | (6.62411) | (9.34798) | (0.15756) | (0.23492) | (0.20626) |
| 2020 (within) | -0.27697*** | -0.02850*** | 0.73218*** | -0.90911*** | -0.00592 | -0.03550*** | -0.03213*** |
|  | (0.01459) | (0.00217) | (0.14690) | (0.11222) | (0.00496) | (0.00286) | (0.00477) |
| 2021 (within) | -0.15752*** | -0.01619*** | 0.36808*** | -0.52189*** | 0.00075 | -0.02295*** | -0.03309*** |
|  | (0.00899) | (0.00140) | (0.08346) | (0.07106) | (0.00281) | (0.00189) | (0.00317) |
| 2022 (within) | -0.10591*** | -0.00971*** | 0.18885*** | -0.25361*** | 0.00177 | -0.01594*** | -0.00050 |
|  | (0.00662) | (0.00103) | (0.05681) | (0.05206) | (0.00190) | (0.00127) | (0.00192) |
| 2020 (between) | -0.29788*** | -0.03758*** | 0.77433** | -0.60390* | -0.00744 | -0.04037*** | -0.03968*** |
|  | (0.04274) | (0.00510) | (0.31117) | (0.35952) | (0.00710) | (0.00897) | (0.00814) |
| 2021 (between) | -0.21760*** | -0.02565*** | 0.43949* | -0.76062*** | 0.00100 | -0.02303*** | -0.03108*** |
|  | (0.03900) | (0.00485) | (0.24467) | (0.25308) | (0.00657) | (0.00829) | (0.00827) |
| 2022 (between) | -0.11776*** | -0.01296*** | 0.31790 | -0.36278 | 0.00286 | -0.01634** | -0.00361 |
|  | (0.03708) | (0.00466) | (0.23979) | (0.25952) | (0.00647) | (0.00786) | (0.00766) |
| Region North | 0.31721*** | 0.03065*** | -0.64527*** | -2.18948*** | -0.01623*** | 0.05214*** | 0.03270*** |
|  | (0.03370) | (0.00419) | (0.22035) | (0.35483) | (0.00488) | (0.00721) | (0.00716) |
| Region Central | 0.30368*** | 0.03209*** | -0.87253*** | -1.36071*** | 0.00187 | 0.03781*** | 0.03138*** |
|  | (0.02685) | (0.00343) | (0.17073) | (0.38078) | (0.00455) | (0.00551) | (0.00563) |
| Region South | 0.09072*** | 0.00741** | -0.77738*** | -2.52146*** | -0.01264*** | 0.02711*** | 0.02509*** |
|  | (0.02431) | (0.00297) | (0.17388) | (0.30949) | (0.00444) | (0.00570) | (0.00597) |
| Region Zealand | 0.09527*** | 0.01046*** | 0.32648* | -0.70652*** | -0.01707*** | 0.01620** | -0.00299 |
|  | (0.02367) | (0.00280) | (0.18749) | (0.25504) | (0.00405) | (0.00650) | (0.00553) |
| Rural area | -0.05435*** | -0.00546*** | 0.11780 | 0.09357 | -0.00410 | -0.01051** | -0.00417 |
|  | (0.01715) | (0.00206) | (0.11811) | (0.12058) | (0.00261) | (0.00409) | (0.00403) |
| Urban area | 0.01151 | 0.00100 | 0.13343 | 0.52307*** | -0.00026 | -0.00790* | -0.01367*** |
|  | (0.02039) | (0.00230) | (0.13175) | (0.14733) | (0.00345) | (0.00445) | (0.00428) |
| Risk of GP shortage | -0.07593*** | -0.00897*** | 0.08269 | -0.70840*** | -0.00479 | 0.00971 | -0.00009 |
|  | (0.02797) | (0.00310) | (0.21516) | (0.22963) | (0.00414) | (0.00717) | (0.00590) |
| Constant | 2.10669*** | 0.07265*** | -1.91852*** | 2.29319*** | 0.04568*** | 0.13857*** | 0.11940*** |
|  | (0.07813) | (0.00912) | (0.56569) | (0.60570) | (0.01336) | (0.01694) | (0.01521) |
|  |  |  |  |  |  |  |  |
| Observations | 3,175 | 3,175 | 3,175 | 3,175 | 3,175 | 3,175 | 3,175 |
| No. of practices | 1,230 | 1,230 | 1,230 | 1,230 | 1,230 | 1,230 | 1,230 |

Note: The columns contain coefficients from within-between regressions estimated by maximum likelihood (MLE). Each of the seven quality indicators (calculated over the first six months of each year t=2020 to 2023) is regressed on financial motivation and patient-oriented altruism from each year t-1 (2019 to 2022), time dummies (reference year 2023) and a set of controls related to patient, practice, and area characteristics (see Appendix C for details). The motivation scores of GPs who responded to any survey items with “do not know/do not want to answer” are excluded from the calculation of the practice-level average motivations in this analysis. Regressions are based on an unbalanced sample of 3175 observations from 1230 general practices in Denmark. The number of observations per wave is 729 (2020), 811 (2021), 816 (2022), 819 (2023). Observations are weighted according to the share of GPs in the practice responding to the motivation survey in that year. Standard errors clustered at the practice level in parentheses. *p<0.10	**p<0.05	*** p<0.01

# **Appendix N – Regression results when observations with 2020 motivation and 2021 quality are removed**

Table N1. Regression results when observations with 2020 motivation and 2021 quality are removed

|  | (1) | (2) | (3) | (4) | (5) | (6) | (7) |
| --- | --- | --- | --- | --- | --- | --- | --- |
|  | Average number of prescriptions (over 65) | Share of polypharmacy patients  (over 65) | Average DDDs, NSAIDs without PPI (over 65) | Average DDDs, sleeping medication (18+) | Share of patients with preventive home visit (75+) | Share of patients with annual control for chronic disease (18+) | Share of COPD and asthma patients with spirometry test (18+) |
|  |  |  |  |  |  |  |  |
| Lagged financial motivation (within) | -0.00755  (0.02108) | 0.00158  (0.00347) | -0.08980  (0.16795) | -0.40629**  (0.18097) | 0.00513  (0.00498) | 0.01564***  (0.00528) | -0.00730  (0.00649) |
|  |  |  |  |  |  |  |  |
| Lagged patient-oriented altruism (within) | -0.00167  (0.02917) | -0.00475  (0.00458) | -0.06385  (0.22457) | 0.11289  (0.22523) | 0.00071  (0.00755) | 0.00997  (0.00758) | 0.00594  (0.00841) |
|  |  |  |  |  |  |  |  |
| Lagged financial motivation (between) | -0.05099  (0.03528) | -0.00537  (0.00422) | 0.40007  (0.26759) | 0.21630  (0.29911) | 0.03157***  (0.00821) | 0.03255***  (0.00754) | 0.00373  (0.00801) |
|  |  |  |  |  |  |  |  |
| Lagged patient-oriented altruism (between) | 0.12080**  (0.06128) | 0.01666**  (0.00710) | 1.87993***  (0.43557) | 0.97457**  (0.44844) | -0.02383**  (0.01131) | -0.04561***  (0.01412) | -0.02357*  (0.01298) |
|  |  |  |  |  |  |  |  |
| List per GP (within) | 0.00000 | -0.00000 | -0.00030*** | -0.00002 | -0.00001 | -0.00001* | -0.00001* |
|  | (0.00002) | (0.00000) | (0.00011) | (0.00012) | (0.00001) | (0.00000) | (0.00001) |
| List per GP (between) | 0.00002 | 0.00000 | 0.00042*** | -0.00021 | 0.00000 | 0.00000 | 0.00000 |
|  | (0.00002) | (0.00000) | (0.00012) | (0.00013) | (0.00000) | (0.00000) | (0.00000) |
| SOCIO1 (within) | 2.25623 | 0.21034 | 1.83531 | -22.50418** | 0.60530 | 0.33131 | 0.17294 |
|  | (1.69640) | (0.24504) | (16.17870) | (10.90227) | (0.42548) | (0.27583) | (0.45266) |
| SOCIO2 (within) | 0.33614 | -0.02945 | -10.12132 | -7.78199 | 0.21634 | -0.18294 | -0.22508 |
|  | (0.89634) | (0.13825) | (7.73662) | (7.08056) | (0.24769) | (0.17547) | (0.29272) |
| SOCIO3 (within) | -1.46375 | -0.09533 | 8.61228 | 16.21494 | -0.45727 | -0.52208* | 0.20980 |
|  | (1.23701) | (0.18267) | (10.31699) | (11.17635) | (0.32118) | (0.29226) | (0.32874) |
| SOCIO4 (within) | 0.09822 | -0.12753 | -12.37731 | -24.35052 | -0.41203 | -0.08810 | -0.59231 |
|  | (2.88625) | (0.39831) | (19.56029) | (19.54123) | (0.65500) | (0.49231) | (0.80999) |
| SOCIO5 (within) | -0.32011 | 0.03770 | 7.96537* | -2.42381 | 0.06917 | 0.00173 | 0.12815 |
|  | (0.69667) | (0.11735) | (4.36593) | (5.86505) | (0.14378) | (0.14703) | (0.17291) |
| SOCIO6 (within) | 0.21119 | -0.06034 | 9.80329* | -0.04318 | 0.02988 | 0.03656 | -0.23873 |
|  | (0.66329) | (0.10640) | (5.66850) | (6.04932) | (0.21482) | (0.15125) | (0.21861) |
| SOCIO7 (within) | 2.14263*** | 0.29532** | -1.48934 | 24.50858*** | 0.20347 | 0.13861 | 0.04294 |
|  | (0.83060) | (0.12154) | (6.28749) | (8.25152) | (0.20574) | (0.18665) | (0.28090) |
| CCI = 1 (within) | 2.33257* | 0.10605 | -24.20751** | -24.88176** | 0.08199 | 0.90894*** | 0.67123 |
|  | (1.39663) | (0.20566) | (10.26032) | (12.37786) | (0.28668) | (0.35194) | (0.45913) |
| CCI > 1 (within) | 1.37236 | 0.26649 | -19.48488* | 9.15909 | 0.48173 | 0.20850 | 1.07081*** |
|  | (1.11125) | (0.17446) | (10.47103) | (9.48055) | (0.34828) | (0.26577) | (0.38226) |
| SOCIO1 (between) | -3.02731 | 0.19692 | 5.58243 | -72.50566** | 0.76426 | -0.82881 | 0.45820 |
|  | (3.12799) | (0.36879) | (22.80986) | (31.01025) | (0.58409) | (0.62317) | (0.58632) |
| SOCIO2 (between) | 3.19196*** | 0.33142*** | 14.91115*** | -16.49477*** | 0.08947 | -0.12430 | 0.01137 |
|  | (0.64625) | (0.07644) | (4.29928) | (4.49431) | (0.09620) | (0.13051) | (0.12060) |
| SOCIO3 (between) | 2.34017*** | 0.17525** | -8.52231* | 67.86162*** | -0.10626 | 0.14641 | -0.27064** |
|  | (0.61186) | (0.08862) | (4.95303) | (12.85166) | (0.10163) | (0.12887) | (0.12940) |
| SOCIO4 (between) | 0.77846 | 0.34169 | 32.24910** | -23.13299 | -0.34673 | 0.49261 | -0.10434 |
|  | (2.07377) | (0.25292) | (15.24395) | (24.44272) | (0.34846) | (0.47919) | (0.45014) |
| SOCIO5 (between) | 0.26243 | 0.01348 | -1.21504 | -4.82845*** | 0.04059 | -0.01229 | 0.00889 |
|  | (0.18153) | (0.02098) | (1.25748) | (1.59126) | (0.03796) | (0.03875) | (0.03582) |
| SOCIO6 (between) | -2.15520*** | -0.22721*** | -0.63775 | 8.78302*** | 0.01108 | -0.26444*** | -0.03547 |
|  | (0.35316) | (0.04314) | (2.45731) | (3.04361) | (0.05718) | (0.07897) | (0.07400) |
| SOCIO7 (between) | 3.90852*** | 0.52652*** | 20.57113*** | -12.43055** | -0.09066 | 0.17739 | 0.22083* |
|  | (0.59749) | (0.07764) | (5.05091) | (5.23604) | (0.10167) | (0.15183) | (0.13411) |
| CCI = 1 (between) | 11.54927*** | 1.25358*** | -13.10678* | 5.38754 | -0.05961 | 1.01109*** | 1.24981*** |
|  | (1.22170) | (0.15122) | (7.86733) | (10.88689) | (0.17790) | (0.28280) | (0.23750) |
| CCI > 1 (between) | 2.40998** | 0.08601 | 8.27059 | 11.46629 | 0.02512 | 0.78625*** | -0.24378 |
|  | (0.99794) | (0.13463) | (6.66118) | (9.72571) | (0.16338) | (0.23667) | (0.21468) |
| 2020 (within) | -0.28919*** | -0.02884*** | 0.59524*** | -0.86724*** | -0.00350 | -0.03813*** | -0.02762*** |
|  | (0.01595) | (0.00239) | (0.14483) | (0.13600) | (0.00417) | (0.00300) | (0.00506) |
| 2021 (within) | 0.13191*** | 0.01826*** | 0.29204 | 0.92603*** | -0.01015 | -0.02189** | -0.00137 |
|  | (0.04729) | (0.00587) | (0.31968) | (0.34421) | (0.00873) | (0.01049) | (0.01045) |
| 2022 (within) | -0.10971*** | -0.01003*** | 0.17265*** | -0.28272*** | 0.00248 | -0.01567*** | 0.00036 |
|  | (0.00738) | (0.00111) | (0.05733) | (0.05689) | (0.00192) | (0.00134) | (0.00207) |
| 2020 (between) | -0.27568*** | -0.03484*** | 0.87916*** | -0.33953 | -0.01270* | -0.04680*** | -0.04183*** |
|  | (0.04205) | (0.00510) | (0.32378) | (0.36948) | (0.00725) | (0.00916) | (0.00843) |
| 2022 (between) | -0.12426*** | -0.01409*** | 0.36117 | -0.34635 | -0.00001 | -0.02003** | -0.00515 |
|  | (0.03736) | (0.00468) | (0.24053) | (0.26790) | (0.00636) | (0.00787) | (0.00786) |
| Region North | 0.33087*** | 0.03139*** | -0.69573*** | -2.28277*** | -0.01923*** | 0.05209*** | 0.03450*** |
|  | (0.03353) | (0.00412) | (0.22637) | (0.34681) | (0.00500) | (0.00740) | (0.00760) |
| Region Central | 0.31918*** | 0.03317*** | -0.85619*** | -1.32952*** | 0.00033 | 0.03894*** | 0.03438*** |
|  | (0.02719) | (0.00351) | (0.17674) | (0.36882) | (0.00457) | (0.00566) | (0.00592) |
| Region South | 0.10201*** | 0.00897*** | -0.76973*** | -2.53143*** | -0.01423*** | 0.02663*** | 0.02852*** |
|  | (0.02427) | (0.00300) | (0.18019) | (0.30189) | (0.00444) | (0.00592) | (0.00620) |
| Region Zealand | 0.10633*** | 0.01187*** | 0.30713 | -0.65762** | -0.01810*** | 0.01660** | -0.00023 |
|  | (0.02473) | (0.00296) | (0.18729) | (0.25893) | (0.00415) | (0.00671) | (0.00578) |
| Rural area | -0.05932*** | -0.00639*** | 0.10843 | 0.10467 | -0.00520** | -0.01201*** | -0.00710* |
|  | (0.01715) | (0.00206) | (0.12028) | (0.12498) | (0.00264) | (0.00413) | (0.00407) |
| Urban area | 0.01496 | 0.00157 | 0.23574* | 0.64425*** | -0.00037 | -0.00961** | -0.02008*** |
|  | (0.02042) | (0.00234) | (0.13203) | (0.15512) | (0.00341) | (0.00460) | (0.00447) |
| Risk of GP shortage | -0.10004*** | -0.01087*** | -0.04800 | -0.82356*** | -0.00436 | 0.01013 | 0.00049 |
|  | (0.02643) | (0.00298) | (0.22010) | (0.24107) | (0.00429) | (0.00717) | (0.00628) |
| Constant | 2.05851*** | 0.06889*** | -1.63375*** | 2.38425*** | 0.04945*** | 0.13484*** | 0.12614*** |
|  | (0.07893) | (0.00932) | (0.58546) | (0.64673) | (0.01339) | (0.01760) | (0.01563) |
|  |  |  |  |  |  |  |  |
| Observations | 2,444 | 2,444 | 2,444 | 2,444 | 2,444 | 2,444 | 2,444 |
| No. of practices | 1,177 | 1,177 | 1,177 | 1,177 | 1,177 | 1,177 | 1,177 |

Note: The columns contain coefficients from within-between regressions estimated by maximum likelihood (MLE). Each of the seven quality indicators (calculated over the first six months of each year t=2020, 2022, and 2023) is regressed on financial motivation and patient-oriented altruism from each year t-1 (2019, 2021, and 2022), time dummies (reference year 2023) and a set of controls related to patient, practice, and area characteristics (see Appendix C for details). Regressions are based on an unbalanced sample of 2444 observations from 1177 general practices in Denmark. The number of observations per wave is 755 (2020), 841 (2022), and 848 (2023). These regressions exclude observations from 2021 (2020 motivations). Observations are weighted according to the share of GPs in the practice responding to the motivation survey in that year. Standard errors clustered at the practice level in parentheses.

*p<0.10	**p<0.05	*** p<0.01

# **Appendix O – Regression results when observations with 2019 motivation and 2020 quality are removed**

Table O1. Regression results when observations with 2019 motivation and 2020 quality are removed

|  | (1) | (2) | (3) | (4) | (5) | (6) | (7) |
| --- | --- | --- | --- | --- | --- | --- | --- |
|  | Average number of prescriptions (over 65) | Share of polypharmacy patients  (over 65) | Average DDDs, NSAIDs without PPI (over 65) | Average DDDs, sleeping medication (18+) | Share of patients with preventive home visit (75+) | Share of patients with annual control for chronic disease (18+) | Share of COPD and asthma patients with spirometry test (18+) |
|  |  |  |  |  |  |  |  |
| Lagged financial motivation (within) | -0.03433*  (0.02062) | -0.00483  (0.00331) | -0.12702  (0.15633) | -0.16519  (0.13861) | 0.00325  (0.00493) | 0.00655  (0.00410) | -0.00531  (0.00623) |
|  |  |  |  |  |  |  |  |
| Lagged patient-oriented altruism (within) | -0.00776  (0.02918) | -0.00176  (0.00429) | -0.28825  (0.24003) | 0.05419  (0.18573) | 0.00440  (0.00987) | -0.00053  (0.00574) | 0.00761  (0.01066) |
|  |  |  |  |  |  |  |  |
| Lagged financial motivation (between) | -0.02199  (0.03677) | -0.00435  (0.00436) | 0.59079**  (0.25448) | 0.25168  (0.25429) | 0.02666***  (0.00877) | 0.02696***  (0.00777) | 0.00683  (0.00878) |
|  |  |  |  |  |  |  |  |
| Lagged patient-oriented altruism (between) | 0.03618  (0.06616) | 0.00612  (0.00763) | 1.88093***  (0.44949) | 0.22879  (0.46572) | -0.02226*  (0.01241) | -0.04083***  (0.01369) | -0.01980  (0.01362) |
|  |  |  |  |  |  |  |  |
| List per GP (within) | 0.00001 | 0.00000 | 0.00019* | 0.00015 | 0.00000 | -0.00001** | -0.00001 |
|  | (0.00002) | (0.00000) | (0.00011) | (0.00012) | (0.00001) | (0.00000) | (0.00000) |
| List per GP (between) | 0.00002 | 0.00000 | 0.00046*** | -0.00010 | 0.00000 | 0.00000 | 0.00000 |
|  | (0.00002) | (0.00000) | (0.00011) | (0.00013) | (0.00000) | (0.00000) | (0.00000) |
| SOCIO1 (within) | 2.99788 | 0.38765 | 0.88050 | 6.08094 | 0.15130 | -0.42627 | 0.92539* |
|  | (1.99155) | (0.30740) | (17.60717) | (12.24766) | (0.71628) | (0.30909) | (0.50615) |
| SOCIO2 (within) | 1.55006 | 0.26283 | -12.69348 | -6.87353 | 0.71337** | -0.26109 | -0.15489 |
|  | (1.04731) | (0.16506) | (10.65790) | (7.83738) | (0.34571) | (0.18178) | (0.31658) |
| SOCIO3 (within) | -1.93112 | -0.44474** | 1.78467 | 9.21208 | -0.38092 | -0.17700 | -0.04036 |
|  | (1.45799) | (0.18920) | (11.05309) | (10.12577) | (0.31525) | (0.20441) | (0.37136) |
| SOCIO4 (within) | -2.85134 | -0.29136 | -32.25638 | 2.53100 | -0.72066 | -0.84771 | -1.53231 |
|  | (2.87966) | (0.51147) | (22.13669) | (22.19736) | (0.84375) | (0.52672) | (0.97068) |
| SOCIO5 (within) | 1.19346* | 0.16272 | 13.94027 | 0.49600 | 0.13885 | 0.01405 | 0.12577 |
|  | (0.61790) | (0.11496) | (10.53401) | (5.01941) | (0.31002) | (0.13099) | (0.20051) |
| SOCIO6 (within) | -0.07307 | -0.08639 | 31.50555*** | 7.98351 | 0.13621 | -0.07669 | 0.06405 |
|  | (0.74807) | (0.10823) | (7.81775) | (6.88707) | (0.22790) | (0.13782) | (0.23718) |
| SOCIO7 (within) | 2.59828*** | 0.30058** | -15.68633** | 14.45806** | -0.08238 | 0.29248 | 0.15316 |
|  | (0.92892) | (0.13847) | (7.68344) | (7.27556) | (0.27125) | (0.18143) | (0.30958) |
| CCI = 1 (within) | 3.45624** | 0.35785 | -8.23715 | -15.15546 | 0.10742 | 0.34962 | 0.76092* |
|  | (1.67399) | (0.25793) | (13.75845) | (13.92258) | (0.33611) | (0.26414) | (0.45154) |
| CCI > 1 (within) | 3.76521*** | 0.60407*** | -27.04407*** | 8.16786 | 0.36436 | 0.43430* | 1.35602*** |
|  | (1.11499) | (0.16871) | (9.56211) | (13.47961) | (0.31968) | (0.22957) | (0.41941) |
| SOCIO1 (between) | -1.98903 | 0.35537 | 7.79481 | -65.18425** | 0.24203 | -1.48623** | 0.25414 |
|  | (3.51539) | (0.40560) | (23.14068) | (30.74026) | (0.67743) | (0.64568) | (0.63227) |
| SOCIO2 (between) | 3.17252*** | 0.32277*** | 11.68713*** | -16.66019*** | 0.11308 | -0.09017 | -0.00642 |
|  | (0.64988) | (0.07780) | (4.02017) | (4.31342) | (0.10008) | (0.12813) | (0.12720) |
| SOCIO3 (between) | 2.61680*** | 0.18761** | -6.82173 | 70.36650*** | -0.12935 | 0.19508 | -0.29660** |
|  | (0.60974) | (0.08545) | (4.65257) | (11.64842) | (0.10619) | (0.12761) | (0.13666) |
| SOCIO4 (between) | 0.30976 | 0.24210 | 33.81694** | -20.48232 | -0.48946 | 0.12758 | 0.18146 |
|  | (2.09374) | (0.23879) | (14.65383) | (22.53870) | (0.35578) | (0.45532) | (0.47086) |
| SOCIO5 (between) | 0.30831* | 0.03325 | -0.09887 | -5.44200*** | 0.06042 | -0.00751 | 0.00799 |
|  | (0.18726) | (0.02158) | (1.25312) | (1.56374) | (0.04391) | (0.03787) | (0.03684) |
| SOCIO6 (between) | -2.44706*** | -0.24874*** | 0.52492 | 9.41984*** | 0.00826 | -0.30477*** | -0.02017 |
|  | (0.38508) | (0.04603) | (2.56621) | (3.08509) | (0.06419) | (0.07946) | (0.07966) |
| SOCIO7 (between) | 3.85248*** | 0.52964*** | 19.08824*** | -15.53819*** | -0.10712 | 0.18687 | 0.24440* |
|  | (0.61298) | (0.07883) | (4.88437) | (4.99160) | (0.11031) | (0.15706) | (0.13848) |
| CCI = 1 (between) | 11.68219*** | 1.24696*** | -6.12265 | 5.28080 | -0.16032 | 1.03583*** | 1.40203*** |
|  | (1.18971) | (0.15054) | (7.35593) | (10.65950) | (0.18917) | (0.29326) | (0.24958) |
| CCI > 1 (between) | 3.15511*** | 0.22417 | 4.61441 | 11.50501 | 0.09217 | 0.87262*** | -0.20218 |
|  | (1.04359) | (0.14081) | (6.76116) | (9.51396) | (0.17124) | (0.24585) | (0.22299) |
| 2020 (within) | 0.07974 | 0.01631** | 0.20524 | -0.07279 | 0.00510 | -0.00042 | 0.01347 |
|  | (0.05452) | (0.00644) | (0.41137) | (0.41743) | (0.01217) | (0.01149) | (0.01253) |
| 2021 (within) | -0.15762*** | -0.01652*** | 0.42967*** | -0.58607*** | 0.00232 | -0.02012*** | -0.03027*** |
|  | (0.01031) | (0.00163) | (0.10540) | (0.07526) | (0.00345) | (0.00211) | (0.00363) |
| 2022 (within) | -0.10348*** | -0.01001*** | 0.22179*** | -0.26485*** | 0.00076 | -0.01499*** | 0.00331 |
|  | (0.00680) | (0.00113) | (0.06491) | (0.05409) | (0.00210) | (0.00130) | (0.00211) |
| 2021 (between) | -0.22204*** | -0.02616*** | 0.53283** | -0.68699*** | 0.00179 | -0.02452*** | -0.03217*** |
|  | (0.03962) | (0.00496) | (0.24760) | (0.24669) | (0.00670) | (0.00832) | (0.00845) |
| 2022 (between) | -0.12649*** | -0.01377*** | 0.39217* | -0.35983 | 0.00169 | -0.01911** | -0.00731 |
|  | (0.03727) | (0.00466) | (0.23685) | (0.26124) | (0.00655) | (0.00779) | (0.00774) |
| Region North | 0.31141*** | 0.02955*** | -0.64683*** | -2.34360*** | -0.01788*** | 0.05277*** | 0.03520*** |
|  | (0.03473) | (0.00431) | (0.22127) | (0.32984) | (0.00524) | (0.00734) | (0.00769) |
| Region Central | 0.31828*** | 0.03364*** | -0.83199*** | -1.41287*** | 0.00119 | 0.03971*** | 0.03734*** |
|  | (0.02708) | (0.00344) | (0.16798) | (0.34556) | (0.00478) | (0.00566) | (0.00614) |
| Region South | 0.10035*** | 0.00824*** | -0.79335*** | -2.64613*** | -0.01277*** | 0.02885*** | 0.02522*** |
|  | (0.02487) | (0.00301) | (0.16914) | (0.28120) | (0.00491) | (0.00586) | (0.00628) |
| Region Zealand | 0.10704*** | 0.01159*** | 0.31043 | -0.86707*** | -0.01742*** | 0.01910*** | -0.00528 |
|  | (0.02435) | (0.00283) | (0.18881) | (0.23980) | (0.00432) | (0.00662) | (0.00592) |
| Rural area | -0.05824*** | -0.00537** | 0.10798 | 0.10701 | -0.00433 | -0.01087*** | -0.00538 |
|  | (0.01771) | (0.00216) | (0.12044) | (0.12500) | (0.00282) | (0.00421) | (0.00426) |
| Urban area | 0.02226 | 0.00169 | 0.14053 | 0.53041*** | 0.00037 | -0.00501 | -0.01347*** |
|  | (0.02098) | (0.00240) | (0.13380) | (0.15482) | (0.00375) | (0.00456) | (0.00462) |
| Risk of GP shortage | -0.08256*** | -0.01012*** | 0.11785 | -0.88435*** | -0.00428 | 0.01393* | 0.00020 |
|  | (0.02953) | (0.00327) | (0.22226) | (0.22373) | (0.00476) | (0.00755) | (0.00678) |
| Constant | 2.08733*** | 0.06976*** | -1.92276*** | 2.69290*** | 0.05333*** | 0.13368*** | 0.11003*** |
|  | (0.07836) | (0.00937) | (0.58809) | (0.64606) | (0.01551) | (0.01798) | (0.01708) |
|  |  |  |  |  |  |  |  |
| Observations | 2,531 | 2,531 | 2,531 | 2,531 | 2,531 | 2,531 | 2,531 |
| No. of practices | 1,177 | 1,177 | 1,177 | 1,177 | 1,177 | 1,177 | 1,177 |

Note: The columns contain coefficients from within-between regressions estimated by maximum likelihood (MLE). Each of the seven quality indicators (calculated over the first six months of each year t=2021 to 2023) is regressed on financial motivation and patient-oriented altruism from each year t-1 (2020 to 2022), time dummies (reference year 2023) and a set of controls related to patient, practice, and area characteristics (see Appendix C for details). Regressions are based on an unbalanced sample of 2531 observations from 1177 general practices in Denmark. The number of observations per wave is 842 (2021), 841 (2022), and 848 (2023). These regressions exclude observations from 2020 (2019 motivations). Observations are weighted according to the share of GPs in the practice responding to the motivation survey in that year. Standard errors clustered at the practice level in parentheses.

*p<0.10	**p<0.05	*** p<0.01

# **Appendix P – Regression results when motivations and quality of care are measured at the same time**

Table P1. Regression results when motivations and quality of care are measured at the same time

|  | (1) | (2) | (3) | (4) | (5) | (6) | (7) |
| --- | --- | --- | --- | --- | --- | --- | --- |
|  | Average number of prescriptions (over 65) | Share of polypharmacy patients  (over 65) | Average DDDs, NSAIDs without PPI (over 65) | Average DDDs, sleeping medication (18+) | Share of patients with preventive home visit (75+) | Share of patients with annual control for chronic disease (18+) | Share of COPD and asthma patients with spirometry test (18+) |
|  |  |  |  |  |  |  |  |
| Lagged financial motivation (within) | 0.00293  (0.01579) | -0.00226  (0.00246) | -0.03353  (0.14732) | -0.26256**  (0.13306) | -0.00100  (0.00505) | 0.00569*  (0.00301) | -0.01098*  (0.00623) |
|  |  |  |  |  |  |  |  |
| Lagged patient-oriented altruism (within) | 0.00688  (0.02095) | -0.00218  (0.00351) | 0.10658  (0.18873) | 0.08870  (0.16194) | 0.00392  (0.00615) | 0.00325  (0.00419) | 0.00155  (0.00885) |
|  |  |  |  |  |  |  |  |
| Lagged financial motivation (between) | -0.01937  (0.03357) | -0.00170  (0.00402) | 0.09720  (0.26818) | 0.22298  (0.30958) | 0.02412***  (0.00737) | 0.02481***  (0.00759) | 0.00333  (0.00845) |
|  |  |  |  |  |  |  |  |
| Lagged patient-oriented altruism (between) | 0.04100  (0.05704) | 0.00236  (0.00656) | 1.79684***  (0.48927) | 1.12836**  (0.46596) | -0.03399***  (0.01296) | -0.03723***  (0.01359) | -0.01878  (0.01402) |
|  |  |  |  |  |  |  |  |
| List per GP (within) | 0.00001 | 0.00000 | -0.00008 | -0.00003 | -0.00000 | -0.00000 | 0.00000 |
|  | (0.00001) | (0.00000) | (0.00009) | (0.00009) | (0.00000) | (0.00000) | (0.00000) |
| List per GP (between) | 0.00002 | 0.00000** | 0.00053*** | -0.00005 | -0.00000 | 0.00000 | -0.00000 |
|  | (0.00002) | (0.00000) | (0.00017) | (0.00013) | (0.00000) | (0.00000) | (0.00000) |
| SOCIO1 (within) | -1.78553 | 0.20101 | 10.60398 | -33.71604 | 1.17885** | -1.81095*** | -0.08030 |
|  | (3.00036) | (0.36652) | (23.17458) | (25.99585) | (0.58325) | (0.54667) | (0.61741) |
| SOCIO2 (within) | 3.24513*** | 0.35529*** | 17.28102*** | -13.29597*** | 0.13142 | -0.08499 | -0.01209 |
|  | (0.55311) | (0.06181) | (4.15819) | (4.03864) | (0.10164) | (0.13124) | (0.12536) |
| SOCIO3 (within) | 0.32039 | -0.00587 | -8.05970 | 60.81504*** | -0.28479*** | 0.10696 | -0.35927*** |
|  | (0.80598) | (0.09690) | (5.78755) | (10.34499) | (0.09173) | (0.12422) | (0.13135) |
| SOCIO4 (within) | 1.95840 | 0.26623 | 17.80791 | -26.92376 | 0.11673 | 0.26127 | 0.50413 |
|  | (2.18678) | (0.25528) | (15.99621) | (19.65386) | (0.38110) | (0.42354) | (0.46835) |
| SOCIO5 (within) | 0.32612* | 0.03525* | -1.72661 | -4.04872*** | 0.00474 | 0.00820 | -0.00611 |
|  | (0.17297) | (0.01996) | (1.40593) | (1.53827) | (0.03797) | (0.03857) | (0.03777) |
| SOCIO6 (within) | -2.34735*** | -0.24912*** | -1.18462 | 5.44629* | 0.07119 | -0.24635*** | 0.08250 |
|  | (0.33103) | (0.03802) | (2.89450) | (2.98031) | (0.06012) | (0.08254) | (0.08090) |
| SOCIO7 (within) | 3.55424*** | 0.48265*** | 17.87395*** | -14.11906*** | -0.15112 | 0.07426 | 0.05049 |
|  | (0.57934) | (0.06708) | (5.17538) | (4.94050) | (0.10953) | (0.15022) | (0.13631) |
| CCI = 1 (within) | 10.90431*** | 1.14752*** | -3.03842 | 1.62177 | -0.25491 | 0.93506*** | 1.14097*** |
|  | (1.08906) | (0.12355) | (9.13473) | (9.92316) | (0.18586) | (0.31945) | (0.23585) |
| CCI > 1 (within) | 2.78945*** | 0.19078* | 8.89710 | 32.30294*** | 0.20760 | 0.90649*** | -0.22662 |
|  | (0.92628) | (0.10524) | (8.07926) | (8.34518) | (0.18868) | (0.26230) | (0.22008) |
| SOCIO1 (between) | 1.45464 | 0.11072 | 6.90933 | 4.09044 | -0.36074 | -0.22766 | 0.67428* |
|  | (1.35762) | (0.24738) | (9.27971) | (8.83126) | (0.38188) | (0.18356) | (0.39535) |
| SOCIO2 (between) | -1.19692 | -0.00753 | 2.53583 | 0.61829 | 0.20545 | -0.19370 | -0.06962 |
|  | (0.80614) | (0.11691) | (6.58662) | (6.30345) | (0.22174) | (0.14590) | (0.25263) |
| SOCIO3 (between) | 0.05415 | -0.22777 | 1.42000 | 8.94931 | -0.21219 | -0.32962* | 0.24928 |
|  | (1.08208) | (0.16672) | (7.54541) | (7.36572) | (0.28226) | (0.19404) | (0.32565) |
| SOCIO4 (between) | 5.27654** | 0.50415 | -21.57934 | 11.38928 | -0.12683 | -0.27100 | 0.05218 |
|  | (2.25506) | (0.35237) | (17.53082) | (16.45160) | (0.49706) | (0.35851) | (0.75628) |
| SOCIO5 (between) | 0.52329 | -0.02883 | 20.70139*** | -6.80831 | -0.17208 | -0.03426 | -0.24436 |
|  | (0.76171) | (0.10480) | (6.21852) | (4.93812) | (0.16894) | (0.12518) | (0.14974) |
| SOCIO6 (between) | 0.03531 | -0.11366 | -7.08466 | 8.59800* | -0.09998 | 0.17378* | -0.07455 |
|  | (0.48717) | (0.08584) | (4.69409) | (4.76153) | (0.13817) | (0.09331) | (0.20030) |
| SOCIO7 (between) | 2.81012*** | 0.13926 | 9.81565 | 4.90584 | -0.42608** | 0.02947 | -0.42591 |
|  | (0.73249) | (0.12147) | (6.90038) | (7.78818) | (0.18707) | (0.15406) | (0.29511) |
| CCI = 1 (between) | 1.90699 | 0.32635* | 1.73314 | -22.35011** | 0.17708 | 0.54669** | -0.07338 |
|  | (1.18919) | (0.17809) | (9.52341) | (9.85446) | (0.31173) | (0.23486) | (0.43302) |
| CCI > 1 (between) | -0.04526 | 0.13815 | 2.44759 | -5.93992 | 0.37139 | 0.16971 | -0.06653 |
|  | (0.89439) | (0.13466) | (7.01011) | (8.05253) | (0.23834) | (0.17079) | (0.31071) |
| 2019 (within) | -0.19612*** | -0.02174*** | 0.79332*** | -1.13052*** | 0.01193*** | -0.02155*** | 0.02012*** |
|  | (0.00901) | (0.00152) | (0.08540) | (0.08309) | (0.00290) | (0.00194) | (0.00317) |
| 2020 (within) | -0.13806*** | -0.01436*** | 0.41359*** | -0.87944*** | 0.01061*** | -0.00986*** | -0.06001*** |
|  | (0.01186) | (0.00209) | (0.09609) | (0.09511) | (0.00363) | (0.00205) | (0.00453) |
| 2021 (within) | -0.06194*** | -0.00643*** | 0.21746*** | -0.25070*** | 0.00202 | -0.00988*** | -0.00981*** |
|  | (0.00658) | (0.00106) | (0.05557) | (0.05372) | (0.00204) | (0.00117) | (0.00250) |
| 2019 (between) | -0.20814*** | -0.02302*** | 1.13803*** | -0.71105** | 0.00311 | -0.02527*** | 0.01897** |
|  | (0.03371) | (0.00402) | (0.27625) | (0.31617) | (0.00602) | (0.00774) | (0.00787) |
| 2020 (between) | -0.17954*** | -0.02191*** | 0.50229 | -0.94490*** | -0.00741 | 0.00174 | -0.03584*** |
|  | (0.04651) | (0.00569) | (0.33278) | (0.36241) | (0.00786) | (0.00963) | (0.01034) |
| 2021 (between) | -0.04605 | -0.00530 | 0.15695 | -0.17569 | -0.00026 | 0.00384 | 0.00371 |
|  | (0.03972) | (0.00476) | (0.28613) | (0.31591) | (0.00700) | (0.00824) | (0.00917) |
| Region North | 0.37333*** | 0.03501*** | -0.76014*** | -2.00087*** | -0.02142*** | 0.06001*** | 0.03611*** |
|  | (0.03462) | (0.00413) | (0.24397) | (0.31461) | (0.00548) | (0.00744) | (0.00763) |
| Region Central | 0.32486*** | 0.03174*** | -0.88227*** | -1.07672*** | 0.00015 | 0.04411*** | 0.04002*** |
|  | (0.02958) | (0.00345) | (0.20248) | (0.31012) | (0.00464) | (0.00551) | (0.00611) |
| Region South | 0.15210*** | 0.01213*** | -0.81352*** | -2.32873*** | -0.01848*** | 0.03355*** | 0.03329*** |
|  | (0.02671) | (0.00309) | (0.20377) | (0.26865) | (0.00433) | (0.00579) | (0.00638) |
| Region Zealand | 0.12281*** | 0.01267*** | 0.48946** | -0.62921** | -0.02238*** | 0.01494** | 0.00004 |
|  | (0.02537) | (0.00279) | (0.22015) | (0.24449) | (0.00456) | (0.00701) | (0.00598) |
| Rural area | -0.04434*** | -0.00373** | 0.12997 | 0.18680 | -0.00507* | -0.01030** | -0.00628 |
|  | (0.01583) | (0.00180) | (0.13030) | (0.12859) | (0.00273) | (0.00410) | (0.00441) |
| Urban area | 0.00952 | 0.00054 | 0.09642 | 0.47055*** | 0.00132 | -0.00849* | -0.01963*** |
|  | (0.02012) | (0.00222) | (0.15071) | (0.15953) | (0.00368) | (0.00475) | (0.00463) |
| Risk of GP shortage | -0.06481** | -0.00739** | 0.24942 | -0.64067*** | -0.00202 | 0.01088 | -0.00297 |
|  | (0.02595) | (0.00308) | (0.25304) | (0.24267) | (0.00532) | (0.00744) | (0.00641) |
| Constant | 2.02325*** | 0.06498*** | -1.65465** | 1.73255*** | 0.06610*** | 0.12458*** | 0.12976*** |
|  | (0.07409) | (0.00870) | (0.64907) | (0.61342) | (0.01489) | (0.01778) | (0.01646) |
|  |  |  |  |  |  |  |  |
| Observations | 3,286 | 3,286 | 3,286 | 3,286 | 3,286 | 3,286 | 3,286 |
| No. of practices | 1,248 | 1,248 | 1,248 | 1,248 | 1,248 | 1,248 | 1,248 |

Note: The columns contain coefficients from within-between regressions estimated by maximum likelihood. Each of the seven quality indicators (calculated over the same half-year window as the survey data collection for each year 2019 to 2022) is regressed on financial motivation and patient-oriented altruism from each year (2019 to 2022), time dummies (reference year 2023) and a set of controls related to patient, practice, and area characteristics (see Appendix C for details). The regressions are based on an unbalanced sample of 3286 observations from 1248 general practices in Denmark. Observations are weighted according to the share of GPs in the practice responding to the motivation survey in that year. The number of observations per wave is 755 (in 2019), 842 (in 2020), 841 (in 2021), and 848 (in 2022). Standard errors clustered by practice in parentheses.

*p<0.10	**p<0.05	*** p<0.01

# **Appendix Q – Unadjusted regression results with motivations and time dummies as controls**

Table Q1. Unadjusted regression results with motivations and time dummies as controls

|  | (1) | (2) | (3) | (4) | (5) | (6) | (7) |
| --- | --- | --- | --- | --- | --- | --- | --- |
|  | Average number of prescriptions (over 65) | Share of polypharmacy patients  (over 65) | Average DDDs, NSAIDs without PPI (over 65) | Average DDDs, sleeping medication (18+) | Share of patients with preventive home visit (75+) | Share of patients with annual control for chronic disease (18+) | Share of COPD and asthma patients with spirometry test (18+) |
|  |  |  |  |  |  |  |  |
| Lagged financial motivation (within) | -0.01303  (0.01628) | -0.00062  (0.00280) | -0.03928  (0.13391) | -0.31721**  (0.12980) | 0.00369  (0.00409) | 0.01150***  (0.00406) | -0.00341  (0.00524) |
|  |  |  |  |  |  |  |  |
| Lagged patient-oriented altruism (within) | 0.01636  (0.02240) | -0.00101  (0.00349) | -0.13148  (0.17557) | 0.04510  (0.15914) | 0.00686  (0.00799) | 0.00662  (0.00612) | 0.00476  (0.00733) |
|  |  |  |  |  |  |  |  |
| Lagged financial motivation (between) | -0.09559*  (0.05002) | -0.01115**  (0.00563) | 0.52893*  (0.29094) | 0.21180  (0.29929) | 0.03620***  (0.00809) | 0.00978  (0.00885) | -0.00298  (0.00810) |
|  |  |  |  |  |  |  |  |
| Lagged patient-oriented altruism (between) | 0.14554*  (0.08323) | 0.01705*  (0.00898) | 2.15143***  (0.47709) | 0.69517  (0.49114) | -0.02949**  (0.01172) | -0.02014  (0.01473) | -0.01060  (0.01285) |
|  |  |  |  |  |  |  |  |
| 2020 (within) | -0.27636*** | -0.02855*** | 0.46090*** | -1.19545*** | -0.00055 | -0.03807*** | -0.02960*** |
|  | (0.00618) | (0.00096) | (0.04869) | (0.05219) | (0.00164) | (0.00137) | (0.00197) |
| 2021 (within) | -0.16099*** | -0.01686*** | 0.19031*** | -0.67612*** | 0.00317* | -0.02485*** | -0.03327*** |
|  | (0.00558) | (0.00089) | (0.04632) | (0.04195) | (0.00172) | (0.00116) | (0.00193) |
| 2022 (within) | -0.11438*** | -0.01118*** | 0.11468*** | -0.33852*** | 0.00113 | -0.01633*** | -0.00198 |
|  | (0.00498) | (0.00080) | (0.03850) | (0.03691) | (0.00139) | (0.00093) | (0.00147) |
| 2020 (between) | -0.19976*** | -0.02220*** | 1.07109*** | -1.26997*** | -0.00860 | -0.03970*** | -0.03489*** |
|  | (0.04662) | (0.00510) | (0.26583) | (0.43464) | (0.00581) | (0.00786) | (0.00704) |
| 2021 (between) | -0.20826*** | -0.02110*** | 0.77755*** | -1.36826*** | 0.00199 | -0.03254*** | -0.03284*** |
|  | (0.04940) | (0.00556) | (0.25897) | (0.43707) | (0.00630) | (0.00842) | (0.00775) |
| 2022 (between) | -0.04245 | -0.00478 | 0.33733 | -0.65066 | -0.00141 | -0.00791 | 0.00195 |
|  | (0.05208) | (0.00587) | (0.26450) | (0.49314) | (0.00658) | (0.00939) | (0.00816) |
| Constant | 3.00545*** | 0.16654*** | 0.29221 | 4.45646*** | 0.05467*** | 0.18209*** | 0.15148*** |
|  | (0.06870) | (0.00738) | (0.40130) | (0.61467) | (0.00962) | (0.01192) | (0.01093) |
|  |  |  |  |  |  |  |  |
| Observations | 3,286 | 3,286 | 3,286 | 3,286 | 3,286 | 3,286 | 3,286 |
| No. of practices | 1,248 | 1,248 | 1,248 | 1,248 | 1,248 | 1,248 | 1,248 |

Note: The columns contain coefficients from within-between regressions estimated by maximum likelihood (MLE). Each of the seven quality indicators (calculated over the first six months of each year t=2020 to 2023) is regressed on financial motivation and patient-oriented altruism from each year t-1 (2019 to 2022) and time dummies (reference year 2023). Regressions are based on an unbalanced sample of 3286 observations from 1248 general practices in Denmark. The number of observations per wave is 755 (2020), 842 (2021), 841 (2022), and 848 (2023). Observations are weighted according to the share of GPs in the practice responding to the motivation survey in that year. Standard errors clustered at the practice level in parentheses.

*p<0.10	**p<0.05	*** p<0.01

# **Appendix R – Regression results when patient characteristics are removed from the model**

Table R1. Regression results when patient characteristics are removed from the model

|  | (1) | (2) | (3) | (4) | (5) | (6) | (7) |
| --- | --- | --- | --- | --- | --- | --- | --- |
|  | Average number of prescriptions (over 65) | Share of polypharmacy patients  (over 65) | Average DDDs, NSAIDs without PPI (over 65) | Average DDDs, sleeping medication (18+) | Share of patients with preventive home visit (75+) | Share of patients with annual control for chronic disease (18+) | Share of COPD and asthma patients with spirometry test (18+) |
|  |  |  |  |  |  |  |  |
| Lagged financial motivation (within) | -0.01256  (0.01626) | -0.00053  (0.00279) | -0.04150  (0.13395) | -0.31919**  (0.12974) | 0.00358  (0.00405) | 0.01152***  (0.00405) | -0.00316  (0.00525) |
|  |  |  |  |  |  |  |  |
| Lagged patient-oriented altruism (within) | 0.01711  (0.02251) | -0.00086  (0.00351) | -0.13045  (0.17624) | 0.04853  (0.15922) | 0.00641  (0.00792) | 0.00601  (0.00612) | 0.00466  (0.00733) |
|  |  |  |  |  |  |  |  |
| Lagged financial motivation (between) | -0.04503  (0.04588) | -0.00686  (0.00529) | 0.51180*  (0.27068) | 0.06242  (0.28507) | 0.02854***  (0.00765) | 0.02379***  (0.00779) | 0.00306  (0.00768) |
|  |  |  |  |  |  |  |  |
| Lagged patient-oriented altruism (between) | 0.08580  (0.07642) | 0.01179  (0.00849) | 2.06106***  (0.44752) | 1.02549**  (0.46839) | -0.01833*  (0.01100) | -0.03837***  (0.01328) | -0.01946  (0.01228) |
|  |  |  |  |  |  |  |  |
| List per GP (within) | 0.00001 | 0.00000 | 0.00004 | 0.00005 | -0.00001 | -0.00001*** | -0.00001 |
|  | (0.00002) | (0.00000) | (0.00010) | (0.00010) | (0.00001) | (0.00000) | (0.00000) |
| List per GP (between) | -0.00001 | 0.00000 | 0.00038*** | -0.00045 | 0.00000 | -0.00000 | 0.00000 |
|  | (0.00002) | (0.00000) | (0.00012) | (0.00029) | (0.00000) | (0.00000) | (0.00000) |
| 2020 (within) | -0.27602*** | -0.02848*** | 0.46129*** | -1.19473*** | -0.00078 | -0.03822*** | -0.02962*** |
|  | (0.00617) | (0.00096) | (0.04898) | (0.05224) | (0.00161) | (0.00136) | (0.00196) |
| 2021 (within) | -0.16072*** | -0.01681*** | 0.19137*** | -0.67580*** | 0.00301* | -0.02498*** | -0.03331*** |
|  | (0.00557) | (0.00089) | (0.04638) | (0.04196) | (0.00167) | (0.00115) | (0.00192) |
| 2022 (within) | -0.11404*** | -0.01112*** | 0.11506*** | -0.33885*** | 0.00092 | -0.01646*** | -0.00200 |
|  | (0.00498) | (0.00080) | (0.03884) | (0.03696) | (0.00133) | (0.00091) | (0.00147) |
| 2020 (between) | -0.22596*** | -0.02512*** | 0.99726*** | -1.24586*** | -0.00528 | -0.04398*** | -0.03620*** |
|  | (0.04129) | (0.00471) | (0.25281) | (0.40606) | (0.00554) | (0.00717) | (0.00678) |
| 2021 (between) | -0.19502*** | -0.01982*** | 0.63890*** | -1.35302*** | 0.00268 | -0.03072*** | -0.02900*** |
|  | (0.04481) | (0.00526) | (0.24257) | (0.41001) | (0.00594) | (0.00764) | (0.00744) |
| 2022 (between) | -0.08265* | -0.00887 | 0.38749 | -0.68649 | 0.00115 | -0.01389* | -0.00088 |
|  | (0.04693) | (0.00551) | (0.25447) | (0.50610) | (0.00635) | (0.00830) | (0.00786) |
| Region North | 0.30517*** | 0.02944*** | -0.51213*** | -1.01395*** | -0.02558*** | 0.05075*** | 0.02330*** |
|  | (0.02997) | (0.00349) | (0.17872) | (0.16294) | (0.00371) | (0.00642) | (0.00612) |
| Region Central | 0.27558*** | 0.02803*** | -0.83504*** | 0.01369 | -0.00397 | 0.02879*** | 0.01735*** |
|  | (0.02593) | (0.00287) | (0.11328) | (0.19629) | (0.00346) | (0.00404) | (0.00388) |
| Region South | 0.22308*** | 0.02080*** | -0.45034*** | -1.10038*** | -0.02050*** | 0.03530*** | 0.02095*** |
|  | (0.02345) | (0.00263) | (0.12302) | (0.13469) | (0.00334) | (0.00453) | (0.00418) |
| Region Zealand | 0.06904** | 0.00621** | 0.41820** | 0.15427 | -0.02134*** | 0.01474*** | -0.01436*** |
|  | (0.02694) | (0.00308) | (0.17854) | (0.17918) | (0.00349) | (0.00555) | (0.00441) |
| Rural area | -0.01243 | -0.00035 | 0.31269*** | 0.12578 | -0.00707*** | -0.00447 | -0.00029 |
|  | (0.01977) | (0.00228) | (0.11680) | (0.17083) | (0.00242) | (0.00389) | (0.00375) |
| Urban area | -0.06836*** | -0.00536** | 0.13081 | -0.09716 | 0.00766*** | -0.03124*** | -0.01972*** |
|  | (0.02179) | (0.00242) | (0.10295) | (0.14112) | (0.00293) | (0.00365) | (0.00341) |
| Risk of GP shortage | 0.11589*** | 0.01308*** | 0.71561*** | 0.11242 | -0.00826** | 0.01950*** | 0.00553 |
|  | (0.03161) | (0.00352) | (0.22748) | (0.18391) | (0.00336) | (0.00663) | (0.00524) |
| Constant | 2.91923*** | 0.15510*** | -0.14893 | 5.33259*** | 0.05382*** | 0.18691*** | 0.15253*** |
|  | (0.07200) | (0.00784) | (0.41054) | (0.98959) | (0.01105) | (0.01263) | (0.01187) |
|  |  |  |  |  |  |  |  |
| Observations | 3,286 | 3,286 | 3,286 | 3,286 | 3,286 | 3,286 | 3,286 |
| No. of practices | 1,248 | 1,248 | 1,248 | 1,248 | 1,248 | 1,248 | 1,248 |

Note: The columns contain coefficients from within-between regressions estimated by maximum likelihood. Each of the seven quality indicators (calculated over the first six months of each year t=2020 to 2023) is regressed on financial motivation and patient-oriented altruism from each year t-1 (2019 to 2022), time dummies (reference year 2023) and a set of controls related to practice and area characteristics (see Appendix C for details). Patient characteristics related to health and socioeconomic status are excluded. The regressions are based on an unbalanced sample of 3286 observations from 1248 general practices in Denmark. Observations are weighted according to the share of GPs in the practice responding to the motivation survey in that year. The number of observations per wave is 755 (in 2020), 842 (in 2021), 841 (in 2022), and 848 (in 2023). Standard errors clustered by practice in parentheses.

*p<0.10	**p<0.05	*** p<0.01

# **Appendix S – Regression results after entropy balancing on financial motivation**

Table S1. Regression results after entropy balancing on the first and second moments of all observable characteristics (balancing on financial motivation)

|  | (1) | (2) | (3) | (4) | (5) | (6) | (7) |
| --- | --- | --- | --- | --- | --- | --- | --- |
|  | Average number of prescriptions (over 65) | Share of polypharmacy patients  (over 65) | Average DDDs, NSAIDs without PPI (over 65) | Average DDDs, sleeping medication (18+) | Share of patients with preventive home visit (75+) | Share of patients with annual control for chronic disease (18+) | Share of COPD and asthma patients with spirometry test (18+) |
|  |  |  |  |  |  |  |  |
| Lagged financial motivation (within) | -0.01817  (0.01599) | -0.00150  (0.00283) | -0.00484  (0.14096) | -0.39350***  (0.12889) | 0.00304  (0.00455) | 0.01198***  (0.00373) | -0.00212  (0.00550) |
|  |  |  |  |  |  |  |  |
| Lagged patient-oriented altruism (within) | 0.01398  (0.02279) | -0.00248  (0.00344) | -0.22708  (0.19873) | 0.04101  (0.15291) | 0.00630  (0.00857) | 0.00703  (0.00557) | 0.00422  (0.00768) |
|  |  |  |  |  |  |  |  |
| Lagged financial motivation (between) | -0.02887  (0.03490) | -0.00439  (0.00406) | 0.56474**  (0.25162) | 0.26049  (0.25110) | 0.02500***  (0.00796) | 0.02580***  (0.00725) | 0.00403  (0.00794) |
|  |  |  |  |  |  |  |  |
| Lagged patient-oriented altruism (between) | 0.05155  (0.06267) | 0.00706  (0.00705) | 1.83822***  (0.43362) | 0.85576**  (0.40582) | -0.01727  (0.01136) | -0.03765***  (0.01308) | -0.01785  (0.01243) |
|  |  |  |  |  |  |  |  |
| List per GP (within) | 0.00001 | 0.00000 | 0.00002 | 0.00009 | -0.00000 | -0.00001** | -0.00000 |
|  | (0.00002) | (0.00000) | (0.00009) | (0.00008) | (0.00001) | (0.00000) | (0.00000) |
| List per GP (between) | 0.00001 | 0.00000 | 0.00037*** | -0.00003 | 0.00000 | 0.00000 | 0.00000 |
|  | (0.00001) | (0.00000) | (0.00011) | (0.00010) | (0.00000) | (0.00000) | (0.00000) |
| SOCIO1 (within) | 2.63453* | 0.45308** | -2.08017 | -14.77115 | 0.60479 | 0.12870 | 0.76019* |
|  | (1.57341) | (0.21552) | (13.45275) | (9.23890) | (0.43686) | (0.23574) | (0.40352) |
| SOCIO2 (within) | 1.37042* | 0.24045* | -5.00040 | -3.00432 | 0.64276* | -0.09918 | -0.07430 |
|  | (0.81049) | (0.13513) | (7.80781) | (5.63521) | (0.33622) | (0.14614) | (0.25951) |
| SOCIO3 (within) | -2.37869** | -0.29080* | 4.16849 | 5.46119 | -0.39340 | -0.37321* | -0.10237 |
|  | (1.12231) | (0.15659) | (8.42566) | (8.69133) | (0.28025) | (0.20155) | (0.29624) |
| SOCIO4 (within) | -1.88864 | -0.32823 | -22.48382 | -20.53782 | -0.79529 | -0.47543 | -0.74380 |
|  | (2.33711) | (0.35021) | (17.35653) | (16.54653) | (0.88270) | (0.43617) | (0.73903) |
| SOCIO5 (within) | 0.66743 | 0.11948 | 9.50923 | 2.20349 | 0.16599 | -0.06287 | 0.05041 |
|  | (0.63723) | (0.10596) | (6.75061) | (4.15935) | (0.28973) | (0.11495) | (0.15482) |
| SOCIO6 (within) | 0.46860 | -0.09086 | 18.58867*** | 1.49720 | -0.07326 | 0.08950 | 0.00591 |
|  | (0.58614) | (0.09774) | (6.10161) | (4.90709) | (0.22192) | (0.12269) | (0.18378) |
| SOCIO7 (within) | 2.93702*** | 0.42206*** | -5.54681 | 21.89596*** | 0.21918 | 0.33429* | 0.09066 |
|  | (0.80225) | (0.11721) | (6.17249) | (7.04318) | (0.23578) | (0.17488) | (0.27263) |
| CCI = 1 (within) | 1.86965 | 0.10838 | -12.90072 | -14.11969 | -0.11063 | 0.68092** | 0.61213 |
|  | (1.15096) | (0.19012) | (9.32193) | (11.00479) | (0.31924) | (0.27868) | (0.41263) |
| CCI > 1 (within) | 3.12617*** | 0.54929*** | -15.75251* | 15.70296** | 0.39851 | 0.11331 | 0.94570*** |
|  | (0.89344) | (0.15026) | (8.34838) | (7.50912) | (0.28257) | (0.20844) | (0.32984) |
| SOCIO1 (between) | -1.78716 | 0.23568 | -13.64910 | -4.97979 | 0.50446 | -0.84334 | 0.59074 |
|  | (3.43174) | (0.38820) | (21.41878) | (19.32127) | (0.59824) | (0.58963) | (0.58905) |
| SOCIO2 (between) | 3.07940*** | 0.31036*** | 15.55656*** | -14.05727*** | 0.15917 | -0.13194 | -0.04186 |
|  | (0.62538) | (0.07334) | (3.97579) | (3.83649) | (0.09965) | (0.11799) | (0.11516) |
| SOCIO3 (between) | 2.69272*** | 0.29341*** | -0.49583 | 28.39739*** | -0.26916** | 0.20971 | -0.32797** |
|  | (0.77150) | (0.09064) | (5.23572) | (5.63498) | (0.11189) | (0.16789) | (0.16348) |
| SOCIO4 (between) | 0.38223 | 0.09365 | 23.19287 | 32.49606** | -0.34164 | 0.03989 | 0.16967 |
|  | (2.18537) | (0.24079) | (14.80098) | (14.74616) | (0.35159) | (0.46465) | (0.47315) |
| SOCIO5 (between) | 0.32810* | 0.02799 | -1.32627 | -2.49426** | 0.06075 | -0.00486 | 0.00161 |
|  | (0.17910) | (0.02060) | (1.20646) | (1.19357) | (0.03883) | (0.03632) | (0.03398) |
| SOCIO6 (between) | -2.14733*** | -0.21614*** | 0.15506 | 4.64198* | -0.00737 | -0.27637*** | -0.02019 |
|  | (0.36699) | (0.04395) | (2.48253) | (2.50476) | (0.06049) | (0.07350) | (0.07418) |
| SOCIO7 (between) | 3.75447*** | 0.51612*** | 17.98828*** | -10.31697** | -0.12140 | 0.19759 | 0.28303** |
|  | (0.59696) | (0.07558) | (4.71099) | (4.46954) | (0.10536) | (0.14642) | (0.13429) |
| CCI = 1 (between) | 10.67355*** | 1.12246*** | -13.91423* | 16.90419** | -0.05079 | 1.15556*** | 1.29912*** |
|  | (1.21970) | (0.15145) | (7.30665) | (8.55570) | (0.18322) | (0.25241) | (0.23594) |
| CCI > 1 (between) | 3.14610*** | 0.16160 | 6.02904 | 28.37938*** | 0.12140 | 0.75194*** | -0.26036 |
|  | (1.00746) | (0.13295) | (6.65163) | (8.03177) | (0.15887) | (0.21954) | (0.21008) |
| 2020 (within) | -0.27527*** | -0.02987*** | 0.69531*** | -0.90702*** | -0.00571 | -0.03559*** | -0.02936*** |
|  | (0.01434) | (0.00229) | (0.14103) | (0.11433) | (0.00538) | (0.00278) | (0.00467) |
| 2021 (within) | -0.15682*** | -0.01698*** | 0.34001*** | -0.49208*** | 0.00072 | -0.02342*** | -0.03223*** |
|  | (0.00880) | (0.00151) | (0.08376) | (0.07203) | (0.00294) | (0.00184) | (0.00310) |
| 2022 (within) | -0.10210*** | -0.00956*** | 0.18020*** | -0.28975*** | 0.00119 | -0.01536*** | 0.00155 |
|  | (0.00668) | (0.00108) | (0.05622) | (0.05038) | (0.00198) | (0.00123) | (0.00199) |
| 2020 (between) | -0.30390*** | -0.03738*** | 0.93160*** | -1.08409*** | -0.00985 | -0.04276*** | -0.04365*** |
|  | (0.04257) | (0.00501) | (0.30127) | (0.27808) | (0.00756) | (0.00888) | (0.00839) |
| 2021 (between) | -0.23380*** | -0.02708*** | 0.52426** | -0.98700*** | -0.00101 | -0.02583*** | -0.03331*** |
|  | (0.03801) | (0.00465) | (0.24304) | (0.22482) | (0.00663) | (0.00814) | (0.00827) |
| 2022 (between) | -0.13016*** | -0.01457*** | 0.31042 | -0.35512 | 0.00147 | -0.01634** | -0.00239 |
|  | (0.03669) | (0.00459) | (0.23840) | (0.24450) | (0.00676) | (0.00770) | (0.00775) |
| Region North | 0.31635*** | 0.02822*** | -0.84269*** | -1.35241*** | -0.01531*** | 0.05273*** | 0.03303*** |
|  | (0.03492) | (0.00416) | (0.21441) | (0.21531) | (0.00500) | (0.00748) | (0.00744) |
| Region Central | 0.30498*** | 0.02943*** | -1.10385*** | -0.33425* | 0.00386 | 0.03928*** | 0.03488*** |
|  | (0.02922) | (0.00340) | (0.17042) | (0.18838) | (0.00467) | (0.00603) | (0.00610) |
| Region South | 0.09293*** | 0.00554* | -0.93964*** | -1.72419*** | -0.01009** | 0.02733*** | 0.02728*** |
|  | (0.02573) | (0.00298) | (0.17138) | (0.17746) | (0.00470) | (0.00595) | (0.00633) |
| Region Zealand | 0.09569*** | 0.00933*** | 0.20039 | -0.23884 | -0.01661*** | 0.01858*** | -0.00045 |
|  | (0.02482) | (0.00286) | (0.18802) | (0.19040) | (0.00419) | (0.00616) | (0.00572) |
| Rural area | -0.05486*** | -0.00555*** | 0.11503 | 0.12003 | -0.00402 | -0.01016** | -0.00427 |
|  | (0.01690) | (0.00202) | (0.11752) | (0.11465) | (0.00261) | (0.00404) | (0.00404) |
| Urban area | 0.01354 | 0.00099 | 0.19195 | 0.56502*** | 0.00063 | -0.00837* | -0.01374*** |
|  | (0.02010) | (0.00228) | (0.12978) | (0.13910) | (0.00346) | (0.00441) | (0.00433) |
| Risk of GP shortage | -0.07566*** | -0.00910*** | 0.06596 | -0.45426** | -0.00359 | 0.00884 | -0.00027 |
|  | (0.02812) | (0.00305) | (0.21306) | (0.20192) | (0.00417) | (0.00678) | (0.00588) |
| Constant | 2.09599*** | 0.07502*** | -1.73780*** | 1.75133*** | 0.04718*** | 0.13287*** | 0.11702*** |
|  | (0.07658) | (0.00880) | (0.55613) | (0.52664) | (0.01362) | (0.01653) | (0.01562) |
|  |  |  |  |  |  |  |  |
| Observations | 3,286 | 3,286 | 3,286 | 3,286 | 3,286 | 3,286 | 3,286 |
| No. of practices | 1,248 | 1,248 | 1,248 | 1,248 | 1,248 | 1,248 | 1,248 |

Note: The columns contain coefficients from within-between regressions estimated by maximum likelihood. Each of the seven quality indicators (calculated over the first six months of each year t=2020 to 2023) is regressed on financial motivation and patient-oriented altruism from each year t-1 (2019 to 2022), time dummies (reference year 2023) and a set of controls related to patient, practice, and area characteristics (see Appendix C for details). The regressions are based on an unbalanced sample of 3286 observations from 1248 general practices in Denmark. We use entropy balancing (Hainmueller, 2012) to pre-process the data such that the mean and variance of all observable patient, practice, and area characteristics are equalised between practices with high (above-median) and low (below-median) financial motivation. We then multiply the resulting motivation-specific balancing weights with the existing weights (share of GPs in the practice who responded to the survey in that wave) to obtain updated within-between regression estimates. The number of observations per wave is 755 (in 2020), 842 (in 2021), 841 (in 2022), and 848 (in 2023). Standard errors clustered by practice in parentheses. *p<0.10	**p<0.05	*** p<0.01

# **Appendix T – Regression results after entropy balancing on patient-oriented altruism**

Table T1. Regression results after entropy balancing on the first and second moments of all observable characteristics (balancing on patient-oriented altruism)

|  | (1) | (2) | (3) | (4) | (5) | (6) | (7) |
| --- | --- | --- | --- | --- | --- | --- | --- |
|  | Average number of prescriptions (over 65) | Share of polypharmacy patients  (over 65) | Average DDDs, NSAIDs without PPI (over 65) | Average DDDs, sleeping medication (18+) | Share of patients with preventive home visit (75+) | Share of patients with annual control for chronic disease (18+) | Share of COPD and asthma patients with spirometry test (18+) |
|  |  |  |  |  |  |  |  |
| Lagged financial motivation (within) | -0.00487  (0.01665) | -0.00091  (0.00281) | -0.04947  (0.14274) | -0.33813**  (0.13500) | 0.00550  (0.00439) | 0.01011***  (0.00390) | -0.00397  (0.00563) |
|  |  |  |  |  |  |  |  |
| Lagged patient-oriented altruism (within) | 0.00420  (0.02142) | -0.00157  (0.00348) | -0.14470  (0.19938) | 0.10803  (0.16996) | 0.00308  (0.00876) | 0.00453  (0.00566) | 0.00362  (0.00799) |
|  |  |  |  |  |  |  |  |
| Lagged financial motivation (between) | -0.02419  (0.03491) | -0.00338  (0.00403) | 0.53825**  (0.25265) | 0.22220  (0.24963) | 0.02430***  (0.00755) | 0.02623***  (0.00730) | 0.00409  (0.00776) |
|  |  |  |  |  |  |  |  |
| Lagged patient-oriented altruism (between) | 0.04592  (0.06336) | 0.00776  (0.00702) | 1.82147***  (0.43210) | 0.90724**  (0.40677) | -0.01851  (0.01148) | -0.03948***  (0.01336) | -0.01660  (0.01221) |
|  |  |  |  |  |  |  |  |
| List per GP (within) | 0.00002 | 0.00000 | 0.00001 | 0.00011 | -0.00001 | -0.00001** | -0.00000 |
|  | (0.00001) | (0.00000) | (0.00009) | (0.00009) | (0.00001) | (0.00000) | (0.00000) |
| List per GP (between) | 0.00001 | 0.00000 | 0.00036*** | -0.00004 | 0.00000 | 0.00000 | 0.00000 |
|  | (0.00001) | (0.00000) | (0.00011) | (0.00010) | (0.00000) | (0.00000) | (0.00000) |
| SOCIO1 (within) | 1.60422 | 0.19695 | 1.88037 | -14.89802 | 0.68523 | -0.06327 | 0.50329 |
|  | (1.43710) | (0.21361) | (14.18628) | (10.28727) | (0.54866) | (0.24764) | (0.40488) |
| SOCIO2 (within) | 1.38363* | 0.21546* | -5.65009 | -6.55973 | 0.37962 | -0.09123 | -0.09295 |
|  | (0.81032) | (0.12069) | (8.52522) | (6.49110) | (0.29423) | (0.14886) | (0.27897) |
| SOCIO3 (within) | -1.02058 | -0.15400 | -0.23239 | 3.13043 | -0.55270* | -0.32700* | 0.03623 |
|  | (1.56979) | (0.17591) | (9.33437) | (9.32139) | (0.31498) | (0.19672) | (0.31786) |
| SOCIO4 (within) | -0.97335 | -0.14926 | -30.22739* | -13.35810 | -0.25538 | -0.15022 | -0.12194 |
|  | (2.37629) | (0.38142) | (17.14228) | (16.85688) | (0.67584) | (0.45968) | (0.77455) |
| SOCIO5 (within) | -0.08380 | 0.08155 | 10.61248 | 0.82233 | 0.14082 | -0.14997 | -0.12580 |
|  | (0.59829) | (0.10255) | (6.77579) | (5.39940) | (0.24512) | (0.11490) | (0.15980) |
| SOCIO6 (within) | -0.10636 | -0.08323 | 13.65887** | 7.95443 | -0.07211 | 0.06725 | -0.08133 |
|  | (0.62692) | (0.08990) | (5.99984) | (6.17199) | (0.20257) | (0.12689) | (0.20026) |
| SOCIO7 (within) | 2.90362*** | 0.38053*** | -6.42803 | 17.64216** | 0.05272 | 0.22763 | 0.06311 |
|  | (0.76280) | (0.11127) | (6.10619) | (7.82309) | (0.20133) | (0.18393) | (0.27561) |
| CCI = 1 (within) | 2.29803* | 0.36859* | -7.00062 | -18.04353 | -0.02287 | 0.71135*** | 0.82205** |
|  | (1.27675) | (0.19851) | (9.93793) | (11.91134) | (0.28704) | (0.25164) | (0.41431) |
| CCI > 1 (within) | 2.76986*** | 0.46591*** | -20.54929** | 13.09972 | 0.52207** | 0.19713 | 1.06357*** |
|  | (0.90975) | (0.14639) | (8.23846) | (10.15792) | (0.26594) | (0.23021) | (0.34751) |
| SOCIO1 (between) | -2.06145 | 0.17275 | -10.40584 | -1.75911 | 0.54993 | -1.03824* | 0.43276 |
|  | (3.46706) | (0.38551) | (21.72460) | (19.99125) | (0.59795) | (0.61945) | (0.59221) |
| SOCIO2 (between) | 3.14963*** | 0.32363*** | 15.54276*** | -14.88592*** | 0.15649* | -0.08961 | -0.00015 |
|  | (0.62340) | (0.07258) | (4.01279) | (3.86821) | (0.09439) | (0.12576) | (0.11517) |
| SOCIO3 (between) | 2.63408*** | 0.28188*** | -0.69484 | 28.66989*** | -0.27262** | 0.19319 | -0.36291** |
|  | (0.77389) | (0.08989) | (5.26421) | (5.58236) | (0.11304) | (0.17053) | (0.15860) |
| SOCIO4 (between) | 0.23340 | 0.10292 | 19.51071 | 29.51551** | -0.38823 | 0.13104 | 0.17926 |
|  | (2.18474) | (0.23885) | (14.75205) | (14.51506) | (0.34469) | (0.47779) | (0.46597) |
| SOCIO5 (between) | 0.32430* | 0.02523 | -1.14798 | -2.44365** | 0.06278 | -0.00638 | 0.00721 |
|  | (0.18182) | (0.02051) | (1.20445) | (1.18571) | (0.03925) | (0.03654) | (0.03326) |
| SOCIO6 (between) | -2.18070*** | -0.22052*** | 0.63418 | 4.94209** | 0.00225 | -0.29950*** | -0.02362 |
|  | (0.36726) | (0.04378) | (2.50876) | (2.49709) | (0.05942) | (0.07700) | (0.07407) |
| SOCIO7 (between) | 3.75652*** | 0.51509*** | 18.96029*** | -8.54898** | -0.12371 | 0.17250 | 0.23661* |
|  | (0.59982) | (0.07604) | (4.67834) | (4.35000) | (0.09951) | (0.14991) | (0.12971) |
| CCI = 1 (between) | 10.46631*** | 1.09875*** | -11.84159 | 19.50720** | -0.05054 | 0.98835*** | 1.19086*** |
|  | (1.19966) | (0.14890) | (7.55165) | (8.60124) | (0.17588) | (0.28024) | (0.23030) |
| CCI > 1 (between) | 3.17176*** | 0.16928 | 5.05286 | 25.74020*** | 0.13886 | 0.84371*** | -0.16867 |
|  | (1.00049) | (0.13181) | (6.68248) | (8.07692) | (0.15835) | (0.23421) | (0.20881) |
| 2020 (within) | -0.28524*** | -0.02944*** | 0.61864*** | -0.82009*** | -0.00457 | -0.03638*** | -0.03132*** |
|  | (0.01459) | (0.00215) | (0.14325) | (0.12087) | (0.00485) | (0.00300) | (0.00501) |
| 2021 (within) | -0.16230*** | -0.01688*** | 0.32336*** | -0.42951*** | 0.00280 | -0.02451*** | -0.03326*** |
|  | (0.00885) | (0.00144) | (0.08646) | (0.07999) | (0.00277) | (0.00198) | (0.00330) |
| 2022 (within) | -0.11071*** | -0.01051*** | 0.17985*** | -0.24052*** | 0.00312 | -0.01631*** | -0.00012 |
|  | (0.00666) | (0.00105) | (0.05960) | (0.05928) | (0.00199) | (0.00133) | (0.00210) |
| 2020 (between) | -0.29705*** | -0.03625*** | 0.87101*** | -1.07076*** | -0.00857 | -0.04153*** | -0.04268*** |
|  | (0.04294) | (0.00506) | (0.30961) | (0.28565) | (0.00720) | (0.00904) | (0.00842) |
| 2021 (between) | -0.22527*** | -0.02567*** | 0.48482** | -0.94558*** | 0.00040 | -0.02549*** | -0.03253*** |
|  | (0.03812) | (0.00468) | (0.24098) | (0.23118) | (0.00634) | (0.00821) | (0.00828) |
| 2022 (between) | -0.12666*** | -0.01410*** | 0.23670 | -0.30493 | 0.00218 | -0.01764** | -0.00309 |
|  | (0.03688) | (0.00456) | (0.23543) | (0.25299) | (0.00652) | (0.00776) | (0.00771) |
| Region North | 0.31532*** | 0.02833*** | -0.82732*** | -1.37547*** | -0.01493*** | 0.05280*** | 0.03611*** |
|  | (0.03470) | (0.00407) | (0.21600) | (0.21734) | (0.00505) | (0.00752) | (0.00738) |
| Region Central | 0.30527*** | 0.02964*** | -1.08202*** | -0.37099** | 0.00469 | 0.03828*** | 0.03629*** |
|  | (0.02920) | (0.00338) | (0.17133) | (0.18848) | (0.00475) | (0.00606) | (0.00608) |
| Region South | 0.09316*** | 0.00552* | -0.94258*** | -1.75990*** | -0.01108** | 0.02742*** | 0.02880*** |
|  | (0.02579) | (0.00298) | (0.17301) | (0.17576) | (0.00451) | (0.00602) | (0.00630) |
| Region Zealand | 0.09300*** | 0.00892*** | 0.22255 | -0.20329 | -0.01601*** | 0.01659** | -0.00092 |
|  | (0.02468) | (0.00285) | (0.18554) | (0.19189) | (0.00418) | (0.00650) | (0.00558) |
| Rural area | -0.05449*** | -0.00555*** | 0.10459 | 0.15134 | -0.00382 | -0.01030** | -0.00407 |
|  | (0.01692) | (0.00201) | (0.11586) | (0.11400) | (0.00266) | (0.00408) | (0.00403) |
| Urban area | 0.01315 | 0.00102 | 0.17638 | 0.56402*** | -0.00006 | -0.00784* | -0.01345*** |
|  | (0.02017) | (0.00225) | (0.12989) | (0.13969) | (0.00350) | (0.00443) | (0.00426) |
| Risk of GP shortage | -0.07523*** | -0.00927*** | 0.06511 | -0.46992** | -0.00436 | 0.00952 | 0.00218 |
|  | (0.02800) | (0.00308) | (0.21221) | (0.20177) | (0.00405) | (0.00714) | (0.00599) |
| Constant | 2.10326*** | 0.07396*** | -1.79135*** | 1.72179*** | 0.04577*** | 0.13607*** | 0.11213*** |
|  | (0.07667) | (0.00878) | (0.55548) | (0.52987) | (0.01348) | (0.01670) | (0.01519) |
|  |  |  |  |  |  |  |  |
| Observations | 3,286 | 3,286 | 3,286 | 3,286 | 3,286 | 3,286 | 3,286 |
| No. of practices | 1,248 | 1,248 | 1,248 | 1,248 | 1,248 | 1,248 | 1,248 |

Note: The columns contain coefficients from within-between regressions estimated by maximum likelihood. Each of the seven quality indicators (calculated over the first six months of each year t=2020 to 2023) is regressed on financial motivation and patient-oriented altruism from each year t-1 (2019 to 2022), time dummies (reference year 2023) and a set of controls related to patient, practice, and area characteristics (see Appendix C for details). The regressions are based on an unbalanced sample of 3286 observations from 1248 general practices in Denmark. We use entropy balancing (Hainmueller, 2012) to pre-process the data such that the mean and variance of all observable patient, practice, and area characteristics are equalised between practices with high (above-median) and low (below-median) patient-altruistic motivation. We then multiply the resulting motivation-specific balancing weights with the existing weights (share of GPs in the practice who responded to the survey in that wave) to obtain updated within-between regression estimates. The number of observations per wave is 755 (in 2020), 842 (in 2021), 841 (in 2022), and 848 (in 2023). Standard errors clustered by practice in parentheses. *p<0.10	**p<0.05	*** p<0.01

# **Appendix U – Results for an alternative definition of high quality NSAID prescribing**

Our primary measure of quality related to NSAID prescribing focuses on the prescription of NSAIDs to older patients (above 65) without a concurrent PPI prescription, aligning with the definition used in Pedersen et al. (2024). Based on clinical inputs and updated guidelines on NSAID prescribing to high-risk patients, this robustness check uses an alternate measure of quality for the subgroup of patients defined as being at high risk for upper gastrointestinal bleeding. Patients are classified into risk groups according to a model introduced in Petersen et al. (2020) which considers patients’ age, sex, history of ulcer diagnoses, and use of medications that are known to increase bleeding risk. We use a 5-year backward-looking period (4 years in 2020 due to data availability) to define history of ulcer diagnosis in the Danish National Patient Register. Medication use in a given year is based on the redemption of the relevant prescriptions during the 6-month windows in which quality is measured. These prescription redemptions are recorded in the Danish National Prescription Register.

Table U1. Regression results for NSAID prescribing to high-risk patients.

|  | (1) |
| --- | --- |
|  | Average DDDs, NSAIDs without PPI (high-risk patients) |
|  |  |
| Lagged financial motivation (within) | -0.21744  (0.14827) |
|  |  |
| Lagged patient-oriented altruism (within) | -0.28579  (0.21275) |
|  |  |
| Lagged financial motivation (between) | 0.66921**  (0.30536) |
|  |  |
| Lagged patient-oriented altruism (between) | 2.01110***  (0.51092) |
|  |  |
| List per GP (within) | 0.00002  (0.00010) |
| List per GP (between) | 0.00043***  (0.00014) |
| SOCIO1 (within) | -12.41013  (12.60781) |
| SOCIO2 (within) | -2.84634  (7.98561) |
| SOCIO3 (within) | 7.90705  (9.92506) |
| SOCIO4 (within) | -23.26577  (19.42262) |
| SOCIO5 (within) | 13.98992*  (7.24599) |
| SOCIO6 (within) | 13.77177**  (6.15804) |
| SOCIO7 (within) | -0.60213  (7.45499) |
| CCI = 1 (within) | -6.88546  (11.06180) |
| CCI > 1 (within) | -21.87054**  (8.54973) |
| SOCIO1 (between) | 12.21167  (26.33595) |
| SOCIO2 (between) | 18.99138***  (5.16087) |
| SOCIO3 (between) | -10.83982*  (6.46542) |
| SOCIO4 (between) | 35.83854**  (16.84506) |
| SOCIO5 (between) | -0.52615  (1.42096) |
| SOCIO6 (between) | -0.00619  (3.18802) |
| SOCIO7 (between) | 20.86079***  (5.91872) |
| CCI = 1 (between) | -13.85601  (9.67891) |
| CCI > 1 (between) | 12.32348  (8.98845) |
| 2020 (within) | 0.92874***  (0.13067) |
| 2021 (within) | 0.49322***  (0.08113) |
| 2022 (within) | 0.25872***  (0.06033) |
| 2020 (between) | 0.78822**  (0.36836) |
| 2021 (between) | 0.43726  (0.29236) |
| 2022 (between) | 0.30220  (0.28449) |
| Region North | -0.73274***  (0.26471) |
| Region Central | -0.73251***  (0.22149) |
| Region South | -0.73655*** |
|  | (0.21249) |
| Region Zealand | 0.41162* |
|  | (0.22234) |
| Rural area | 0.26070* |
|  | (0.14403) |
| Urban area | 0.17313 |
|  | (0.14892) |
| Risk of GP shortage | 0.19965 |
|  | (0.26643) |
| Constant | -2.35803*** |
|  | (0.66015) |
|  |  |
| Observations | 3,286 |
| No. of practices | 1,248 |

Note: The column contains coefficients from a within-between regression estimated by maximum likelihood (MLE). The number of DDDs of NSAIDs prescribed to high-risk patients without PPI (calculated over the first six months of each year t=2020 to 2023) is regressed on financial motivation and patient-oriented altruism from each year t-1 (2019 to 2022) and time dummies (reference year 2023). The regression is based on an unbalanced sample of 3286 observations from 1248 general practices in Denmark. The number of observations per wave is 755 (2020), 842 (2021), 841 (2022), and 848 (2023). Observations are weighted according to the share of GPs in the practice responding to the motivation survey in that year. Standard errors clustered at the practice level in parentheses.

*p<0.10	**p<0.05	*** p<0.01

# **References**

Danish Regions. (2018, June 27). *Ekstra penge til praktiserende læger i særlige områder er nu fordelt.* [Extra money for GPs in specific areas has now been distributed]. <https://www.regioner.dk/services/nyheder/2018/juni/ekstra-penge-til-praktiserende-laeger-i-saerlige-omraader-er-nu-fordelt/>

Hainmueller, J. (2012). Entropy Balancing for Causal Effects: A Multivariate Reweighting Method to Produce Balanced Samples in Observational Studies. *Political Analysis*, *20*(1), 25–46. <https://doi.org/10.1093/pan/mpr025>

Oxholm, A. S., Gyrd-Hansen, D., Jacobsen, C. B., Jensen, U. T., & Pedersen, L. B. (2024). The link between physician motivation and care. *The European Journal of Health Economics*, *25*(3), 525–537. <https://doi.org/10.1007/s10198-023-01605-7>

Pedersen, L. B., Andersen, M. K., Wehberg, S., Siersma, V., Søndergaard, J., Kousgaard, M. B., Due, T. D., Reventlow, S., Bro, F., & Waldorff, F. B. (2024). Clinical effects of accreditation in general practice: A pragmatic randomized controlled study. *Family Practice*, *42*(2), cmae049. <https://doi.org/10.1093/fampra/cmae049>

Petersen, J., Møller Hansen, J., de Muckadell, O. B. S., Dall, M., & Hallas, J. (2020). A model to predict the risk of aspirin/non-steroidal anti-inflammatory drugs-related upper gastrointestinal bleeding for the individual patient. *Basic & Clinical Pharmacology & Toxicology*, *126*(5), 437–443. <https://doi.org/10.1111/bcpt.13370>

Quan, H., Li, B., Couris, C. M., Fushimi, K., Graham, P., Hider, P., Januel, J.-M., & Sundararajan, V. (2011). Updating and Validating the Charlson Comorbidity Index and Score for Risk Adjustment in Hospital Discharge Abstracts Using Data From 6 Countries. *American Journal of Epidemiology*, *173*(6), 676–682. <https://doi.org/10.1093/aje/kwq433>

Statistics Denmark. (2025). *Degree of Urbanisation (DEGURBA)—Statistics Denmark, v1:2007*. <https://www.dst.dk/en/Statistik/dokumentation/nomenklaturer/degurba-dst>

Vedsted, P., & Sørensen, T. H. (2009, March). *Praksisscore på DAMP baseret på praksispopulationer i 2006.* [Practice scores on DAMP based on practice populations in 2006].

Yordanov, D., Oxholm, A. S., Gyrd-Hansen, D., & Pedersen, L. B. (2023). Mapping GPs’ motivation — it’s not all about the money: A nationwide cross-sectional survey study from Denmark. *British Journal of General Practice*, *73*(734), e687–e693. <https://doi.org/10.3399/BJGP.2022.0563>
